# Bond and Antibond Resonances: A Unified Framework for Singlet Biradical Character

**DOI:** 10.1002/chem.70968

**Published:** 2026-04-08

**Authors:** Daniel T. Gschwind, Jonas Bresien

**Affiliations:** ^1^ Institute of Chemistry University of Rostock Rostock Germany

**Keywords:** ab‐initio calculations, biradical character, biradicals, bond order, bonding theory

## Abstract

While the mathematical description of the electronic structure of biradical(oid)s is nowadays well understood, we feel that the chemical‐conceptual understanding of the structure and bonding, especially of singlet biradicaloids, remains underdeveloped. In particular, the degree of “biradical character” of singlet biradicaloids is discussed in the literature using a heterogeneous selection of indices, which are often not directly comparable and therefore hamper the development of a systematic understanding as well as comparison of different biradicaloid species. This paper aims to ameliorate this situation, by taking a holistic approach considering a wide variety of biradical indicators (including e.g. the LUNO occupancy, singlet‐triplet (ST) gap, bond order (BO) between the radical centers) and applying established computational methods (DFT, CCSD(T), CASSCF, NEVPT2, MRCI) to attain a unified description across the whole range of biradical character, from closed‐shell molecules to “perfect” (open‐shell) biradicals. We mainly focus on model systems of inorganic, four‐membered ring systems which are formally isolobal to S_2_N_2_ to discuss our results, but the general conclusions should hold for any structure with biradical character. Most notably, we suggest describing the interaction between the radical electrons in biradicaloids with low to moderate biradical character using bonds and antibonds for an intuitive chemical understanding of these species.

## Graphical abstract

The electronic structure of a variety of singlet biradicaloids may be described in the Lewis picture using single antibonds as the central bonding motif. In conjunction with a consistent set of biradical indicators, we can resolve some long‐standing disputes over the biradical character of molecules such as O_3_ and S_2_N_2_.

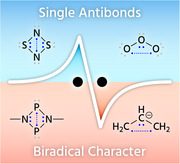

## 1 Introduction

Biradicals and biradicaloids (or diradical(oid)s, which is used synonymously) [1, 2] have fascinated chemists for over five decades [3, 4, 5, 6, 7, 8, 9, 10, 11, 12, 13, 14, 15, 16, 17, 18]. Contrary to common closed‐shell molecules, where all electrons are paired, biradical(oid)s are characterized by two electrons with no or only a weak interaction, leading to open‐shell character and therefore high reactivity as well as unusual properties of these species [8, 17]. For example, they may activate small molecules with a negligible activation barrier or exhibit nonlinear optic properties [19, 20, 21, 22]. Furthermore, biradical(oid)s often play a key role in photochemical processes and are of interest, for example, as molecular switches and for spin‐based information technology [10, 11, 23, 24, 25, 26].

**FIGURE 1:**
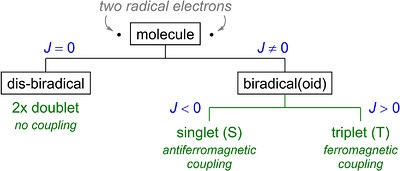
Two radical electrons can interact in different ways. The electron exchange coupling constant *J* is a measure for the electron interaction [9].

Yet, the term “biradical” actually refers to a variety of species with very different electronic structures and properties. This is best explained in terms of the exchange coupling constant *J*, which is used to express the strength of the interaction between the two radical electrons (Figure 1) [17]. In case *J*  =  0 (no interaction), the molecule might be regarded as a so‐called “two‐doublet” species, meaning that there are two individual radical centers in a single molecule, which behave like and cannot be distinguished from isolated radicals. The spins of the two radical electrons are not constrained in their orientation; thus, the lowest singlet and triplet state are degenerate. This situation is sometimes also referred to as “dis‐biradical” to imply that the radical electrons are often at a large distance from each other (*dis*: Latin for “apart”) [6, 9, 10].

In case *J* > 0 (ferromagnetic coupling), the two electrons prefer spin‐parallel alignment. Hence, the electronic ground state is a triplet state. Depending on the magnitude of the coupling, such biradicals can either behave similarly to dis‐biradicals (i.e., small *J* as found in many highly reactive organic biradicals) [15] or be somewhat less reactive due to strong coupling (i.e. large *J*). Triplet dioxygen certainly is a noteworthy example of the latter group, where the ferromagnetic coupling and resonance stabilization lead to a rather unreactive triplet biradicaloid [27].

Similarly, the case *J* < 0 (antiferromagnetic coupling) results in preferential antiparallel alignment of the electron spins. Again, if *J* is small, these singlet biradical(oid)s behave very similarly to dis‐biradicals, since only little energy is needed to overcome the interelectronic coupling, whereas with an increasing coupling constant, they gradually become classical closed‐shell species and therefore increasingly unreactive in radical reactions (which does not exclude high reactivity in closed‐shell reaction such as cycloadditions, though) [8, 9, 10].

In consequence, from an electronic spin point of view, we can distinguish between at least three categories of biradicals, namely dis‐biradicals with little to no interaction between the electrons as well as triplet and singlet biradicaloids with significant (anti)ferromagnetic coupling. However, the boundaries are diffuse and the transition between closed‐shell molecules, open‐shell singlet biradicaloids, dis‐biradicals, and triplet biradical(oid)s is continuous [8, 9, 10].

While triplet species may be accurately described by single‐determinantal methods (such as density functional theory, DFT) [28], dis‐biradicals and singlet biradicaloids require multi‐determinantal treatment to analyze their electronic structure and bonding [10]. This hampers the intuitive understanding of these species, since chemists are usually trained to think in terms of single configurations—that is, a single molecular orbital (MO) diagram—or preferably, even, in terms of Lewis resonance structures [9, 29]. While translation of a multi‐reference wavefunction (i.e., using a delocalized orbital basis) into a valence bond (VB)‐type wavefunction (using localized orbitals) is mathematically straightforward [8, 10, 15], its chemical interpretation is not, as the information about the biradical character becomes obscured in the process (vide infra). This may easily lead to misinterpretations and/or misunderstandings with respect to the electronic structure.

Additionally, while the *biradical character* of singlet biradical(oid)s is frequently discussed in the literature, different authors use different indicators of biradical character [3, 30, 31, 32, 33, 34, 35], rendering direct comparison between the results often impractical or even impossible. This may lead to confusion especially for nonexperts, which is why we would like to propose a unified, more intuitive concept of classifying biradical(oid)s, much in the same way that chemists use Lewis resonance structures to understand the electronic structure of molecules.

Before we start the discussion of our results, we would like to re‐iterate a few key points of the mathematical description of biradical states which are important for the understanding of this article. For a more in‐depth analysis, we would like to refer the interested reader to a selection of review articles on this topic [3, 8, 10, 11, 15].

**FIGURE 2:**
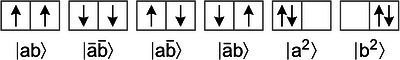
Graphical representation of the six possible configurations of two electrons in two orbitals *ψ*_a_ and *ψ*_b_. We use the notation |ab⟩ for $|\psi_{a}\psi_{b}\rangle$ etc.

In a biradical(oid), the two radical electrons are distributed over two orbitals (contrary to closed shell molecules, where the two energetically highest electrons are placed in a single orbital). Two electrons in two (delocalized) orbitals *ψ*_a_ and *ψ*_b_ can, in principle, form six possible configurations (see also Figure 2):

(1) $$\begin{array}{c} \left|\mathrm{ab}\rangle \right.,\;\left|\bar{a}\bar{b}\rangle \right.,\;\left|a\bar{b}\rangle \right.,\;\left|\bar{a}b\rangle \right.,\;\left|a^{2}\rangle \right.,\;\left|b^{2}\rangle \right.\; \end{array}$$

This results in four different electronic states, namely a triplet state with the three degenerate *M*_S_ = −1, 0, +1 configurations

(2) $$\begin{array}{c} \;\Psi_{T_{-1}}=\left|\bar{a}\bar{b}\rangle \right.\;,\;\;\Psi_{T_{0}}=\frac{1}{\sqrt{2}}\;\left(\left|a\bar{b}\rangle \right.+\left|\bar{a}b\rangle \right.\right),\;\\ \;\Psi_{T_{+1}}=\left|\mathrm{ab}\rangle \right.\;\; \end{array}$$

a low‐energy, two‐configuration singlet state

(3) $$\begin{array}{c} \;\Psi_{S_{-}}=c_{1}\;\left|a^{2}\rangle \right.-c_{2}\left|b^{2}\rangle \right.\; \end{array}$$

an “open‐shell” singlet state

(4) $$\begin{array}{c} \;\Psi_{S_{\mathrm{OS}}}=\frac{1}{\sqrt{2}}\;\left(\left|a\bar{b}\rangle \right.-\left|\bar{a}b\rangle \right.\right)\; \end{array}$$

and a high‐energy, two configuration singlet state

(5) $$\begin{array}{c} \;\Psi_{S_{+}}=c_{2}\;\left|a^{2}\rangle \right.+c_{1}\left|b^{2}\rangle \right.\; \end{array}$$

with the configuration interaction (CI) coefficients *c*_1_ ≥ *c*_2_ > 0 and *c*_1_^2^ + *c*_2_^2^  =  1. We do want to stress that all three singlet states are open‐shell singlets and are described by two configurations, even if the names, which are commonly used in the literature [8], suggest otherwise.

The relevant states regarding this article are the triplet state (usually expressed by $\Psi_{T_{+1}}$, although the other two configurations would be equally valid, eqn. 2) and the singlet state $\Psi_{S_{-}}$ (eqn. 3); the singlet‐triplet (ST) energy gap (ST gap, Δ*E*_ST_) is computed from the energies of those states:

(6) $$\begin{array}{c} \Delta \;E_{\mathrm{ST}}=E_{S}\;-E_{T}\; \end{array}$$

In a “perfect” biradical with degenerate frontier orbitals, the triplet state is typically the ground state; however, if there is an energy splitting between the orbitals *ψ*_a_ and *ψ*_b_, the singlet state $\Psi_{S_{-}}$ becomes the ground state (i.e., in a singlet biradicaloid, Δ*E*_ST_ is the S_0_‐T_1_ ST gap). The singlet biradical character of the singlet state is often derived from the CI coefficients, for example [3, 30].

(7) $$\begin{array}{c} \beta =2c_{2}^{2}\; \end{array}$$

However, the coefficients depend on the exact choice of orbitals [11]. Therefore, we prefer to use the LUNO occupation number because its definition is unambiguous (LUNO = Lowest Unoccupied Natural Orbital) [8, 32]. If the wavefunction is expressed in terms of the natural orbitals (NOs), LUNO occupancy and *β* are identical in the two‐electrons‐in‐two‐orbitals (2e2o) picture (i.e., for a CASSCF(2,2) wavefunction) [10]. As mentioned before, many more different indices for the quantization of biradical character have been proposed over the years, which depend, for example, on the CI coefficients (like *β*) or on various properties of broken‐symmetry wavefunctions [8].

Note that delocalized orbitals are unsuited to interpret the electronic structure in an intuitive chemical way. A valence bond (VB)‐type description of the wavefunction, which can be translated into Lewis resonance structures, may be obtained by localizing the orbitals [8, 10],

(8) $$\begin{array}{c} \;\chi_{1}=\frac{1}{\sqrt{2}}\;\left(\psi_{a}+\psi_{b}\right),\;\;\chi_{2}=\frac{1}{\sqrt{2}}\left(\psi_{a}-\psi_{b}\right)\; \end{array}$$

which results in an alternative, mathematically identical description of the singlet wavefunction using those localized orbitals *χ*_1_ and *χ*_2_:

(9) $$\begin{array}{c} \psi_{S\_}=\frac{c_{1}+c_{2}}{{\underset{︸}{\sqrt{2}}}_{c_{cov}}}\cdot \frac{1}{\sqrt{2}}\left(|1\bar{2}\rangle -|\bar{1}2\rangle \right)\;+\frac{c_{1}-c_{2}}{{\underset{︸}{\sqrt{2}}}_{c_{ion}}}\cdot \frac{1}{\sqrt{2}}\left(|1^{2}\rangle +|2^{2}\rangle \right) \end{array}$$

Clearly, a single electron may be found at each of the two different radical sites (cf. configurations $|1\bar{2}\rangle$ and $|\bar{1}2\rangle$) with a probability of *c*_cov_^2^, or both electrons may be found at the same radical site ($|1^{2}\rangle$, $|2^{2}\rangle$) with a probability of *c*_ion_^2^. This finding is often interpreted in terms of a “covalent” and “ionic” contribution to the wavefunction [8, 10]. Note, however, that a “closed‐shell”, covalent bond is represented by *c*_cov_ = *c*_ion_ = 1/√2, that is an equal mixture of “covalent” and “ionic” contributions, whereas the singlet state of a “perfect” biradical is described by the limit *c*_cov_ = 1 and *c*_ion_ = 0. The singlet biradical character is therefore not easily visible in the localized picture, but *both covalent and ionic contribution* must be considered, for example: [3, 8, 30]

(10) $$\begin{array}{c} \beta ={\left(c_{\mathrm{cov}}-c_{\mathrm{ion}}\right)}^{2}\;\; \end{array}$$

In particular, and this cannot be stressed enough, note that the configurations $|1\bar{2}\rangle$ and $|\bar{1}2\rangle$ alone do not say anything about biradical character, even though one might be tempted to interpret them as such since a single electron is localized in each of the localized orbitals 1 and 2. Notice, though, that this is the case even in a covalent bond, with a probability of 50% (in the closed‐shell limit).

Lastly, the triplet wavefunction becomes

(11) $$\begin{array}{c} \;\Psi_{T_{+1}}=\left|12\rangle \right.\;\; \end{array}$$

with a single electron in each of the localized orbitals [10].

The magnitude of interaction between the radical electrons (i.e., ferromagnetic or antiferromagnetic) cannot be derived from a single state alone, but is connected to the ST gap [17],

(12) $$\begin{array}{c} \Delta \;E_{\mathrm{ST}}=2J\; \end{array}$$

where *J* is the exchange coupling constant from the phenomenological Heisenberg‐Dirac‐Van Vleck Hamiltonian [36, 37, 38]

(13) $${\hat{H}}_{\text{HDvV}}=-2J{\hat{S}}_{1}{\hat{S}}_{2}$$

Since an alternative definition, ${\hat{H}}_{\mathrm{HDvV}}=-J{\hat{S}}_{1}{\hat{S}}_{2}$, is also frequently used in the literature [25], resulting in Δ*E*_ST_ = *J*, and also because the HDvV Hamiltonian only pertains to localized spins, we will, however, discuss only $\Delta E_{\mathrm{ST}}$ values to avoid ambiguities.

## 2 Computational Methods

For our unified description of biradical character, we investigated the following quantities for a variety of biradical(oid)s: the vertical ST gap as a measure of the (anti)ferromagnetic coupling between the electrons, the adiabatic ST gap that is relevant for thermal equilibria between the two states, the biradical character *β*  = 2*c*_2_^2^ derived from complete active space self‐consistent field [CASSCF(2,2)] calculations as well as the LUNO occupancies from *N*‐electron valence state perturbation (NEVPT2) and multi‐reference configuration interaction (MRCI) calculations, the bond order (BO) between the radical sites as derived from the CASSCF density matrix using localized orbitals, and the weight of important Lewis resonance structures.

**FIGURE 3:**
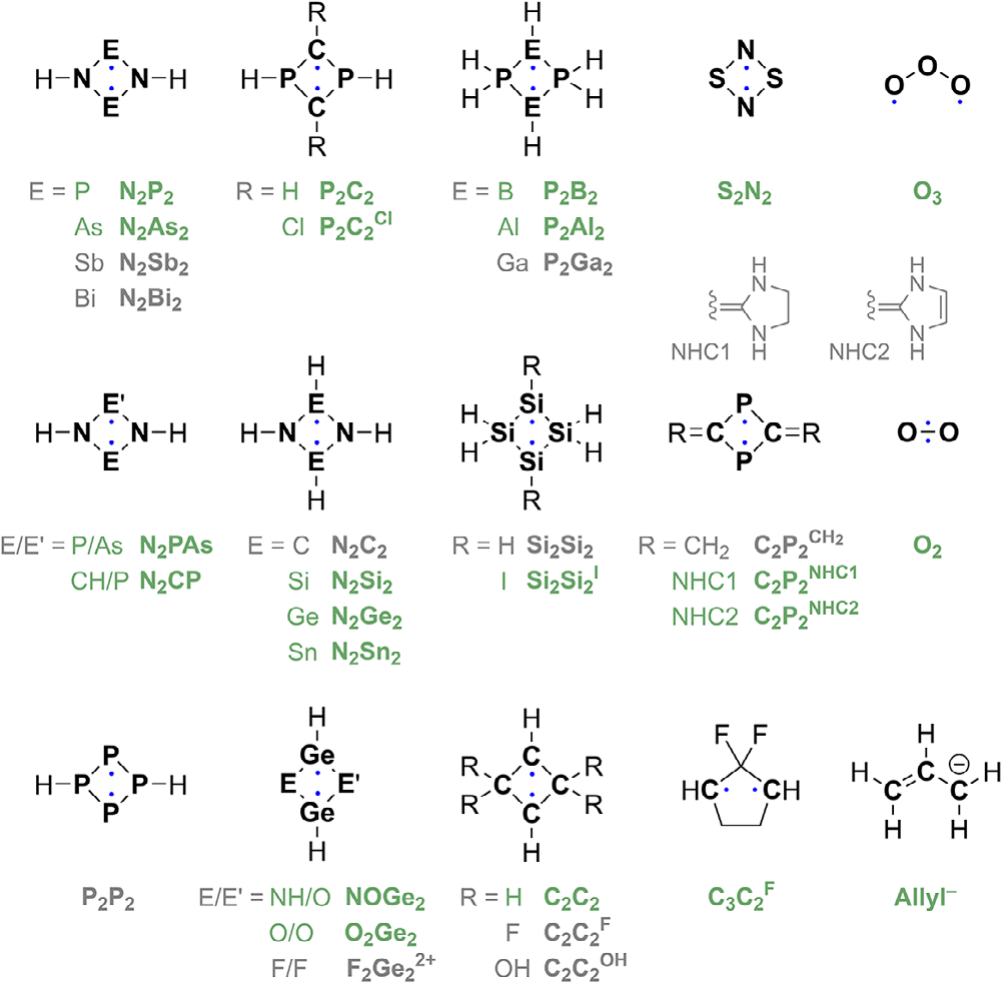
Overview of the structures that were investigated in our study. The structural motifs highlighted in green have been directly observed in experiments (i.e., at least by in‐situ spectroscopy), albeit typically with sterically demanding substituents instead of H. The singlet and triplet states of the model systems were optimized using DFT and CCSD(T) (with the notable exception of the C_2_P_2_^R^ derivatives, which were only optimized using DFT; for further details, please see the Supporting Information).

As model systems for our investigation we chose, somewhat arbitrarily, a selection of literature known [39, 40, 41, 42, 43, 44, 45, 46, 47, 48, 49, 50, 51, 52, 53, 54, 55, 56, 57, 58, 59] as well as hypothetical four‐membered ring systems that are formally isolobal to prototypical disulfur dinitride S_2_N_2_ [39, 40, 51], giving a total of 28 molecules (Figure 3). Organic substituents of synthetically known derivatives such as [P(μ‐NTer)]_2_ [41] (Ter = 2,6‐dimesitylphenyl) or [ClC(μ‐PMes*)]_2_ [53] (Mes* = 2,4,6‐tri‐*tert*‐butylphenyl) were deliberately replaced by H so as to exclude sterical or hyperconjugative effects from our calculations. Moreover, reducing the size of the model molecules was, of course, required to run MRCI+Q calculations. Thus, the biradical descriptors computed for the model systems do not necessarily reflect the biradical character of the synthetically accessible counterparts, depending on the electronic influence of the actual substituents. Additionally, dioxygen, ozone, the allyl anion and a cyclopentanediyl derivative [60] were included in our study to demonstrate a broader applicability of our conclusions.

All structures were optimized using density functional theory (DFT, PBE‐D3/def2‐TZVP) [61, 62, 63, 64, 65, 66] and, where feasible, also using coupled‐cluster theory (CCSD(T)/def2‐TZVP) [28, 67, 68, 69, 70, 71]. An unrestricted approach was used where appropriate, cf. Supporting Information (SI, p. S2ff). Frequency analyses were run to characterize the stationary points on the potential energy surface (PES) as minima or transition states. The coupled‐cluster geometries were preferably used for multireference calculations. Different types of CASSCF [72, 73, 74, 75, 76] calculations were run: A minimal active space of two electrons in two orbitals was used for MRCI+Q [77, 78] as well as NEVPT2 [79, 80, 81] calculations. A larger active space including all formal π electrons was used for bonding analysis and orbital localization. All calculations used a triple‐zeta basis set (def2‐TZVP or aug‐cc‐pVTZ) [82]. Computations were carried out using Gaussian [83, 84] and ORCA [85, 86, 87, 88]; for more details please refer to the Supporting Information.

## 3 Results and Discussion

### 3.1 Structure Optimization

To begin with, we needed reliable structures that could be used in high‐level ab‐initio calculations to investigate the electronic situation and biradical character of our model systems. Naturally, we were also interested in the accuracy of DFT optimized structures, since at the end of the day, others should be able to use our strategy to also investigate the electronic structure of “real” molecules with large substituents (even though this is not the scope of the present work). Since all molecules possess biradical character to a varying degree, we chose the pure functional PBE, since on the one hand, pure functionals are more robust with respect to nondynamical correlation (i.e., biradical character) [8, 9, 28, 89], and on the other hand, the PBE exchange‐correlation functional has proven reliable in our hands on many occasions [24, 90, 91].

When using currently available Kohn‐Sham DFT methods such as PBE to describe singlet biradicaloids, one issue that needs to be tracked is symmetry breaking of the Kohn‐Sham wavefunction at a moderate biradical character [28, 92]. For PBE, our data show that this happens at around 50% biradical character (Tables S1,S5), but especially for hybrid DFT methods, this value might be significantly lower [9]. As a rule of thumb, hybrid DFT methods become more prone to symmetry breaking with an increasing amount of Hartree‐Fock (HF) exchange [9, 10, 28]. As the broken symmetry solution corresponds to a mixture of the true (multireference) singlet state and the triplet state, care must be taken when working with those results (cf. Supporting Information, p. S23) [28].

To evaluate the quality of the DFT results, they were benchmarked against a set of CCSD(T) optimized structures (cf. Figure 3 and Tables S2, S6, S7). While CCSD(T) is, of course, also a single reference method, it is in our opinion still the best compromise between accuracy and computational effort (as also evidenced, for example, by comparison of the ST gaps with those from MRCI calculations, vide infra). Coupled‐cluster methods recover a significant amount of electron correlation, and it is known that CCSD(T) often gives reliable results even in the case of considerable multireference character [28, 93, 94, 95]. (Note that at the Full‐CI limit the reference wavefunction is of no concern; thus, the more electron correlation is included, the more robust the results will be with respect to multireference character.) [28].

The general agreement between the DFT and CCSD(T) optimized structures is very good (cf. Supporting Information p. S24ff, Tables S6, S7). If we consider all heavy‐atom distances (including the transannular distances), the mean signed error (MSE) amounts to 0.0078 Å (i.e., less than 1 pm), while the mean absolute error (MAE) is 0.014 Å (1.4 pm). The MSE in bond angles (−0.15°) and dihedral angles (−0.44°) are also small. Yet, it is noteworthy that DFT fails to predict a planar structure (*D*_2_ *_h_*) for **N_2_As_2_** and **N_2_Sb_2_** but introduces a small nonplanarity at the N atoms, resulting in a *C*_2_ *_h_*‐symmetric structure. These small structural differences do not affect the electronic structure significantly, though.

Finally, a few intricacies for those with a particular interest in four‐membered cyclic biradicaloids: All species were optimized with a *planar* four‐membered ring system. Thus, the model systems with a transannular π‐bond between the radical centers (vide infra), that is **P_2_B_2_**, **P_2_Al_2_**, **P_2_Ga_2_**, and **Si_2_Si_2_**, were optimized as saddle points on the PES, which connect two lower‐energy, [1.1.0]bicyclic minima incorporating a transannular σ‐bond [48, 54, 59, 96] (which we were not interested in). In the corresponding experimentally accessible species [48, 54, 59], large sterically demanding substituents introduce sufficient Pauli repulsion that the planar ring systems become minima on the PES, so the planar model systems describe these species adequately.

### 3.2 Singlet‐Triplet Gaps

As the coupling between the two radical electrons is one of the defining parameters of a biradical(oid), any computational method used to describe these species should be able to compute the ST energy gap (ST gap) within reasonable accuracy. In particular, the vertical ST gap is of interest, since it is directly related to the electronic exchange coupling constant *J* (vide supra).

We computed vertical ST gaps using PBE‐D3 and CCSD(T), as well as the multireference ab‐initio methods NEVPT2 and MRCI+Q (for details, see Supporting Information p. S10ff). DFT calculations used the DFT optimized structures, while ab‐initio methods employed the CCSD(T) optimized structures (except for **C_2_P_2_^R^**, where DFT structures were used as well). The MRCI results, representing the highest‐accuracy reference available, served as the benchmark against which all other computational methods were evaluated.

In general, all methods agree reasonably well (Tables 1 and S5). The best agreement is found between CCSD(T) and MRCI. On average, the coupled‐cluster results are very close to the MRCI values (mean signed error, MSE: −1.1 kJ/mol) with a low dispersion of the coupled‐cluster ST gaps (mean absolute error, MAE: 6.6 kJ/mol). Notice that the largest deviations from the MRCI results are found for the triplet ground state species **C_2_C_2_** and **O_2_**, which is not surprising considering that their singlet states have nearly 100% biradical character. NEVPT2 seems to very slightly overestimate the stability of the singlet state on average (MSE: −5.8 kJ/mol) and shows a slightly wider dispersion (MAE: 8.4 kJ/mol) than CCSD(T). Even the DFT results are reasonably accurate; the MSE of −5.7 kJ/mol is similar to that of NEVPT2, but the dispersion is somewhat larger (MAE: 12.3 kJ/mol). This indicates that PBE‐D3 (and likely other pure functionals) can be a good choice for molecules with larger substituents, which cannot be easily treated using high‐level ab‐initio methods.

**TABLE 1:** Vertical ST energy gaps Δ*E*_ST_^v^ as computed by MRCI and deviation Δ(Δ*E*_ST_^v^) of the other methods from the MRCI results. All energies in kJ/mol. PG = point group.

	PG	Δ*E*_ST_^v^	Δ(Δ*E*_ST_^v^)		
		MRCI	CCSD(T)	NEVPT2	PBE‐D3
**N_2_P_2_**	*D*_2_ *_h_*	−144.6	−4.1	−6.9	−13.3
**N_2_As_2_**	*D*_2_ *_h_*	−114.0	−8.8	−12.2	−15.3
**N_2_Sb_2_**	*D*_2_ *_h_*	−70.2	−9.9	−11.5	−13.8
**N_2_Bi_2_**	*C*_2_ *_h_*	−58.9	−5.2	−12.6	−11.3
**P_2_C_2_^Cl^**	*C_i_*	−71.7	−12.1	−9.4	−15.0
**P_2_C_2_**	*C_i_*	−124.1	−10.1	−8.8	−12.7
**P_2_B_2_**	*D*_2_ *_h_*	−119.0	+5.3	−3.7	+15.0
**P_2_Al_2_**	*D*_2_ *_h_*	−134.9	−2.0	−8.3	+7.7
**P_2_Ga_2_**	*C*_2_ *_h_*	−91.0	−2.1	−7.2	−0.2
**S_2_N_2_**	*D*_2_ *_h_*	−288.6	−6.7	−21.5	−11.8
**O_3_**	*C*_2_ *_v_*	−160.0	+4.4	−26.5	−24.4
**N_2_C_2_**	*C_i_*	−226.2	−2.0	−4.5	−12.2
**N_2_Si_2_**	*C*_2_ *_h_*	−67.6	+0.5	+1.7	−5.0
**N_2_Si_2_**	*D*_2_ *_h_*	−69.7	−4.3	0.0	+2.3
**N_2_Ge_2_**	*C_i_*	−76.2	−2.8	−3.8	−20.5
**N_2_Sn_2_**	*C_i_*	−63.3	−4.2	−6.7	−18.5
**NOGe_2_**	*C*_2_	−60.4	−2.5	−3.5	−20.1
**O_2_Ge_2_**	*C*_2_ *_h_*	−42.5	−0.7	−1.6	−16.7
**F_2_Ge_2_^2+^**	*C*_2_ *_h_*	−7.3	−1.0	+0.2	−8.2
**Si_2_Si_2_^I^**	*D*_2_ *_h_*	−118.7	−3.5	−11.7	+0.3
**Si_2_Si_2_**	*D*_2_ *_h_*	−84.7	+3.7	−5.0	+7.8
**P_2_P_2_**	*C*_2_ *_h_*	−132.8	−1.5	−2.1	−0.8
**P_2_P_2_**	*C_s_*	−146.2	+0.1	−3.5	―^[^ ^a^ ^]^
**N_2_PAs** ^[^ ^b^ ^]^	*C*_2_ *_v_*	−134.9	−7.6	−11.0	−14.8
**N_2_PAs** ^[^ ^c^ ^]^	*C*_2_ *_v_*	−192.7	+17.1	+23.8	+35.2
**N_2_CP** ^[^ ^b^ ^]^	*C*_2_	−245.6	−7.2	−15.0	+25.2
**N_2_CP** ^[^ ^d^ ^]^	*C*_2_	−199.1	−12.3	+3.6	+0.3
**C_2_P_2_^CH2^**	*D*_2_ *_h_*	+64.6	―	−7.9	−22.7
**O_2_**	*D*_∞_ *_h_*	+95.7	+30.0	+14.2	−21.7
**C_2_C_2_**	*C*_2_ *_h_*	+12.6	+18.1	+0.7	+0.7
**C_2_C_2_^F^**	*D*_2_ *_h_*	−115.7	+1.5	−7.7	+1.5
**C_2_C_2_^OH^**	*D*_2_	−82.3	+2.5	−6.2	−2.3
**C_3_C_2_^F^**	*C*_2_ *_v_*	−55.0	+6.6	−3.4	−15.9
**Allyl^−^**	*C*_2_ *_v_*	−213.3	−16.8	−20.9	+13.7

^[a]^ Not a stationary point on the PES at this level of theory.

^[b]^ 1,3‐diyl.

^[c]^ arsinidene.

^[d]^ phosphinidene.

We screened the literature for previous reports on ST gaps of the model systems we calculated, and those reports we could find generally agree quite well with our calculated data [7, 8, 17, 53, 54, 97, 98, 99, 100, 101, 102]. This is true for vertical (Δ*E*_ST_^v^) as well as adiabatic ST gaps (Δ*E*_ST_^a^, Table S5). Depending on the distortion of the geometry in the triplet state compared to the singlet state, the adiabatic energy differences may be quite similar (e.g. **N_2_Si_2_** CCSD(T): vertical −67.1 vs. adiabatic −66.0 kJ/mol) or much smaller than the vertical ST gap (e.g. **P_2_C_2_^Cl^** CCSD(T): −83.7 vs. −4.6 kJ/mol). The latter example is particularly noteworthy as it represents the smallest adiabatic ST gap of all biradical(oid)s in our study. The small adiabatic ST gaps of **P_2_C_2_^Cl^** and also **P_2_C_2_**, especially in comparison with other experimentally accessible biradicaloids, have previously been recognized in the literature and are traced back to the influence of the pyramidalization at the P and C atoms of the central ring system [7, 53, 97].

### 3.3 Biradical Character: Theoretical Considerations

With a set of reliable ST gaps, it is now time to turn our attention to one of the essential questions of this study: What does the ST gap tell us about biradical character?

From a reactivity standpoint, clearly the *adiabatic* ST gap is important, as it gives us an idea whether the triplet state may be thermally accessible. If so, reactions may occur both on the singlet and triplet surface [8], with consequences for the possible reaction outcomes. “Closed‐shell” (singlet) reactivity often involves cycloadditions, whereas “open‐shell” reactivity is frequently defined by stepwise (radical‐like) reaction mechanisms [8, 103].

As mentioned before, the *vertical* ST gap may be taken as a measure of the magnetic interaction between the electrons. Thus, one might expect that it could also serve as a direct measure of singlet biradical character. However, the situation is not that simple: In the two‐electrons‐in‐two‐orbitals (2e2o) picture, the vertical ST gap is computed by [10]

(14) $$\begin{array}{c} \Delta \;E_{\mathrm{ST}}^{v}=C+K_{\mathrm{ab}}-\frac{1}{2}\sqrt{\Delta E_{\mathrm{ab}}^{2}+4K_{\mathrm{ab}}^{2}}\; \end{array}$$

with

(15) $$\begin{array}{c} C=\frac{J_{\mathrm{aa}}+J_{\mathrm{bb}}}{2}\;-J_{\mathrm{ab}}\; \end{array}$$

where *J*_aa_, *J*_bb_, and *J*_ab_ are the one‐ and two‐site Coulomb integrals of the delocalized orbitals a and b, *K*_ab_ is the exchange integral between those orbitals, and Δ*E*_ab_ = (*H*_bb_—*H*_aa_) is the energy difference between the two configurations $|a^{2}\rangle$ and $|b^{2}\rangle$ (which may be understood as a measure of the HOMO‐LUMO gap).

On the other hand, the singlet biradical character in the 2e2o picture may be inferred from the CI coefficient *c*_2_ of the two‐determinantal wavefunction of the lowest singlet state $\Psi_{S_{-}}$ (cf. eqn. 3 and 7):

$$\beta =2c_{2}^{2}$$

The CI coefficients may alternatively be expressed in terms of the parameter *τ*,

(16) $$\;c_{1}=\cos\left(\tau \right)\;\;$$

(17) $$\;c_{2}=\sin\left(\tau \right)\;\;$$

which is linked to the previously introduced energy difference Δ*E*_ab_ and the exchange integral *K*_ab_ [10]

(18) $$\tau =-\frac{1}{2}\arctan\left(\frac{2K_{\mathrm{ab}}}{\Delta E_{\mathrm{ab}}}\right)\;$$

Substituting equations 17 and 18 into eqn. 7, we get

$$\beta =2\sin^{2}\left[-\frac{1}{2}\arctan\left(\frac{2K_{ab}}{\Delta E_{\mathrm{ab}}}\right)\right]$$

which may be further simplified using sin(−*x*)  =  −sin(*x*) and the trigonometric identity 2 sin^2^(*x*)  =  1−cos(2*x*)

$$\begin{array}{c} \beta =1-\cos\left[\arctan\left(\frac{2K_{ab}}{\Delta E_{\mathrm{ab}}}\right)\right] \end{array}$$

Finally, using cos[arctan(x)]  =  1/√(1+x2) and expanding the fraction with Δ*E*_ab_, we arrive at

(19) $$\begin{array}{c} \beta =1-\frac{\Delta E_{\mathrm{ab}}}{\sqrt{\Delta E_{\mathrm{ab}}^{2}+4K_{\mathrm{ab}}^{2}}}\; \end{array}$$

Thus, the biradical character solely depends on the energy difference Δ*E*_ab_ and the exchange integral *K*_ab_, whereas the ST gap additionally depends on the one‐ and two‐site Coulomb integrals *J*_aa_, *J*_bb_, and *J*_ab_. *In consequence, even if we just consider the basic 2e2o model, there is no “simple” mathematical relationship between ST gap and biradical character*. When combining equations 14 and 19 and eliminating Δ*E*_ab_ (cf. Supporting Information p. S69), we find that the relationship between Δ*E*_ST_^v^ and *β* depends on the magnitude of the Coulomb and exchange integrals, which naturally differ between different biradical(oid)s:

(20) $$\begin{array}{c} \Delta \;E_{\mathrm{ST}}^{v}=C+K_{\mathrm{ab}}\left(1-\frac{1}{\sqrt{\beta \left(2-\beta \right)}}\right)\; \end{array}$$

**FIGURE 4:**
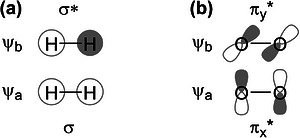
Delocalized orbitals a and b for (a) dihydrogen and (b) dioxygen.

To interpret the meaning of equation 20, let us first consider the term *C*  =  (*J*_aa_+*J*_bb_)/2 – *J*_ab_ (eqn. 15): In the case of disjoint biradical(oid)s [14, 17, 104] (i.e., those where the radical electrons can be localized on different atoms or in different regions of the molecule), the *delocalized* molecular orbitals a and b are typically bonding and antibonding (in‐phase and out‐of‐phase) combinations of the same atomic orbitals [10]. The simplest example is stretched H_2_, where the delocalized orbitals (*σ*, σ*) are combinations of the two 1s orbitals at the H atoms (Figure 4a). Since the electrons occupy roughly the same region of space in both delocalized orbitals, the average of the two one‐site Coulomb integrals (*J*_aa_+*J*_bb_)/2 is of similar magnitude as the two‐site Coulomb integral *J*_ab_. Overall, the term *C*  =  (*J*_aa_+*J*_bb_)/2 – *J*_ab_ will become small or close to 0.

Conversely, in the case of nondisjoint biradical(oid)s [14, 17, 104] (i.e., those where the radical electrons share a significant region of space), the delocalized orbitals are *not* linear combinations of the same atomic orbitals [10]. A simple example is dioxygen with its *π_x_* * and *π_y_* * orbitals, which are linear combinations of the 2p_x_ and 2p_y_ orbitals, respectively (Figure 4b). Here, the average of the two one‐site Coulomb integrals (*J*_aa_+*J*_bb_)/2 is significantly larger than the two‐site Coulomb integral *J*_ab_, as the two delocalized orbitals have different spatial orientations (i.e., interelectronic repulsion is larger if both electrons are in the same orbital than if they are in different orbitals). Thus, the term *C*  =  (*J*_aa_+*J*_bb_)/2 – *J*_ab_ will be large. This is in agreement with the well‐established fact that nondisjoint biradicals usually have large positive ST gaps [8, 15].

The exchange integral *K*_ab_ modulates how strongly the biradical character *β* affects the ST gap Δ*E*_ST_^v^. *K*_ab_ will be large in those molecules where the two orbitals a and b are located in the same region of space (as, for example, in stretched H_2_), whereas it will become small if the two orbitals are in different regions of the molecule (e.g., in case of a metal‐centered HOMO and ligand‐centered LUMO). This is in line with our previous analysis that a small HOMO‐LUMO gap alone does not reflect biradical character; in fact, it only does if the exchange integral is significantly large [10].

**FIGURE 5:**
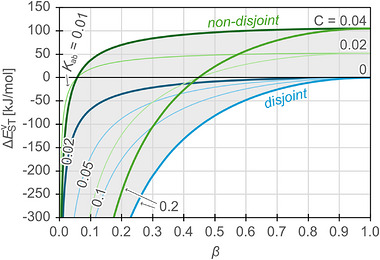
Plot of the relationship between Δ*E*_ST_^v^ and *β* according to eqn. 20. The values of *K*_ab_ are indicated on the left, the values of *C* are indicated on the right (in atomic units). Clearly, there is no unique relation between ST gap and biradical character; the majority of biradical(oid)s should be found somewhere within or in the vicinity of the shaded region. The “boundaries” are somewhat arbitrarily chosen; larger values of *K*_ab_ and *C* are, of course, possible.

With the above considerations, Δ*E*_ST_^v^ versus *β* can be plotted for different combinations of the parameters *C* and *K_ab_* (Figure 5). Note that typically *C* ≳ 2*K*_ab_ [8, 10], which limits the possible combinations of these parameters.

For *C*  = 0, isolines with *K*_ab_  = 0.02, 0.05, 0.1, and 0.2 were plotted, which accounts for most disjoint biradical(oid)s (blue lines in Figure 5). Smaller values of *K*_ab_ are expected only in the case of spatially separated frontier orbitals, resulting in little to no biradical character (vide supra).

To represent nondisjoint biradicals, we chose *C*  = 0.04, which results in a ST gap of approx. +100 kJ/mol at *β*  =  1, which roughly corresponds to the parameters found for dioxygen (vide infra). Two isolines at *K*_ab_  = 0.02 and 0.2 (thick green lines) indicate weak and strong exchange, analogously to the disjoint case. Lastly, *C*  = 0.02 was chosen as an intermediate value, with *K*_ab_  = 0.01 and 0.1 as exemplary values for the exchange interaction (thin green lines).

Please note that the isolines do not represent actual chemical processes that influence the biradical character of a system (such as dissociation of H_2_ or C = C bond rotation of ethylene, cf. Supporting Information p. S64ff). In those instances, both parameters *K*_ab_ and *C* will change simultaneously during the process. Nonetheless, Figure 5 nicely demonstrates that *the relationship between two very important descriptors of biradical character, ΔE_ST_^v^ and β, is diffuse;* and one quantity cannot be derived from the other (at least not without further information).

### 3.4 Biradical Character: Computations

To compare the theoretical deliberations derived from the simple 2e2o model with high‐level calculations, we set out to compute the singlet biradical character of the molecules listed in Figure 3. To obtain reliable data and also to explore the influence of dynamical correlation, we evaluated the LUNO occupancy [32] at different levels of theory: a simple CASSCF(2,2) wavefunction was used to exclude dynamical correlation, while NEVPT2 and MRCI natural orbital occupancies were utilized to include dynamical correlation (Table 2). Moreover, we ran CASSCF calculations encompassing additional orbitals in the valence region to verify that a minimal (2,2) active space was sufficient in all cases (cf. Supporting Information p. S11‐S23).

**TABLE 2:** LUNO occupancies computed at different levels of theory. Note that for CASSCF(2,2), *n*_LUNO_ = *β*. A CASSCF(2,2) reference was used in all cases. Additionally, the vertical ST gaps are indicated (MRCI results unless noted otherwise, cf. Table 1). PG = point group.

		*n*_LUNO_			
	PG	CAS	NEVPT2	MRCI	Δ*E*_ST_^v^ [kJ/mol]
**N_2_P_2_**	*D*_2_ *_h_*	0.24	0.27	0.20	−144.6
**N_2_As_2_**	*D*_2_ *_h_*	0.29	0.32	0.23	−114.0
**N_2_Sb_2_**	*D*_2_ *_h_*	0.38	0.40	0.31	−70.2
**N_2_Bi_2_**	*C*_2_ *_h_*	0.43	0.45	0.37	−58.9
**P_2_C_2_^Cl^**	*C_i_*	0.31	0.33	0.26	−71.7
**P_2_C_2_**	*C_i_*	0.19	0.23	0.16	−124.1
**P_2_B_2_**	*D*_2_ *_h_*	0.19	0.21	0.16	−119.0
**P_2_Al_2_**	*D*_2_ *_h_*	0.10	0.12	0.09	−134.9
**P_2_Ga_2_**	*C*_2_ *_h_*	0.19	0.21	0.17	−91.0
**S_2_N_2_**	*D*_2_ *_h_*	0.17	0.21	0.14	−288.6
**O_3_**	*C*_2_ *_v_*	0.42	0.45	0.31	−160.0
**N_2_C_2_**	*C_i_*	0.21	0.23	0.17	−226.2
**N_2_Si_2_**	*C*_2_ *_h_*	0.48	0.49	0.41	−67.6
**N_2_Si_2_**	*D*_2_ *_h_*	0.37	0.39	0.31	−69.7
**N_2_Ge_2_**	*C_i_*	0.42	0.44	0.36	−76.2
**N_2_Sn_2_**	*C_i_*	0.45	0.46	0.38	−63.3
**NOGe_2_**	*C*_2_	0.48	0.49	0.41	−60.4
**O_2_Ge_2_**	*C*_2_ *_h_*	0.56	0.57	0.49	−42.5
**F_2_Ge_2_^2+^**	*C*_2_ *_h_*	0.81	0.81	0.77	−7.3
**Si_2_Si_2_^I^**	*D*_2_ *_h_*	0.17	0.20	0.15	−118.7
**Si_2_Si_2_**	*D*_2_ *_h_*	0.25	0.27	0.22	−84.7
**P_2_P_2_**	*C*_2_ *_h_*	0.18	0.22	0.16	−132.8
**P_2_P_2_**	*C_s_*	0.16	0.21	0.15	−146.2
**N_2_PAs** ^a^	*C*_2_ *_v_*	0.24	0.27	0.19	−134.9
**N_2_PAs** ^b^	*C*_2_ *_v_*	0.00	0.03	0.02	−192.7
**N_2_CP** ^a^	*C*_2_	0.09	0.13	0.09	−245.6
**N_2_CP** ^c^	*C*_2_	0.01	0.06	0.04	−199.1
**C_2_P_2_^CH2^**	*D*_2_ *_h_*	0.99	0.98	0.96	+64.6
**C_2_P_2_^NHC1^**	*C*_2_ *_h_*	0.27	0.30	―	−64.6^d^
**C_2_P_2_^NHC2^**	*D*_2_ *_h_*	0.22	0.25	―	−99.2^d^
**O_2_**	*D*_∞_ *_h_*	1.00	1.00^e^	1.00^e^	+95.7
**C_2_C_2_**	*C*_2_ *_h_*	0.92	0.91	0.92	+12.6
**C_2_C_2_^F^**	*D*_2_ *_h_*	0.33	0.34	0.28	−115.7
**C_2_C_2_^OH^**	*D*_2_	0.41	0.41	0.35	−82.3
**C_3_C_2_^F^**	*C*_2_ *_v_*	0.51	0.52	0.45	−55.0
**Allyl^−^**	*C*_2_ *_v_*	0.05	0.08	0.06	−213.3

^a^ 1,3‐diyl.

^b^ arsinidene.

^c^ phosphinidene.

^d^ NEVPT2 results;.

^e^ corrected values (the computed LUNO occupancy is slightly larger than 1 due to numerical noise, cf. Table S5).

Most importantly, *state‐specific* reference wavefunctions were used for the investigation of singlet biradical character. This is important insofar as the state specific natural orbitals allow for the most compact representation of the singlet wavefunction [10, 11, 28], as they correctly diagonalize the singlet density matrix (which is what we are interested in). *State‐averaged* natural orbitals, on the other hand, though useful for the determination of vertical energy differences between the biradical states, would be ill‐suited to analyze the singlet wavefunction, as they diagonalize the state‐averaged density matrix involving also the other electronic states, so they might not fully capture the nondynamical correlation in the singlet state. While the relevant orbitals might superficially look similar (as they should), the orbital coefficients are typically different, resulting also in different CI coefficients and in turn different (or even wrong) values of biradical character. For example, state‐averaged CASSCF incorrectly predicts only 16% biradical character for the singlet ground state ($\Psi_{S_{-}}$) of **P_2_C_2_^Cl^**, which is only about half of the value computed by state‐specific CASSCF (31%).

**FIGURE 6:**
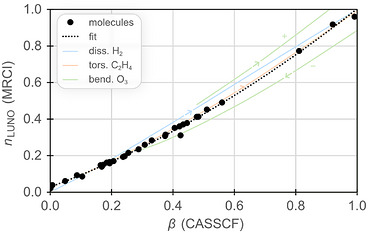
LUNO occupancy *n*_LUNO_ from MRCI calculations plotted against the LUNO occupancy (= *β*) from CASSCF(2,2). The solid lines indicate the following processes: dissociation of H_2_ (light blue), torsion of ethylene around the C═C double bond (light red) and bending of O_3_ starting from a nonequilibrium structure with ∡(OOO)  =  60° (light green; upper branch (+) is bonding, lower branch (−) is antibonding, cf. Supporting Information p. S64ff). The arrows indicate the direction of increasing H‐H distance, HCCH dihedral angle, and OOO bond angle, respectively.

**FIGURE 7:**
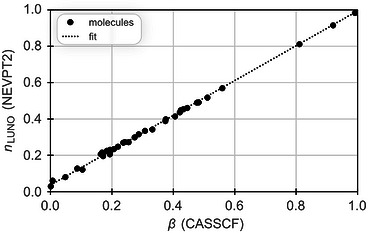
LUNO occupancy *n*_LUNO_ from NEVPT2 calculations (relaxed density) plotted against the LUNO occupancy *β* from CASSCF(2,2).

For the molecules investigated in our study, the calculated values of biradical character using the three different electronic structure methods correlate well (Figures 6, 7). The NEVPT2 and CASSCF results are quite similar. At the MRCI level of theory, the inclusion of dynamical correlation leads to noticeable deviations. For small values of biradical character (< 10%), the MRCI values are slightly higher than the CASSCF results, whereas for larger values of biradical character (>10%), the inclusion of dynamical correlation somewhat lowers the values in comparison to the CASSCF results. Please note, however, that these observations cannot be generalized to other compound classes; especially in molecules with extended π‐bonding systems, inclusion of dynamical correlation may also increase the biradical character [90, 91].

To understand and interpret the data, three model processes that vary the biradical character were included in Figure 6, namely the dissociation of H_2_, torsion of ethylene around the C═C double bond and bending of O_3_ (cf. Supporting Information p. S64ff). The former two align well with the data for the four‐membered ring systems. In case of the latter, the biradical character differs more significantly between CASSCF and MRCI calculations, which we attribute to the circumstance that the interaction between the two radical electrons changes from bonding to antibonding during the process (with a cusp in biradical character at 100%, vide infra). Since this is connected to an interchange of the frontier orbitals (Table S9), clearly this process is very sensitive toward the orbital energies/occupancies, and the inclusion of dynamical correlation therefore has a more pronounced effect on the biradical character than in most cases.

Apart from this “extreme” example, it is worthy to note that LUNO occupancy (as measure of singlet biradical character) computed using the three different methods outlined above usually only varies within a few percentage points. We therefore propose that the values should be considered accurate to about ±2 or ±3 units. That is, for example, 22 and 25% singlet biradical character should *not* be regarded as significantly different; however, 20 and 30% should. Of course, it is always preferable to compare only those values which were derived using the same computational method, but for a rough orientation, even data from different levels of theory can work. In particular, for large molecules which cannot be treated by high‐level MRCI computations, CASSCF(2,2) can be a reasonable choice at a low computational cost. Note, however, that this method solely describes nondynamical correlation, whereas effects from dynamical correlation are not included. They can be estimated, however, by increasing the active space, for example by including all π electrons into the active space in the case of (nearly) planar ring systems. (With regard to the four‐membered ring systems in our study, this would correspond to a (6,4) active space, cf. Supporting Information p. S21 and S48ff.)

**FIGURE 8:**
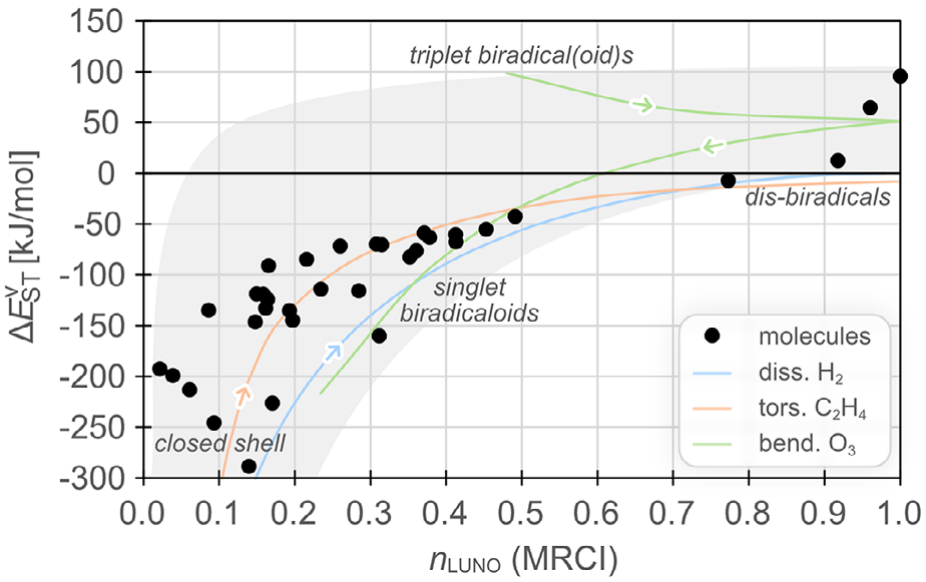
ST gap versus singlet biradical character *n*_LUNO_ for the model systems in Figure 3. The shaded region corresponds to the predicted range from the simple 2e2o model (cf. Figure 5). Additionally, the dissociation of H_2_, torsion of ethylene, and bending of O_3_ are shown (cf. Supporting Information p. S64ff). The arrows indicate the direction of increasing H‐H distance, HCCH dihedral angle, and OOO bond angle, respectively.

For the remainder of this article, we will continue to use the LUNO occupancy from the MRCI calculations as the indicator of singlet biradical character. (For some further indices of biradical character, please refer to the Supporting Information, p. S70f.) Following our initial intent, we would like to further discuss the relationship between ST gap and singlet biradical character, and how the computed data compare to the predicted range from the simple 2e2o model (Figure 8).

Clearly, the calculated results are found within the predicted range. Not the whole area is filled, which is due to the fact that most of the model molecules we selected belong to similar classes of compounds. Still, even though there are a lot of structural similarities—and this is one of the key implications of our work—we do find quite a large distribution of possible combinations of singlet biradical character and ST gap. Hence, *the ST gap alone is not a good indicator of singlet biradical character*.

Let us examine one of the more familiar singlet biradicaloids: ozone. Despite its considerable singlet biradical character of 31%, its vertical ST gap amounts to a rather substantial value of −160.0 kJ/mol. The latter is in good agreement with previous results [100] and in alignment with ozone's frequent role as 1,3‐dipolarophile (closed‐shell reactivity) [100]. We do find, however, many different values for ozone's biradical character in the literature [100, 105, 106, 107], which is mainly due to the previously mentioned fact that different indices of singlet biradical character were used, so the results cannot be directly compared.

This leads to a frequent misunderstanding: Some sources cite a limit of −1 eV (ca. −100 kJ/mol) for the ST gap of singlet biradicaloids [108, 109]. This is clearly too conservative, and our data suggests that a ST gap of −100 kJ/mol can be associated with values of singlet biradical character from approx. 0.1 up to 0.4 (or 10–40%). Thus, *both ST gap and singlet biradical character must be considered* to classify whether or not a molecule has biradical character.

In this regard, it is also noteworthy that a singlet biradical character up to 10% may be considered dynamical correlation [32, 35, 110, 111, 112]. A molecule with a ST gap of −100 kJ/mol and 10% singlet biradical character would therefore surely be considered a “closed‐shell” species. If, on the other hand, the molecule had 40% singlet biradical character instead of 10%, it would be classified as a singlet biradicaloid. In both cases, the ST gap of −100 kJ/mol is likely too large to expect reactions on the triplet PES, but due to the significant singlet biradical character in the second case, stepwise radical‐like reactions may also be initiated via the singlet PES [103]. Moreover, a substantial singlet biradical character may also indicate high reactivity in concerted reactions like cycloadditions [8, 19].

In Figure 8, different regions are labeled according to the different types of biradical(oid)s, so as to incorporate both ST gap and singlet biradical character in their definition: Molecules with a significant negative ST gap and low singlet biradical character are closed shells, those with intermediate values are (open‐shell) singlet biradicaloids, and those with a ST gap approaching zero and a singlet biradical character of (nearly) 100% are dis‐biradicals. Species with a positive ST gap are triplet biradical(oid)s. The remaining (unlabeled) region of low singlet biradical character and a negative ST gap close to zero relates to metal complexes with low lying metal‐to‐ligand‐charge‐transfer (MLCT) triplet states, often connected with phosphorescence phenomena [113]. We deliberately do not introduce boundaries between the different categories, as the transitions between them are continuous.

Let us look at some additional examples: The very first room‐temperature stable biradicaloid, [ClC(μ‐PMes*)]_2_ [53], is described by the model **P_2_C_2_^Cl^** (Δ*E*_ST_^v^  = −71.7 kJ/mol, *n*_LUNO_  = 26%), which has significant singlet biradical character and also a rather small ST gap. On the other hand, **P_2_B_2_** (Δ*E*_ST_^v^  = −119.0 kJ/mol, *n*_LUNO_  = 16%), which is a model of the second isolated biradicaloid [*t*BuB(*μ*‐P*i*Pr_2_)]_2_ [54]_,_ has only low biradical character, in agreement with earlier reports [13]. One of the most thoroughly investigated systems, **N_2_P_2_** [114] (−144.6 kJ/mol, 20%), has an even more substantial ST gap, but its singlet biradical character is in between the other two. In accord with above considerations, **N_2_P_2_** derivatives are particularly reactive in cycloaddition reactions [9, 10]. **NOGe_2_** (−60.4 kJ/mol, 41%) and **N_2_Si_2_** (−67.6 kJ/mol, 41%) have, along with rather small ST gaps, the highest singlet biradical character out of those model systems whose structural motifs are also part of a structurally characterized molecule [42, 58]. (Interestingly, the singlet biradical character was not quantified in those instances.) Furthermore, it is evident in the series **N_2_Ge_2_** (36%), **NOGe_2_** (41%), **O_2_Ge_2_** (49%), and **F_2_Ge_2_^2+^** (77%) that the singlet biradical character increases with increasing electronegativity of the bridging atoms (N, O, F), along with decreasing magnitude of the ST gap (−76.2, −60.4, −42.5, −7.3 kJ/mol).

In summary, the calculated ST gaps and values of singlet biradical character are consistent with the expectations from the 2e2o model (shaded area in Figure 8). Where available, literature data also mostly agree with our analysis (if we factor out that different indices of biradical character were used which may result in numerical differences). However, one apparent discrepancy remains: Our results place **S_2_N_2_** (Δ*E*_ST_^v^  = −288.6 kJ/mol, *n*_LUNO_  = 14%), often cited as a prototypical singlet biradicaloid [106, 108, 115, 116], at the “closed‐shell” end of our classification of biradical(oid)s with only marginal biradical character. In fact, the discussion about the electronic structure and biradical character of **S_2_N_2_** has produced a plethora of publications (a selection may be found in the references) [39, 51, 102, 105, 106, 108, 115, 116], but to the best of our knowledge, the different views on its biradical character could not be reconciliated to date.

### 3.5 Orbital Localization

**FIGURE 9:**
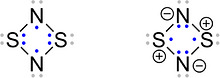
“Biradical” structures of S_2_N_2_. The structure on the left is the leading resonance structure, while only a low weight is assigned to the structure on the right. The π electrons are highlighted in blue.

Part of the confusion arises from an apparent discrepancy between the weight of “biradical” structures in the Lewis resonance scheme (i.e. from an interpretation of VB‐type wavefunctions in the *localized orbital basis*) and the computed biradical character from natural orbitals (i.e. using *delocalized orbitals*). The combined weight of the “biradical” structures of **S_2_N_2_** (Figure 9) is usually placed somewhere in the 50% region, while the computed singlet biradical character is typically low [105, 106], in agreement with our own results.

In fact, this disparity between the singlet biradical character and the weight of “biradical” structures in the VB picture has also been recognized for other species such as **O_3_** [8, 106] and **N_2_P_2_** [117]. It was therefore imperative to investigate this phenomenon further by analyzing the wavefunctions of all model systems in terms of Lewis‐type valence bond structures. Since we were primarily interested in the distribution of the π electrons, this was achieved by computing CASSCF wavefunctions including all formal π orbitals (i.e., typically a (6,4) active space), which were then localized [118, 119] to obtain a VB‐type description of the wavefunction (cf. Table S4 and Scheme S1). To verify that the (6,4) active space would not only be sufficient for the planar molecules, but also for those ring systems with substituents that are bent out of the central ring plane (which therefore include some mixing between the σ and π bonding systems), we also computed CASSCF(22,12) wavefunctions for two examples (**N_2_C_2_** and **N_2_Si_2_**), which encompass the full valence region (cf. Supporting Information p. S60). While the results are, of course, not identical, they are still sufficiently similar (within ∼10%). Since deviations of this order of magnitude would be expected anyway between different choices of localized orbitals and/or different partitioning schemes [8, 10, 105, 106], the simple (6,4) active space suffices for our purposes.

**FIGURE 10:**
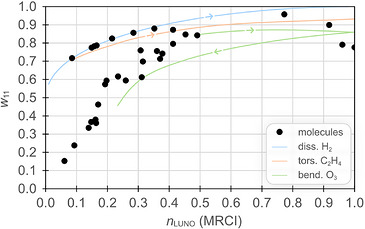
Weight *w*_11_ of the “biradical/covalent” resonance structure (formally A· ·B) versus singlet biradical character *n*_LUNO_. The dissociation of H_2_, torsion of ethylene, and bending of O_3_ are included as model processes (cf. Supporting Information p. S64ff). The arrows indicate the direction of increasing H─H distance, HCCH dihedral angle, and OOO bond angle, respectively.

Figure 10 shows the relationship between the weight *w*_11_ of the leading “biradical” resonance (i.e., the representation analogous to the leading resonance structure of S_2_N_2_ with 1 electron at each of the radical centers; see also Figure 3) and the singlet biradical character (*n*_LUNO_). It becomes immediately clear that, apart from a few exceptions of molecules with low to negligible singlet biradical character (< 20%), the “biradical” resonance has more than 50% weight (!) in the resonance scheme in most instances. In particular, the dissociation of H_2_, starting in the closed‐shell region at its equilibrium distance, begins at about *w*_11_  = 60% which then increases rapidly (blue curve). Of course, H_2_ at its equilibrium distance is a classical “closed‐shell” molecule, and since the two H atoms are in close proximity, we would not write this situation as H· ·H but rather as H─H, but it is important to note that *both are actually the same “covalent” contribution to the singlet wavefunction* represented by the configurations $|1\bar{2}\rangle$ and $|\bar{1}2\rangle$ (cf. eqn. 9). More precisely, the notation H·–·H is sometimes used in VB theory to refer to this “covalent” part of the wavefunction and to distinguish it from the “actual” atomic bond (commonly written as H─H), which contains *covalent and ionic* contributions, that is, H·–·H, ^−^H: H^+^ and ^+^H :H^−^ [120].

When the singlet biradical character of the pair of hydrogen atoms increases as we approach the dissociation limit, the wavefunction is exclusively described by the “biradical/covalent” part (i.e., it has 100% biradical character) [9, 10]. Clearly, we would now be inclined to choose the representation H· ·H over H─H, in alignment with the notion of two separate H radicals. This seemingly somewhat arbitrary choice of two different Lewis representations for the same configurations in the VB picture is unsatisfactory, and we will deal with this issue shortly.

Beforehand, we want to provide some more context on Figure 10. The dissociation of H_2_ (light blue line) may be regarded as the “localized limit”: in H_2_ at its equilibrium distance, the electrons form a localized 2‐electron‐2‐center (2e2c) bond. At the dissociation limit, one electron is localized at each of the two H radicals. Molecules with lower values of *w*_11_ (at constant *n*_LUNO_) therefore indicate more delocalized bonding situations (e.g., delocalization of π‐electrons). This makes sense, as they need additional Lewis resonance structures to describe the electronic situation, therefore lowering the weight of the leading resonance structure. For example, in case of **S_2_N_2_**, the leading resonance has a weight of 33%, indicating a more delocalized electronic structure in agreement with previous reports [106, 108].

### 3.6 Bond Order

Both quantities *w*_11_ and *n*_LUNO_ are clearly complementary and must be discussed together to attain a complete understanding of the electronic structure. Still, how can we reconciliate a weight of *w*_11_  =  33% for the formal “biradical” resonance structure of **S_2_N_2_** with a mere 14% singlet biradical character, or *w*_11_  =  61% for ozone with only 31% biradical character?

**FIGURE 11:**
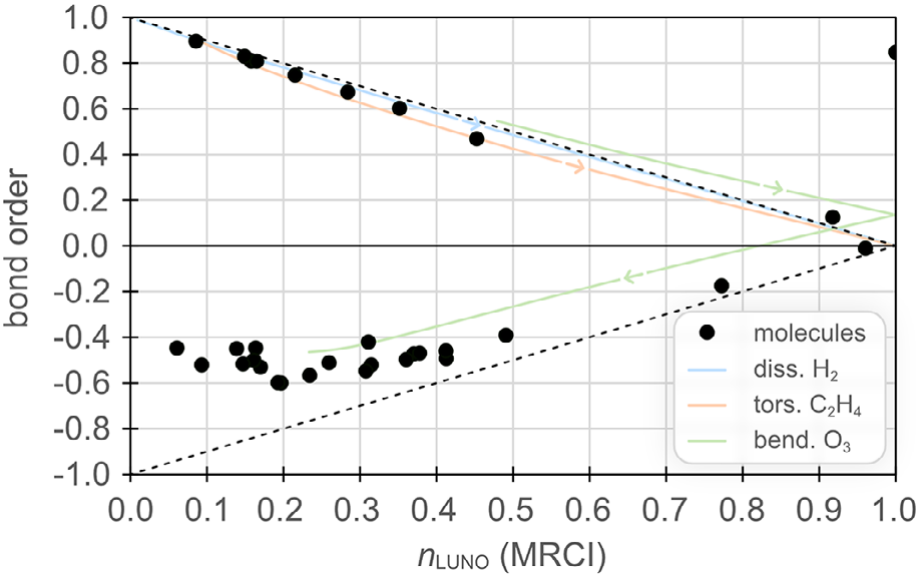
BO as determined from the CASSCF density matrix versus singlet biradical character *n*_LUNO_. The dissociation of H_2_, torsion of ethylene, and bending of O_3_ are included as model processes (cf. Supporting Information p. S64ff). The arrows indicate the direction of increasing H‐H distance, HCCH dihedral angle, and OOO bond angle, respectively. The black dashed line indicates the idealized relationship |BO|  =  1−*β*.

By analogy with the dissociation of H_2_, where at some point we arbitrarily switch between the two (mathematically identical) VB structures H–H and H· ·H, we must ask ourselves the question how to chemically interpret the “biradical” resonance structure of a singlet biradicaloid. Clearly, the key difference between H─H and H· ·H is our intuitive interpretation that one is bonding and the other is not. Thus, it stands to reason that in typical biradicaloids with intermediate biradical character, we should expect a partially bonding situation between the two radical electrons. We therefore evaluated the BO between the radical centers (as the off‐diagonal matrix elements of the CASSCF density matrix using localized orbitals) to obtain a deeper understanding of the interplay between BO and singlet biradical character (Figure 11 and Table 3), and in consequence the intuitive chemical interpretation of the electronic situation of biradical(oid)s.

**TABLE 3:** Singlet biradical character *n*_LUNO_ (MRCI), BO and weight *w*_11_ of the “biradical/covalent” resonance structure (A• •B, A•―•B, or A•⋯•B; vide infra).

	PG	*n*_LUNO_ (MRCI)	BO	*w*_11_
**N_2_P_2_**	*D*_2_ *_h_*	0.20	−0.60	0.59
**N_2_As_2_**	*D*_2_ *_h_*	0.23	−0.57	0.62
**N_2_Sb_2_**	*D*_2_ *_h_*	0.31	−0.52	0.70
**N_2_Bi_2_**	*C*_2_ *_h_*	0.37	−0.47	0.71
**P_2_C_2_^Cl^**	*C_i_*	0.26	−0.51	0.59
**P_2_C_2_**	*C_i_*	0.16	−0.45	0.36
**P_2_B_2_**	*D*_2_ *_h_*	0.16	+0.81	0.78
**P_2_Al_2_**	*D*_2_ *_h_*	0.09	+0.90	0.72
**P_2_Ga_2_**	*C*_2_ *_h_*	0.17	+0.81	0.79
**S_2_N_2_**	*D*_2_ *_h_*	0.14	−0.45	0.33
**O_3_**	*C*_2_ *_v_*	0.31	−0.42	0.61
**N_2_C_2_**	*C_i_*	0.17	−0.53	0.46
**N_2_Si_2_**	*C*_2_ *_h_*	0.41	−0.49	0.87
**N_2_Si_2_**	*D*_2_ *_h_*	0.31	−0.55	0.76
**N_2_Ge_2_**	*C_i_*	0.36	−0.50	0.76
**N_2_Sn_2_**	*C_i_*	0.38	−0.47	0.74
**NOGe_2_**	*C*_2_	0.41	−0.46	0.80
**O_2_Ge_2_**	*C*_2_ *_h_*	0.49	−0.39	0.84
**F_2_Ge_2_^2+^**	*C*_2_ *_h_*	0.77	−0.17	0.96
**Si_2_Si_2_^I^**	*D*_2_ *_h_*	0.15	+0.83	0.78
**Si_2_Si_2_**	*D*_2_ *_h_*	0.22	+0.75	0.82
**P_2_P_2_**	*C*_2_ *_h_*	0.16	−0.50	0.38
**P_2_P_2_**	*C_s_*	0.15	−0.52	0.37
**N_2_PAs** ^a^	*C*_2_ *_v_*	0.19	−0.60	0.57
**N_2_PAs** ^b^	*C*_2_ *_v_*	0.02	—	—
**N_2_CP** ^a^	*C*_2_	0.09	−0.52	0.24
**N_2_CP** ^c^	*C*_2_	0.04	—	—
**C_2_P_2_^CH2^**	*D*_2_ *_h_*	0.96	−0.01	0.79
**C_2_P_2_^NHC1^**	*C*_2_ *_h_*	0.27^d^	−0.50	0.59
**C_2_P_2_^NHC2^**	*D*_2_ *_h_*	0.22^d^	−0.52	0.51
**O_2_**	*D*_∞_ *_h_*	1.02	0.00	0.77
**C_2_C_2_**	*C*_2_ *_h_*	0.92	+0.13	0.90
**C_2_C_2_^F^**	*D*_2_ *_h_*	0.28	+0.67	0.86
**C_2_C_2_^OH^**	*D*_2_	0.35	+0.60	0.88
**C_3_C_2_^F^**	*C*_2_ *_v_*	0.45	+0.47	0.85
**Allyl^−^**	*C*_2_ *_v_*	0.06	−0.45	0.15

^a^ 1,3‐diyl.

^b^ arsinidene.

^c^ phosphinidene.

^d^ CASSCF(2,2) results.

As to be expected from the simple 2e2o model (black dashed line in Figure 11), the BO correlates with singlet biradical character. In fact, in the 2e2o model, |BO|  =  1−*β*, so three distinct groups of molecules can be defined (which may be regarded as an extension of Abe's type I/II concept): [17] 1) those with a bonding interaction between the radical centers, 2) those with no interaction and 3) those with an antibonding interaction. Most of the molecules investigated in our study belong to the last group.

Now, it is important to realize that the BO describes the (anti)bonding interaction between the “radical centers”, that is those *atoms* where it is most likely to find the radical electrons. Thus, it must be distinguished from the bonding interaction between the *radical electrons* themselves, which may be delocalized over a group of atoms. The simple 2e2o model can only describe this interaction between the *radical electrons*, so it may be regarded as a limit that can only be reached if the radical electrons are perfectly localized on the *radical centers*. As can be inferred from Figure 11, this is mostly the case for the bonding biradicaloids, but not for the antibonding ones. Since the antibonding character can be traced back to the so‐called “through‐bond interaction”, which describes the orbital interaction between the radical centers and the bridging atoms [10, 14, 17, 18] (i.e., a type of delocalization), the radical electrons in the antibonding biradicaloids can never be “perfectly” localized, so the magnitude of the antibonding character must be smaller than the ideal value. Note that this effect is independent of any biradical character and comparable to, for example, delocalized π‐bonding systems, where the BO of individual π‐bonds is also decreased due to delocalization.

**FIGURE 12:**
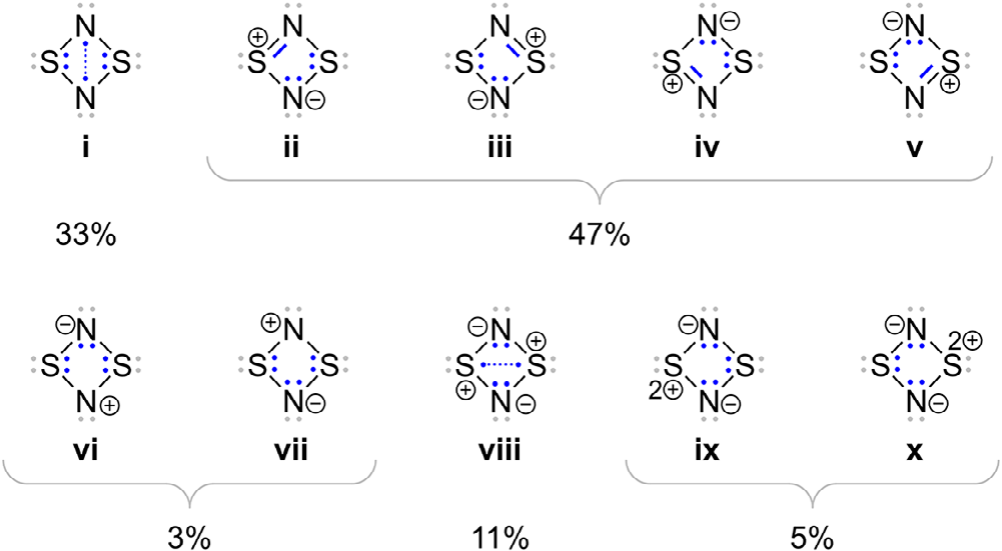
Lewis‐type resonance structures and weights according to the localization of the CASSCF(6,4)/def2‐TZVP wavefunction of S_2_N_2_. The π electrons are highlighted in blue. •⋯• = single π‐antibond (threshold: |BO/*w_i_* | > 0.2). Sums of weights are reported for symmetry equivalent structures.

With these considerations in mind, we can now amend the “biradical” Lewis structures of **S_2_N_2_** in Figure 9 and reinterpret the Lewis resonance scheme (Figure 12): With a total BO of −0.45 (−0.38) between the two N (S) atoms, the “biradical” resonances should be understood as single π‐antibonds (structures **i** and **viii**), which possess a low singlet biradical character. This view immediately resolves the apparent discrepancy between low biradical character and large weight of the “biradical” resonances—simply because they represent antibonds instead of two weakly coupled radical electrons. In total, the antibond resonances have a combined weight of 44%, which is in good agreement with the weight allocated to “biradical” resonances in previous literature reports [106, 115, 121]. The remaining resonance structures correspond to the “classical” delocalized resonances with S−N double bond character (**ii**‐**v**) as well as zwitterionic structures (**vi**, **vii**, **ix**, **x**) associated with the low singlet biradical character (14%) of the N⋯N as well as S⋯S antibonds. This low biradical character agrees with literature reports that investigated the biradical character of **S_2_N_2_** based on delocalized orbital descriptors [105, 108]. Therefore, we feel confident that our interpretation of the electronic structure of **S_2_N_2_** can reconciliate the previously opposing descriptions. Nonetheless, **S_2_N_2_** remains a remarkable molecule with an unusual electronic structure—a single π‐antibond is certainly not commonplace. Incidentally, it also nicely explains the planar structure of the molecule, as the antibonded atoms attempt to maximize their distance (N⋯N 2.36, S⋯S 2.32 Å; cf. Σ*r*_cov_: N‐N 1.42, S‐S 2.06 Å) [122].

**FIGURE 13:**
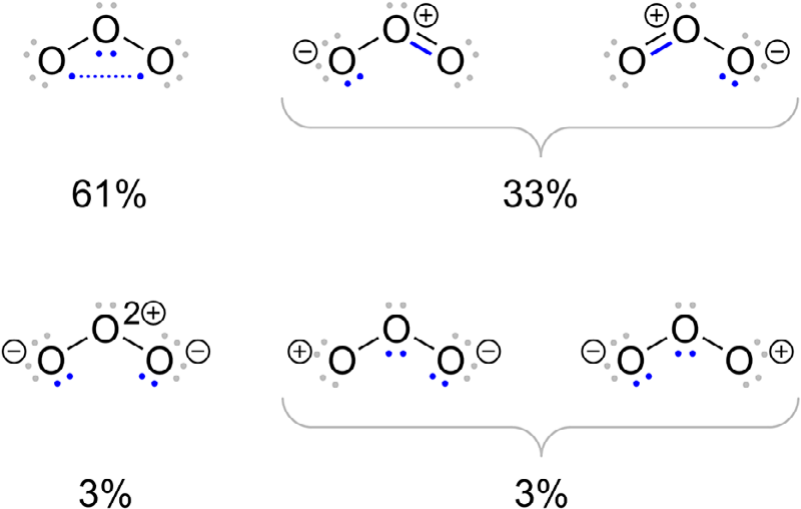
Lewis‐type resonance structures and weights according to the localization of the CASSCF(4,3)/def2‐TZVP wavefunction of O_3_. The π electrons are highlighted in blue. •⋯• = single π‐antibond (threshold: |BO/*w_i_* | > 0.2). Sums of weights are reported for symmetry equivalent structures.

Similar deliberations may be made for all the other singlet biradicaloids we investigated: Ozone, for example, has a significantly less delocalized electronic structure, in accordance with its higher biradical character of 31%. The leading resonance structure with 61% weight describes a single π‐antibond between the terminal O atoms with a BO of −0.42 (Figure 13). Note that the BO is quite similar to that of **S_2_N_2_** despite the much larger weight of the antibonding resonance, so clearly the significant biradical character reduces the magnitude of the BO. Ozone is therefore well described by the notion of a single π‐antibond with significant biradical character between the terminal O atoms.

**FIGURE 14:**
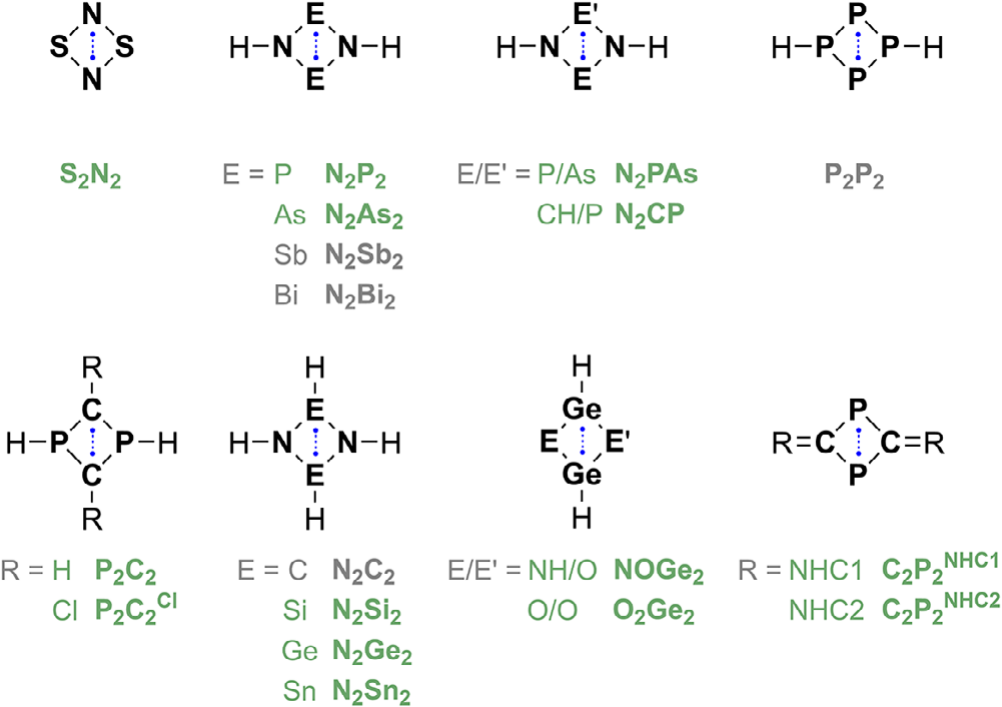
Leading resonance structures of four‐membered cyclic biradicaloids with a single π‐antibond (•⋯•). In those instances where the substituents are bent out of the ring plane (such as P_2_C_2_ or N_2_Si_2_), it might be regarded as a “nonclassical” or distorted π‐antibond. The structural motifs highlighted in green have previously been observed in experiments (cf. Figure 3).

Many of the four‐membered singlet biradicaloids in our study are formally isolobal to **S_2_N_2_**. Their resonance schemes look similar, albeit with different weights of the individual resonance structures (Figure 14 and Scheme S1). In most cases, the weight of the leading resonance structure is 60% or larger. The electronic situation is therefore well described by a single π‐antibond with varying amounts of biradical character between the radical centers, comparable to the situation in ozone.

**FIGURE 15:**
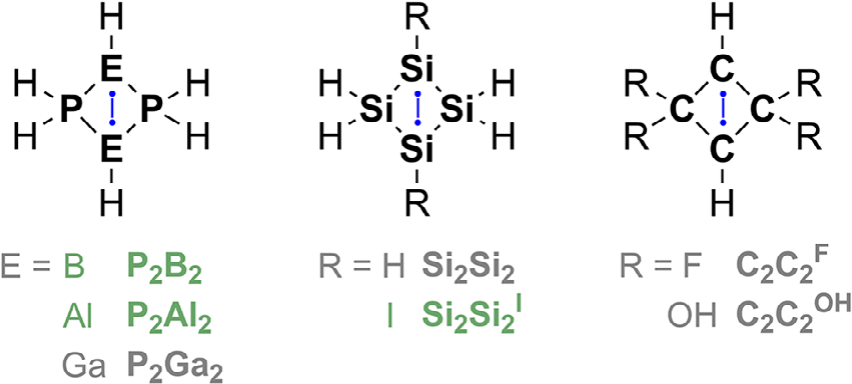
Leading resonance structures of four‐membered cyclic biradicaloids with a single π‐bond (•―•). The structural motifs highlighted in green have previously been observed in experiments (cf. Figure 3).

Notable exceptions are **P_2_B_2_**, **P_2_Al_2_**, **P_2_Ga_2_**, **Si_2_Si_2_**, **Si_2_Si_2_^I^**, **C_2_C_2_^F^**, and **C_2_C_2_^OH^** which have a single π‐bond between the radical centers (and it is this π‐bond that possesses biradical character, Figure 15). In fact, isolated derivatives of **Si_2_Si_2_** with bulky substituents have previously been described as molecules with an unsupported π‐bond between the Si atoms [48, 49], a concept that is also related to the idea of short‐bond and long‐bond isomers in such tetrasilicon ring systems [96]. Our description of biradical character supports this view, with the added benefit of being able to quantify the biradical character in those systems, which is low to moderate (15–22%, Table 3). The notion of a single π‐bond also explains why the parent ring systems with H substituents are saddle points on the PES and can only be stabilized by sterically demanding substituents that provide sufficient Pauli repulsion, so the ring system cannot distort and form a σ‐bond between the radical centers. Notice that the biradicaloids with a single π‐bond have formally sp^3^‐hybridized bridging atoms, contrary to the biradicaloids with a π‐antibond (Figure 14), where the bridging atoms are, at most, sp^2^‐hybridized.

**FIGURE 16:**
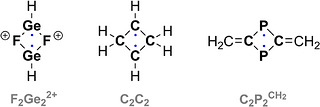
Leading resonance structures of four‐membered cyclic biradicaloids *without* a significant (anti)bonding interaction between the radical centers. C_2_C_2_ and C_2_P_2_^CH2^ have a triplet ground state, but here we discuss the singlet state.

Do all singlet biradicals have a bond or antibond between the radical centers? No—but at least among the four‐membered ring systems we studied, nonbonded biradicals are the exception (Figure 16). Of course, just as the transition between closed‐shell and “perfect” biradical is continuous, there is also no hard boundary between (anti)bonding and nonbonding situations. If we had to pinpoint a value, we would argue that |BO| < 0.2 qualifies as (nearly) nonbonding, but we recognize that this is a somewhat arbitrary choice. To also take into account delocalization effects which might reduce the BO, we propose a threshold of |BO/*w*_11_| < 0.2 for classifying a biradical interaction as nonbonding—or at least not significantly (anti)bonding.

Most notably, the three molecules in Figure 16 are also those with the highest singlet biradical character (≥77%). It therefore makes perfect sense that these systems do not exhibit a significant (anti)bonding interaction between the radical centers. Please also notice that **C_2_C_2_** and **C_2_P_2_^CH2^** possess a *triplet* ground state, but we discuss the properties of their lowest excited *singlet* state.

It is worth noting that biradicaloids with heavier radical centers tend to have a higher biradical character than their lighter analogs, although there are exceptions to this rule (e.g., **P_2_B_2_** 16% versus **P_2_Al_2_** 9%). As mentioned before, increasing electronegativity of the bridging atoms typically also increases the biradical character, as exemplified by the (rather hypothetical) ion **F_2_Ge_2_^2+^** (77%).

**FIGURE 17:**
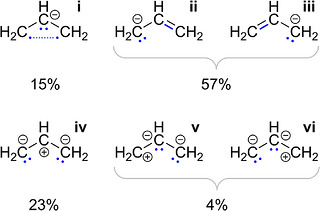
Lewis‐type resonance structures and weights according to the localization of the CASSCF(4,3)/def2‐TZVP wavefunction of Allyl^−^. The π electrons are highlighted in blue. •⋯• = single π‐antibond (threshold: |BO/*w_i_* | > 0.2). Sums of weights are reported for symmetry equivalent structures.

Another very instructive example that we would like to discuss briefly is the allyl anion, which is formally isolobal to ozone. With a singlet biradical character of only 6%, it is undoubtedly a closed‐shell species (the biradical character can be attributed to dynamical correlation). Still, we can identify a Lewis resonance structure with a single π‐antibond between the terminal C atoms with 15% contribution to the overall bonding situation (structure **i**, Figure 17). As expected, the zwitterionic resonances **iv**‐**vi** have much larger weights than in the case of ozone (cf. Figure 13) due to the closed‐shell character of the allyl anion. Thus, the *combination* of the pure “covalent” resonance **i** and the zwitterionic resonances describe a closed‐shell π‐antibond. (Remember that closed‐shell H_2_ is also described by the resonances H–H, ^−^H: H^+^, and ^+^H :H^−^.) This example goes to show that our approach covers the whole range of electronic structures, from closed‐shell species to “perfect” biradicals. Moreover, antibonds are not restricted to “exotic” biradicaloid species but can also be of use in the description and interpretation of the electronic structure of “regular” closed‐shell molecules. In this regard, it is also of interest that the BO between the two terminal C atoms is significantly antibonding (−0.45), which is corroborated by the natural localized molecular orbital (NLMO) [123, 124, 125] BO obtained from DFT calculations (PBE‐D3/aug‐cc‐pVTZ), which amounts to −0.50. Again, the large antibonding character can be traced back not only to the pure “covalent” antibond resonance **i**, but also to contributions from the zwitterionic resonances (in particular **v** and **vi**, partly also **iv**), which together describe the closed‐shell π‐antibond. Consequently, in addition to the textbook resonances **ii** and **iii**, a π‐antibond of type **i** should also be included in the most important resonance structures. Notice that this antibond can very elegantly explain the large CCC bond angle of 131.8° (CCSD(T)/aug‐cc‐pVTZ), which is significantly larger than the ideal *sp* ^2^ bond angle of 120°.

Additionally, we would like to point out that singlet dioxygen has a biradical character of 100% but a π BO of +0.84. This is due to the fact that both π_x_ and π_y_ orbitals are doubly occupied, while the π_x_* and π_y_* orbitals are only singly occupied. This results in an idealized BO of +1, which is close to the computed value.

On a final note, it is of course possible to extend our biradical descriptors to molecules with multiple biradicaloid functionalities, such as bis(biradicaloids) or tetraradicaloids [91]. For example, we calculated both the 1,3‐diyl biradical character as well as the phosphinidene‐type biradical character for **N_2_CP**, which may be regarded as a masked singlet phosphinidene based on the reactivity of [PhC(μ‐NBhp)_2_P] [52, 126]. In both instances, the biradical character is negligible (9% or 4%, respectively), and also the triplet states corresponding either to a 1,3‐diyl (Δ*E*_ST_^v^  = −245.6 kJ/mol) or a phosphinidene (−199.1 kJ/mol) are high in energy. The transannular interaction between C and P is therefore well described as a (delocalized) single π‐antibond, with a BO of −0.52. The lone pair at the P atoms is a “regular” lone pair without significant biradical/phosphinidene character.

**FIGURE 18:**
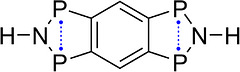
Example of a tetraradicaloid with two single π‐antibonds.

Accordingly, we can use the existing data of a recently reported tetraradicaloid [91] to interpret the electronic structure in a similar way (Figure 18): Both P‐P transannular bonds may be viewed as single π‐antibonds (BO −0.48), with considerable biradical (26%) and in this case also tetraradical character (19%) due to electronic coupling. Because of this coupling of four electrons, the derivation of the exchange coupling constants is a bit more complicated. The short‐range exchange coupling, which is comparable to the ST gap in case of biradicaloids, amounts to −149.9 kJ/mol, which aligns well with the data for the four‐membered ring systems discussed before.

A full list of resonance structures for all molecules we investigated may be found in the Supporting Information (Schemes S1 and S2). Additionally, a summary of all biradical indicators is presented in Table S5.

## 4 Conclusion

The electronic structure of small singlet biradicaloids was systematically investigated. From the computed data, a set of robust biradical indicators was identified, which have complementary meaning and should be considered together. We recommend them for general use:

1. First, the vertical singlet‐triplet (ST) gap is a measure of the magnetic coupling of the electrons, while the adiabatic ST gap indicates the thermal accessibility of the triplet state (or singlet state if the triplet is the ground state).
2. Second, the LUNO occupancy indicates singlet biradical character, which describes whether the radical electrons act more like electron pairs (low biradical character) or like single electrons (high biradical character). Both ST gap and biradical character can influence whether it is likely to observe stepwise, radical‐like reactions. Please note, however, that there is only a loose correlation between ST gap and singlet biradical character, so a smaller energy difference between the two states does not necessarily indicate a higher singlet biradical character or vice versa.
3. Third, the BO between the radical centers is maybe the most important quantity to foster an intuitive understanding of the electronic structure of singlet biradicaloids (or polyradicaloids in general). In conjunction with a Lewis‐type resonance scheme, it provides context to the interpretation of the “biradical” resonance structure(s). We find that in the majority of the molecules we investigated, the leading resonance should be interpreted in terms of either a single (stretched) π‐bond or single (compressed) π‐antibond between the radical centers. It is exactly this (anti)bond to which the biradical character should be assigned; thus, the situation of this (anti)bond is comparable to that of stretched H_2_, which is in the process of bond breaking.

To the best of our knowledge, this interpretation using bonds and antibonds has not been used rigorously before. It allows us to resolve a long‐standing dispute over the biradical character of S_2_N_2_, which we view as a molecule with low biradical character and a delocalized π‐antibond between both N or both S atoms. Also, the description of the electronic structure of other well‐known biradicaloids such as ozone benefits from our interpretation, which intuitively connects the Lewis resonance scheme to the singlet biradical character.

Furthermore, our proposed set of biradical descriptors covers the whole range from closed‐shell molecules to perfect biradicals. Application of our proposed model to the closed‐shell allyl anion, for example, reveals that the interpretation of its electronic structure may be extended to include a resonance structure with a single π‐antibond between the terminal C atoms, next to the textbook description of its delocalized π‐bonding system. The π‐antibond can intuitively explain the large CCC bond angle. If antibonds also play a role in other closed‐shell systems will have to be investigated in the future. Yet, our current findings imply that antibonds may be a previously overlooked bond type in Lewis‐type resonance structures, particularly in singlet biradicaloids with low to moderate biradical character and close proximity between the radical centers.

Our findings imply that structure optimization using a pure density functional such as PBE‐D3 and a CASSCF calculation with a small to moderately‐sized active space give reasonably accurate results compared to our high‐level ab‐initio calculations. Hence, this is our recommended approach to investigate the biradical character (i.e., Δ*E*_ST_ from DFT as well as *n*_LUNO_ and BO from the CASSCF) of larger molecules, for example biradicaloids with sterically demanding substituents. Please note that the computation of BOs between the radical centers in species with largely delocalized radical electrons, such as polyaromatic ring systems, will probably still pose a challenge, though, due to the necessity of large active spaces to obtain fully localized orbitals.

Finally, we find that biradical character is an intricate interplay between different properties of the electronic structure, and in particular, it does not only include the bonding, but also the antibonding regime. *Hence, only a combination of the different descriptors of biradical character allows for a complete understanding of the electronic structure of biradical(oid)s*.

## Conflicts of Interest

The authors declare no conflicts of interest.

## Supporting information



The Supporting Information (PDF) contains detailed information about computational methods, a comprehensive summary of all calculated data, pictures of all optimized structures, depictions of the active space orbitals of CAS reference wavefunctions, Lewis resonance schemes of all molecules including the relative weights, details on the description of the dissociation of H_2_, torsion of ethylene and bending of O_3_, the step‐by‐step derivation of eqn. 20, and lastly, additional biradical indices. Additional references are cited in the Supporting Information [127, 128, 129, 130, 131, 132, 133, 134, 135, 136, 137, 138, 139, 140, 141, 142]. All optimized structures can be found in the XYZ file. **Supporting File 1**: chem70968‐sup‐0001‐SuppMat.pdf.

**Supporting File 2**: chem70968‐sup‐0002‐SuppMat.xyz.
