# Supplementary material for: Bond and Antibond Resonances: A Unified Framework for Singlet Biradical Character
Source: Chemistry. 2026 Apr 8;32(23):e70968. doi: 10.1002/chem.70968 (PMC13282919; doi:10.1002/chem.70968)
Supplement: Supplementary file 1 — The Supporting Information (PDF) contains detailed information about computational methods, a comprehensive summary of all calculated data, pictures of all optimized structures, depictions of the active space orbitals of CAS reference wavefunctions, Lewis resonance schemes of all molecules including the relative weights, details on the description of the dissociation of H2, torsion of ethylene and bending of O3, the step‐by‐step derivation of eqn. 20, and lastly, additional biradical indices. Additional references are cited in the Supporting Information [127, 128, 129, 130, 131, 132, 133, 134, 135, 136, 137, 138, 139, 140, 141, 142]. All optimized structures can be found in the XYZ file. Supporting File 1: chem70968‐sup‐0001‐SuppMat.pdf. [file CHEM-32-e70968-s001.pdf]

SUPPORTING INFORMATION

Bond and Antibond Resonances: A Unified Framework for Singlet Biradical Character

*Daniel T. Gschwind, Jonas Bresien**

This file includes:

1 General remarks S2
2 Summary of calculated data S4
3 Optimized structures S24
4 Vibrational frequencies S38
5 Active space orbitals S48
6 Lewis resonance schemes S58
7 Model processes varying the biradical character S64
8 Derivation of equation 20 ..... S69
9 Additional biradical indices S70
10 References S72

1 General remarks

Computations were carried out using Gaussian09 (rev. D.01),[1] Gaussian16 (rev. C.02),[2] ORCA 5.0.4[3–6] and the standalone version of NBO 6.0.[7–10]

DFT structure optimizations using analytic gradients employed the pure exchange-correlation functional PBE[11,12] in conjunction with Grimme's dispersion correction D3(BJ)[13,14] and the def2-TZVP basis set[15] (notation (U)PBE-D3/def2-TZVP). The resolution of identity (RI) approximation was applied, using Weigend's accurate Coulomb-fitting basis.[16] Numerical integration of the exchange-correlation energy was performed on Gaussian's "ultrafine" grid or ORCA's default XC grid ("DefGrid2"). The stability of all singlet Kohn-Sham wavefunctions was analysed, and the unrestricted "broken symmetry" solution was used where appropriate. Triplet states were calculated using an unrestricted approach. All structures were fully optimized and confirmed as minima (or transition states) by analytic frequency analyses.

The PBE functional has proven to yield reasonably accurate results for main-group biradicaloids.[17–19] Being a pure (i.e. non-hybrid) functional, it is somewhat more robust which respect to non-dynamical correlation than comparable hybrid functionals, at least when dealing with low to moderate biradical character.[20–22]

Ab-initio structure optimizations were performed at the (U)CCSD(T)[23–27]/def2-TZVP level of theory using numerical gradients. All singlet ground states were computed using an RHF reference, and a UHF reference was used otherwise (i.e. for all triplet and excited singlet states; cf. Table S2). All structures were fully optimized and confirmed as minima (or transition states) by numerical frequency analyses. CCSD(T) was chosen despite being a single-reference method, as it is known to be quite robust even in cases of considerable multi-reference character.[22] The T_1 diagnostic was evaluated in each case to assess the multireference character of the wavefunction and verify that the

single-reference coupled-cluster approach is viable (empirically, CCSD(T) results are considered reliable if $T_1 < 0.02$[22,28] for closed-shell reference wavefunctions[29,30]).

Multi-determinantal wavefunctions that correctly describe the multireference character of the investigated systems (correct treatment of non-dynamical correlation) were computed with ORCA using the Complete Active Space SCF (CASSCF) method[31–34] and a triple-zeta basis set (def2-TZVP).[15] The RI approximation was used to speed up calculations, utilizing the appropriate auxiliary basis set (def2-TZVP/C).[35] To account for dynamical correlation, the CASSCF reference was employed in multireference perturbation calculations, using the Strongly Contracted *N*-Electron Valence State Perturbation Theory (SC-NEVPT2).[36–38] Alternatively, the CASSCF reference was used in multireference configuration interaction (MRCI) calculations with single and double substitutions. The perturbative multi-reference Davidson correction for disconnected quadruple substitutions was applied (i.e. MRCISD+Q).[39,40] All single-point multi-reference calculations were performed using the optimized structures at the CCSD(T)/def2-TZVP level of theory, unless noted otherwise.

Please note that we chose to investigate only proton-substituted model systems of literature-known and hypothetical biradicaloids. Substituent effects, especially sterical effects that stabilize additional isomers, are therefore not represented in our computations.

2 Summary of calculated data

We started our investigation with model systems of common, literature-known four-membered cyclic biradicaloids, i.e. formal hetero-cyclobutanediyls.[41–55] With two exceptions,[56,57] only systems with two symmetry-equivalent radical centres were considered. In all cases, the (sterically demanding) substituents were substituted by H atoms, in order to use high-level ab-initio methods for the description of the electronic structure. In molecules with a bonding interaction between the radical centres, the biradicaloid structure is typically stabilized by these sterically demanding substituents (e.g. by Pauli repulsion between the bulky groups),[49,53,58] so the model structures correspond to saddle points on the potential energy surface. Nonetheless, as the stabilization is mainly attributed to sterics, the electronic structure is appropriately represented by the models.

During the course of our study, we arbitrarily added some additional molecules (O_3, O_2, allyl anion) as well as some hypothetical biradicaloids to test our hypotheses. Please note that the list of investigated systems is not intended to be "complete", the examples are merely chosen to be representative of the investigated compound class.

In Tables S1-S2, the relevant thermodynamic data of the investigated molecules are summarized. For most molecules, three sets of energies are reported: The first entry corresponds to the optimized ground state structure (which is derived from the corresponding experimental structure). The second entry corresponds to a spin-flip of one of the radical electrons at the ground state equilibrium geometry (i.e., it is used to calculate the vertical singlet-triplet gap). The third entry describes the optimized structure of the spin-flip state that most closely resembles the ground state structure (esp. with respect to *syn*- or *anti*-arrangement of the substituents). In ambiguous cases, the lowest-energy conformer is reported (based on E_{tot}).

Table S1. Electronic and thermal energies (in a.u.) computed at the (U)PBE-D3/def2-TZVP level of theory (allyl anion at (U)PBE-D3/aug-cc-pVTZ[59] with automatically generated density fitting basis;[1] the augmented basis set was used for an improved description of the anionic charge). M = multiplicity; PG = point group; N_{imag} = number of imaginary frequencies; $\langle S^2 \rangle$ = expectation value of the S^2 operator; E_{tot} = electronic (total) energy; U_0 = inner energy at 0 K (E_{tot} + ZPE); U_{298}, H_{298}, G_{298} = inner energy, enthalpy, free enthalpy at 298.15 K.

Compd.	M	PG	N_{imag}	$\langle S^2 \rangle$	E_{tot}	U_0	U_{298}	H_{298}	G_{298}
$[\mathrm{P}(\mu\text{-NH})]_2$	1	D_{2h}	0	0	−793.0524	−793.0202	−793.0156	−793.0147	−793.0468
$\mathbf{N_2P_2}$	3	D_{2h}		2.01	−792.9923				
	3	C_{2v}	0	2.01	−793.0044	−792.9727	−792.9683	−792.9674	−793.0006
$[\mathrm{As}(\mu\text{-NH})]_2$	1	C_{2h}	0	0	−4581.7165	−4581.6864	−4581.6815	−4581.6805	−4581.7156
$\mathbf{N_2As_2}$	3	C_{2h}		2.01	−4581.6673				
	3	C_{2v}	0	2.00	−4581.6804	−4581.6507	−4581.6459	−4581.6449	−4581.6809
$[\mathrm{Sb}(\mu\text{-NH})]_2$	1	C_{2h}	0	0	−591.1442	−591.1158	−591.1104	−591.1095	−591.1467
$\mathbf{N_2Sb_2}$	3	C_{2h}		2.01	−591.1122				
	3	C_{2v}	0	2.00	−591.1197	−591.0916	−591.0863	−591.0853	−591.1236
$[\mathrm{Bi}(\mu\text{-NH})]_2$	1	C_{2h}	0	0	−539.9632	−539.9359	−539.9303	−539.9293	−539.9683
$\mathbf{N_2Bi_2}$	3	C_{2h}		2.00	−539.9365				
	3	C_{2v}	0	2.00	−539.9446	−539.9176	−539.9120	−539.9110	−539.9513
$[\mathrm{ClC}(\mu\text{-PH})]_2$	1	C_i	0	0	−1679.7149	−1679.6853	−1679.6779	−1679.6769	−1679.7182
$\mathbf{P_2C_2^{Cl}}$	3	C_i		2.01	−1679.6819				
	3	C_{2h}	0	2.01	−1679.7098	−1679.6808	−1679.6731	−1679.6722	−1679.7152
$[\mathrm{HC}(\mu\text{-PH})]_2$	1	C_i	0	0	−760.8522	−760.8056	−760.8005	−760.7995	−760.8335
$\mathbf{P_2C_2}$	3	C_i		2.01	−760.8002				
	3	C_{2h}	0	2.01	−760.8297	−760.7842	−760.7790	−760.7780	−760.8127
$[\mathrm{HB}(\mu\text{-PH_2})]_2$	1	D_{2h}	1	0	−735.5856	−735.5245	−735.5196	−735.5187	−735.5511
$\mathbf{P_2B_2}$	3	D_{2h}		2.01	−735.5460				
	3	C_{2h}	0	2.01	−735.5535	−735.4953	−735.4887	−735.4878	−735.5254
$[\mathrm{HAl}(\mu\text{-PH_2})]_2$	1	D_{2h}	1	0	−1170.5990	−1170.5493	−1170.5420	−1170.5411	−1170.5790
$\mathbf{P_2Al_2}$	3	D_{2h}		2.01	−1170.5506				
	3	C_{2h}	0	2.01	−1170.5869	−1170.5383	−1170.5298	−1170.5289	−1170.5715
$[\mathrm{HGa}(\mu\text{-PH_2})]_2$	1	C_{2h}	2	0	−4535.1184	−4535.0698	−4535.0628	−4535.0618	−4535.1015
$\mathbf{P_2Ga_2}$	3	C_{2h}		2.00	−4535.0836				
	3	C_{2h}	0	2.00	−4535.1103	−4535.0625	−4535.0535	−4535.0526	−4535.0979
$[\mathrm{N}(\mu\text{-S})]_2$	1	D_{2h}	0	0	−905.4384	−905.4286	−905.4250	−905.4241	−905.4541
$\mathbf{S_2N_2}$	3	D_{2h}		2.01	−905.3240				
	3	C_{2v}	0	2.01	−905.3469	−905.3393	−905.3353	−905.3343	−905.3669
$\mathbf{O_3}$	1	C_{2v}	0	0	−225.3246	−225.3178	−225.3148	−225.3139	−225.3410
[a]	3	C_{2v}		2.00	−225.2543				
[a]	3	C_{2v}	0	2.00	−225.2726	−225.2685	−225.2649	−225.2639	−225.2934

[a] Note that this state ($^3B_2$ state) is not the lowest triplet state at this level of theory. It is, however, the correct triplet state that corresponds to the singlet biradical character of the ground state (i.e., the electrons are localized in the same orbitals).

Table S1 continued.

Compd.	*M*	PG	N_{imag}	$\langle S^2 \rangle$	E_{tot}	U_0	U_{298}	H_{298}	G_{298}
[HC(μ-NH)]$_2$	1	C_i	0	0	−187.8659	−187.8051	−187.8011	−187.8002	−187.8306
**N_2C_2**	3	C_i		2.00	−187.7751				
	3	C_1	0	2.01	−187.7941	−187.7355	−187.7313	−187.7304	−187.7625
[HSi(μ-NH)]$_2$	1	C_{2h}	0	0.04	−690.5487	−690.5033	−690.4975	−690.4966	−690.5317
**N_2Si_2**	3	C_{2h}		2.00	−690.5215				
	3	C_i	0	2.00	−690.5226	−690.4768	−690.4714	−690.4704	−690.5061
[b]	1	D_{2h}	2	0.04	−690.4671	−690.4228	−690.4176	−690.4167	−690.4503
[c]	3	D_{2h}		2.01	−690.4417				
[HGe(μ-NH)]$_2$	1	C_i	0	0	−4265.1937	−4265.1514	−4265.1453	−4265.1443	−4265.1819
**N_2Ge_2**	3	C_i		2.00	−4265.1569				
	3	C_i	0	2.00	−4265.1594	−4265.1175	−4265.1113	−4265.1104	−4265.1493
[HSn(μ-NH)]$_2$	1	C_i	0	0	−540.4005	−540.3619	−540.3550	−540.3541	−540.3944
**N_2Sn_2**	3	C_i		2.01	−540.3694				
	3	C_i	0	2.01	−540.3721	−540.3340	−540.3271	−540.3261	−540.3678
[O(μ-GeH)$_2$NH]	1	C_2	0	0	−4285.0758	−4285.0449	−4285.0391	−4285.0382	−4285.0746
**$NOGe_2$**	3	C_2		2.00	−4285.0452				
	3	C_1	0	2.00	−4285.0474	−4285.0167	−4285.0110	−4285.0101	−4285.0482
[HGe(μ-O)]$_2$	1	C_{2h}	0	0.08	−4304.9570	−4304.9372	−4304.9318	−4304.9309	−4304.9667
**O_2Ge_2**	3	C_{2h}		2.00	−4304.9353				
	3	C_{2h}	0	2.00	−4304.9365	−4304.9169	−4304.9115	−4304.9106	−4304.9476
[HGe(μ-F)]$_2^{2+}$	1	C_{2h}	0	0.89	−4353.3798	−4353.3644	−4353.3579	−4353.3570	−4353.3951
**$F_2Ge_2^{2+}$**	3	C_{2h}		2.01	−4353.3765				
	3	C_{2h}	0	2.01	−4353.3767	−4353.3615	−4353.3550	−4353.3541	−4353.3934
[ISi(μ-SiH$_2$)]$_2$	1	D_{2h}	2	0	−1755.2730	−1755.2357	−1755.2266	−1755.2257	−1755.2724
**$Si_2Si_2^{I}$**	3	D_{2h}		2.01	−1755.2279				
	3	C_{2h}	0	2.01	−1755.2610	−1755.2242	−1755.2133	−1755.2123	−1755.2674
[HSi(μ-SiH$_2$)]$_2$	1	D_{2h}	1	0	−1160.9499	−1160.9005	−1160.8935	−1160.8925	−1160.9308
**Si_2Si_2**	3	D_{2h}		2.01	−1160.9399	−1160.8914	−1160.8834	−1160.8824	−1160.9240
	3	C_{2h}	0	2.01	−1160.9206				
[P(μ-PH)]$_2$	1	C_{2h}	0	0	−1366.0358	−1366.0145	−1366.0087	−1366.0078	−1366.0447
**P_2P_2**	3	C_{2h}		2.01	−1365.9849				
	3	C_s	0	2.01	−1366.0116	−1365.9908	−1365.9851	−1365.9842	−1366.0219

[b] Optimized geometry in D_{2h} symmetry. [c] Note that this state ($^3B_{1u}$ state) is not the lowest triplet state at this level of theory. It is, however, the correct triplet state that corresponds to the singlet biradical character of the ground state (i.e., the electrons are localized in the same orbitals).

Table S1 continued.

Compd.	M	PG	N_{imag}	$\langle S^2 \rangle$	E_{tot}	U_0	U_{298}	H_{298}	G_{298}
[P(μ-NH)$_2$As]	1	C_2	0	0	−2687.3867	−2687.3558	−2687.3508	−2687.3498	−2687.3853
**N$_2$PAs** [d]	3	C_2		2.01	−2687.3267				
	3	C_s	0	2.01	−2687.3428	−2687.3120	−2687.3074	−2687.3065	−2687.3418
[e]	3	C_2		2.01	−2687.3297				
[e]	3	C_s	0	2.00	−2687.3540	−2687.3241	−2687.3188	−2687.3179	−2687.3552
[P(μ-NH)$_2$CH]	1	C_2	0	0	−490.4655	−490.4188	−490.4145	−490.4135	−490.4448
**N$_2$CP** [d]	3	C_2		2.01	−490.3816				
	3	C_s	0	2.01	−490.4010	−490.3553	−490.3512	−490.3503	−490.3829
[f]	3	C_2		2.01	−490.3898				
[f]	3	C_1	0	2.01	−490.4456	−490.3991	−490.3945	−490.3935	−490.4277
[P(μ-CCH$_2$)]$_2$	3	D_{2h}	0	2.03	−837.0098	−836.9474	−836.9407	−836.9397	−836.9782
**C$_2$P$_2$^{CH_2}**	1	D_{2h}		1.00	−837.0016				
	1	D_{2h}	0	1.00	−837.0018	−836.9395	−836.9326	−836.9317	−836.9697
[P(μ-CR^1)]$_2$	1	C_{2h}	0	0	−1213.0225	−1212.8198	−1212.8059	−1212.8049	−1212.8615
**C$_2$P$_2$^{NHC1}**	3	C_{2h}		2.01	−1212.9989				
	3	C_{2h}	0	2.01	−1213.0043	−1212.8030	−1212.7889	−1212.7879	−1212.8455
[P(μ-CR^2)]$_2$	1	D_{2h}	0	0	−1210.6220	−1210.4664	−1210.4529	−1210.4520	−1210.5072
**C$_2$P$_2$^{NHC2}**	3	D_{2h}		2.01	−1210.5872				
	3	C_{2h}	0	2.01	−1210.5913	−1210.4383	−1210.4240	−1210.4231	−1210.4811
**O$_2$**	3	$D_{\infty h}$	0	2.00	−150.2492	−150.2456	−150.2433	−150.2423	−150.2656
	1	$D_{\infty h}$		1.00	−150.2351				
[HC(μ-CH$_2$)]$_2$	3	D_{2h}	0	2.01	−155.7210	−155.6434	−155.6383	−155.6374	−155.6700
**C$_2$C$_2$**	1	D_{2h}		1.01	−155.7185				
[HC(μ-CF$_2$)]$_2$	1	D_{2h}	1	0	−552.5513	−552.5008	−552.4944	−552.4934	−552.5304
**C$_2$C$_2$^{F}**	3	D_{2h}		2.01	−552.5078				
	3	C_{2h}	0	2.01	−552.5185	−552.4693	−552.4620	−552.4611	−552.5015
[HC(μ-C(OH)$_2$)]$_2$	1	D_2	1	0.33	−456.4792	−456.3832	−456.3748	−456.3739	−456.4142
**C$_2$C$_2$^{OH}**	3	D_2		2.01	−456.4522				
	3	C_2	0	2.01	−456.4627	−456.3670	−456.3578	−456.3569	−456.4003
C$_5$H$_6$F$_2$	1	C_{2v}	0	0.42	−393.4551	−393.3617	−393.3549	−393.3540	−393.3926
**C$_3$C$_2$^{F}**	3	C_{2v}		2.01	−393.4337				
	3	C_2	0	2.01	−393.4374	−393.3447	−393.3377	−393.3367	−393.3761
[H$_2$CC(H)CH$_2$]$^-$	1	C_{2v}	0	0	−117.1574	−117.0959	−117.0915	−117.0906	−117.1201
[g]	3	C_{2v}		2.01	−117.0814				
[g]	3	C_{2v}	0	2.01	−117.0835	−117.0218	−117.0173	−117.0163	−117.0472

[d] Computed using TD-DFT.[60–63] [e] Arsinidene (i.e. both unpaired electrons mainly localized at As). [f] Phosphinidene (i.e. both unpaired electrons mainly localized at P). [g] Note that this state ($^3B_2$ state) is not the lowest triplet state. The unpaired electrons are located in the n and π^* orbitals.

Table S2. Electronic and thermal energies (in a.u.) computed at the (U)CCSD(T)/def2-TZVP level of theory (allyl anion at (U)CCSD(T)/aug-cc-pVTZ). M = multiplicity; PG = point group; N_{imag} = number of imaginary frequencies; $\langle S^2 \rangle$ = S^2 expectation value of HF reference; E_{tot} = electronic (total) energy; U_0 = inner energy at 0 K (E_{tot} + ZPE); U_{298}, H_{298}, G_{298} = inner energy, enthalpy, free enthalpy at 298.15 K; T_1 = T_1 diagnostic.

Compd.	M	PG	N_{imag}	$\langle S^2 \rangle$	E_{tot}	U_0	H_{298}	G_{298}	T_1
[P(μ-NH)]$_2$	1	D_{2h}	0	0	−792.3391	−792.3059	−792.3006	−792.3322	0.013
**N_2P_2**	3	D_{2h}		2.05	−792.2825				0.018
	3	C_{2v}	0	2.05	−792.2923	−792.2597	−792.2544	−792.2876	0.020
[As(μ-NH)]$_2$	1	D_{2h}	0	0	−4579.4550	−4579.4243	−4579.4182	−4579.4533	0.011
**N_2As_2**	3	D_{2h}		2.04	−4579.4082				0.014
	3	C_{2v}	0	2.04	−4579.4202	−4579.3895	−4579.3838	−4579.4197	0.016
[Sb(μ-NH)]$_2$	1	D_{2h}	0	0	−589.7328	−589.7036	−589.6971	−589.7342	0.014
**N_2Sb_2**	3	D_{2h}		2.04	−589.7022				0.017
	3	C_{2v}	0	2.04	−589.7091	−589.6802	−589.6740	−589.7121	0.019
[Bi(μ-NH)]$_2$	1	C_{2h}	0	0	−538.4470	−538.4189	−538.4124	−538.4512	0.015
**N_2Bi_2**	3	C_{2h}		2.03	−538.4226				0.018
	3	C_{2v}	0	2.03	−538.4306	−538.4028	−538.3963	−538.4363	0.020
[ClC(μ-PH)]$_2$	1	C_i	0	0	−1678.3873	−1678.3569	−1678.3485	−1678.3897	0.017
**$P_2C_2^{Cl}$**	3			2.07	−1678.3554				0.021
	3	C_{2h}	—[a]	2.07	−1678.3856	—[a]	—[a]	—[a]	0.021
[HC(μ-PH)]$_2$	1	C_i	0	0	−760.1440	−760.0960	−760.0900	−760.1241	0.016
**P_2C_2**	3	C_i		2.07	−760.0929				0.022
	3	C_{2h}	0	2.06	−760.1250	−760.0779	−760.0719	−760.1065	0.023
[HB(μ-PH$_2$)]$_2$	1	D_{2h}	1	0	−734.8968	−734.8331	−734.8274	−734.8595	0.016
**P_2B_2**	3	D_{2h}		2.03	−734.8535				0.015
	3	C_{2h}	0	2.03	−734.8602	−734.7994	−734.7920	−734.8291	0.015
[HAl(μ-PH$_2$)]$_2$	1	D_{2h}	1	0	−1169.3568	−1169.3048	−1169.2967	−1169.3345	0.014
**P_2Al_2**	3	D_{2h}		2.04	−1169.3047				0.016
	3	C_{2h}	0	2.01	−1169.3470	−1169.2961	−1169.2868	−1169.3286	0.020
[HGa(μ-PH$_2$)]$_2$	1	C_{2h}	2	0	−4532.2877	−4532.2367	−4532.2289	−4532.2682	0.015
**P_2Ga_2**	3	C_{2h}		2.02	−4532.2523				0.016
	3	C_{2h}	0	2.02	−4532.2822	−4532.2319	−4532.2222	−4532.2668	0.017
[N(μ-S)]$_2$	1	D_{2h}	0	0	−904.7158	−904.7058	−904.7013	−904.7313	0.015
**S_2N_2**	3	D_{2h}		2.04	−904.6034				0.021
	3	C_{2v}	0	2.06	−904.6230	−904.6154	−904.6104	−904.6430	0.033
**O_3**	1	C_{2v}	0	0	−225.1419	−225.1353	−225.1313	−225.1584	0.028
	3	C_{2v}		2.02	−225.0827				0.022
	3	C_{2v}	0	2.03	−225.0978	−225.0927	−225.0886	−225.1172	0.026
[HC(μ-NH)]$_2$	1	C_i	0	0	−187.6813	−187.6191	−187.6142	−187.6446	0.011
**N_2C_2**	3	C_i		2.02	−187.5944				0.014
	3	C_1	0	2.02	−187.6144	−187.5537	−187.5487	−187.5808	0.016

[a] No frequency analysis was performed.

Table S2 continued.

Compd.	M	PG	N_{imag}	$\langle S^2 \rangle$	E_{tot}	U_0	H_{298}	G_{298}	T_1
[HSi(μ-NH)]$_2$	1	C_{2h}	0	0	−689.8474	−689.7997	−689.7934	−689.8272	0.016
**N_2Si_2**	3	C_{2h}		2.01	−689.8218				0.015
	3	C_i	0	2.01	−689.8222	−689.7745	−689.7683	−689.8038	0.016
[b]	1	D_{2h}	2	0	−689.7597	−689.7134	−689.7076	−689.7402	0.014
	3	D_{2h}		2.05	−689.7316				0.016
[HGe(μ-NH)]$_2$	1	C_i	0	0	−4262.9194	−4262.8752	−4262.8683	−4262.9055	0.014
**N_2Ge_2**	3	C_i		2.02	−4262.8893				0.015
	3	C_i	0	2.02	−4262.8910	−4262.8467	−4262.8399	−4262.8783	0.015
[HSn(μ-NH)]$_2$	1	C_i	0	0	−539.0102	−538.9698	−538.9622	−539.0020	0.018
**N_2Sn_2**	3	C_i		2.03	−538.9845				0.020
	3	C_i	0	2.03	−538.9861	−538.9456	−538.9381	−538.9790	0.020
[O(μ-GeH)$_2$NH]	1	C_2	0	0	−4282.7956	−4282.7627	−4282.7564	−4282.7919	0.016
**$NOGe_2$**	3	C_2		2.02	−4282.7717				0.016
	3	C_1	0	2.02	−4282.7725	−4282.7399	−4282.7334	−4282.7712	0.016
[HGe(μ-O)]$_2$	1	C_{2h}	0	0	−4302.6714	−4302.6496	−4302.6437	−4302.6786	0.015
**O_2Ge_2**	3	C_{2h}		2.02	−4302.6549				0.017
	3	C_{2h}	0	2.02	−4302.6555	−4302.6338	−4302.6278	−4302.6640	0.017
[HGe(μ-F)]$_2^{2+}$	1	C_{2h}	1	0	−4351.0824	−4351.0671	−4351.0599	−4351.0978	0.014
**$F_2Ge_2^{2+}$**	3	C_{2h}		2.04	−4351.0793				0.015
	3	C_{2h}	0	2.04	−4351.0794	−4351.0617	−4351.0547	−4351.0930	0.015
[ISi(μ-SiH$_2$)]$_2$	1	D_{2h}	—[a]	0	−1752.9119	—[a]	—[a]	—[a]	0.013
**$Si_2Si_2^I$**	3	D_{2h}		2.05	−1752.8654				0.015
	3	C_{2h}	—[a]	2.04	−1752.8959	—[a]	—[a]	—[a]	0.017
[HSi(μ-SiH$_2$)]$_2$	1	D_{2h}	1	0	−1159.7163	−1159.6645	−1159.6567	−1159.6939	0.018
**Si_2Si_2**	3	D_{2h}		2.05	−1159.6854				0.017
	3	C_{2h}	0	2.04	−1159.7031	−1159.6520	−1159.6434	−1159.6838	0.019
[P(μ-PH)]$_2$	1	C_s	0	0	−1364.7838	−1364.7616	−1364.7550	−1364.7917	0.019
**P_2P_2**	3	C_s		2.06	−1364.7281				0.022
	3	C_s	0	2.05	−1364.7616	−1364.7398	−1364.7333	−1364.7708	0.026
[c]	1	C_{2h}	1	0	−1364.7836	−1364.7616	−1364.7558	−1364.7903	0.018
	3	C_{2h}		2.06	−1364.7325				0.023
[P(μ-NH)$_2$As]	1	C_{2v}	0	0	−2685.8990	−2685.8670	−2685.8614	−2685.8952	0.018
**N_2PAs**	3	C_{2v}		2.04	−2685.8448				0.016
	3	C_s	0	2.04	−2685.8566	−2685.8249	−2685.8194	−2685.8546	0.018
[d]	3	C_{2v}		2.08	−2685.8322				0.023

[b] Optimized geometry in D_{2h} symmetry. [c] Optimized geometry in C_{2h} symmetry. [d] Arsinidene (i.e. both unpaired electrons mainly localized at As) at ground state geometry.

Table S2 continued.

Compd.	M	PG	N_{imag}	$\langle S^2 \rangle$	E_{tot}	U_0	H_{298}	G_{298}	T_1
[P(μ-NH)$_2$CH]	1	C_2	0	0	−490.0172	−489.9693	−489.9642	−489.9953	0.024
**N_2CP**	3	C_2		2.04	−489.9210				0.016
	3	C_s	0	2.03	−489.9547	−489.9076	−489.9027	−489.9351	0.019
[e]	3	C_2		2.05	−489.9367				0.024
**O_2**	3	$D_{\infty h}$	0	2.05	−150.1349	−150.1313	−150.1279	−150.1512	0.016
[f]	1	$D_{\infty h}$		1.03	−150.0870				0.013
[HC(μ-CH$_2$)]$_2$	3	C_{2h}	0	2.04	−155.5592	−155.4792	−155.4730	−155.5069	0.013
**C_2C_2** [f]	1	C_{2h}		1.03	−155.5475				0.013
[HC(μ-CF$_2$)]$_2$	1	D_{2h}	1	0	−552.164	−552.1116	−552.1044	−552.1409	0.014
**$C_2C_2^F$**	3	D_{2h}		2.03	−52.121				0.014
[HC(μ-C(OH)$_2$)]$_2$	1	D_2	—[a]	0	−456.108	—[a]	—[a]	—[a]	0.014
**$C_2C_2^{OH}$**	3	D_2		2.03	−456.078				0.015
$C_5H_6F_2$	1	C_{2v}	1	0	−393.139	−393.0424	−393.0355	−393.0723	0.015
**$C_3C_2^F$**	3	C_{2v}		2.03	−393.120				0.014
[H_2CC(H)CH$_2$]$^-$	1	C_{2v}	0	0	−117.0538	−116.9910	−116.9857	−117.0152	0.013
[g]	3	C_{2v}		2.19	−116.9662				0.053

[e] Phosphinidene (i.e. both unpaired electrons mainly localized at P) at ground state geometry. [f] UCCSD(T) using quasi-restricted orbitals (QRO).[64,65] [g] Note that this state ($^3B_2$) is not the lowest triplet state. The unpaired electrons are located in the n and π^* orbitals.

Results of the multireference calculations are summarized in Tables S3-S4. All computations utilized the coupled-cluster ground state structures, unless noted otherwise. Only vertical excitations were calculated.

Multireference calculations including dynamical correlation (i.e. NEVPT2, MRCI) were based on various sets of CASSCF reference wavefunctions. For the description of the lowest singlet state (typically the overall ground state of the molecules), a state-specific CASSCF(2,2) reference was used. To verify that the minimal (2,2) active space is sufficient, additional CASSCF computations were performed that include all formal π electrons and orbitals in the active space, i.e., typically CASSCF(6,4). To compute the biradical states (and hence the important singlet-triplet gap ($\Delta E_{ST} = E_S - E_T$), state-averaged CASSCF(2,2) reference wavefunctions were used, including all four biradical states (3 singlets, 1 triplet).[19,20,66–68] All states were weighted equally.

Table S3. Summary of multireference calculations (using the coupled-cluster geometries). All computations used the def2-TZVP basis, unless noted otherwise. Energies reported in atomic units (a.u.). Weights of the relevant determinants w_1 (…2220…), w_2 (…2202…), w_3 (…2022…), as well as LUNO occupancy (n_{LUNO}) and biradical character β[66,69] refer to the S_0 state.

		S_0	T_1	S_1	S_2	w_1	w_2	w_3	n_{LUNO}	β
**N_2P_2** (D_{2h})		^1A_g	$^3B_{1u}$	$^1B_{1u}$	^1A_g					
state-av.	CAS(2,2)	−791.5979	−791.5582	−791.4019	−791.3403	0.90	0.10	—	0.21	0.21
	MRCI	−792.2841	−792.2291	−792.1329	−792.0660	0.78	0.06	<0.01	0.17	0.14
	NEVPT2	−792.2823	−792.2246	−792.1459	−792.0841	—	—	—	—	—
state-specific	CAS(2,2)	−791.6099	—	—	—	0.88	0.12	—	0.24	0.24
	MRCI	−792.2861	—	—	—	0.77	0.07	<0.01	0.20	0.17
	NEVPT2	−792.2787	—	—	—	—	—	—	0.27	—
	CAS(6,4)	−791.6124	—	—	—	0.88	0.11	<0.01	0.23	0.23
**N_2As_2** (D_{2h})		^1A_g	$^3B_{1u}$	$^1B_{1u}$	^1A_g					
state-av.	CAS(2,2)	−4578.4896	−4578.4609	−4578.3076	−4578.2582	0.87	0.13	—	0.25	0.25
	MRCI	−4579.3728	−4579.3294	−4579.2294	−4579.1730	0.77	0.08	<0.01	0.20	0.18
	NEVPT2	−4579.4167	−4579.3687	−4579.2957	−4579.3157[a]	—	—	—	—	—
state-specific	CAS(2,2)	−4578.5003	—	—	—	0.86	0.14	—	0.29	0.29
	MRCI	−4579.3740	—	—	—	0.76	0.09	<0.01	0.23	0.22
	NEVPT2	−4579.4119	—	—	—	—	—	—	0.32	—
	CAS(6,4)	−4578.5030	—	—	—	0.86	0.13	<0.01	0.27	0.26
**N_2Sb_2** (D_{2h})		^1A_g	$^3B_{1u}$	$^1B_{1u}$	^1A_g					
state-av.	CAS(2,2)	−588.6159	−588.6007	−588.4656	−588.4343	0.83	0.17	—	0.33	0.33
	MRCI	−589.6291	−589.6023	−589.5086	−589.4702	0.74	0.11	<0.01	0.27	0.25
	NEVPT2	−589.7164	−589.6853	−589.6218	−589.6008	—	—	—	—	—
state-specific	CAS(2,2)	−588.6266	—	—	—	0.81	0.19	—	0.38	0.38
	MRCI	−589.6311	—	—	—	0.71	0.13	<0.01	0.31	0.30
	NEVPT2	−589.7117	—	—	—	—	—	—	0.40	—
	CAS(6,4)	−588.6286	—	—	—	0.82	0.17	<0.01	0.36	0.35
**N_2Bi_2** (C_{2h})		^1A_g	^3A_u	^1A_u	^1A_g					
state-av.	CAS(2,2)	−537.1744	−537.1624	−537.0570	−537.0250	0.85	0.15	—	0.29	0.29
	MRCI	−538.3150	−538.2926	−538.2172	−538.1795	0.74	0.09	<0.01	0.24	0.22
	NEVPT2	−538.4777	−538.4505	−538.4134	−538.4173	—	—	—	—	—
state-specific	CAS(2,2)	−537.1951	—	—	—	0.78	0.22	—	0.43	0.43
	MRCI	−538.3219	—	—	—	0.68	0.15	<0.01	0.37	0.36
	NEVPT2	−538.4701	—	—	—	—	—	—	0.45	—
	CAS(6,4)	−537.1971	—	—	—	0.80	0.20	<0.01	0.41	0.40

[a] The energy lowering due to the NEVPT2 transition energy correction is unusually large (> 3 eV).

Table S3 continued.

		S_0	T_1	S_1	S_2	w_1	w_2	w_3	n_{LUNO}	β
**$P_2C_2^{Cl}$** (C_i)		^1A_g	^3A_u	^1A_u	^1A_g					
state-av.	CAS(2,2)	−1677.2787	−1677.2662	−1677.1624	−1677.0877	0.92	0.08	—	0.16	0.16
	MRCI	−1678.2490	−1678.2217	−1678.1567	−1678.0871	0.75	0.05	<0.01	0.15	0.12
	NEVPT2	−1678.2812	−1678.2503	−1678.2114	−1678.1686	—	—	—	—	—
state-specific	CAS(2,2)	−1677.3189	—	—	—	0.85	0.15	—	0.31	0.31
	MRCI	−1678.2606	—	—	—	0.71	0.10	<0.01	0.26	0.24
	NEVPT2	−1678.2697	—	—	—	—	—	—	0.33	—
	CAS(6,4)	−1677.3207	—	—	—	0.85	0.15	<0.01	0.30	0.29
**P_2C_2** (C_i)		^1A_g	^3A_u	^1A_u	^1A_g					
state-av.	CAS(2,2)	−759.4358	−759.4120	−759.3212	−759.2184	0.95	0.05	—	0.09	0.09
	MRCI	−760.0869	−760.0396	−759.9893	−759.8985	0.80	0.03	<0.01	0.10	0.06
	NEVPT2	−760.0748	−760.0242	−759.9853	−759.9081	—	—	—	—	—
state-specific	CAS(2,2)	−759.4705	—	—	—	0.90	0.10	—	0.19	0.19
	MRCI	−760.0930	—	—	—	0.78	0.06	<0.01	0.16	0.13
	NEVPT2	−760.0685	—	—	—	—	—	—	0.23	—
	CAS(6,4)	−759.4746	—	—	—	0.90	0.09	0.01	0.19	0.18
**P_2B_2** (D_{2h})		^1A_g	$^3B_{2u}$	$^1B_{2u}$	^1A_g					
state-av.	CAS(2,2)	−734.2964	−734.2583	−734.1401	−734.0773	0.92	0.08	—	0.16	0.16
	MRCI	−734.8554	−734.8101	−734.7362	−734.6763	0.79	0.05	<0.01	0.13	0.12
	NEVPT2	−734.8137	−734.7670	−734.7075	−734.6727	—	—	—	—	—
state-specific	CAS(2,2)	−734.3082	—	—	—	0.90	0.10	—	0.19	0.19
	MRCI	−734.8584	—	—	—	0.78	0.06	<0.01	0.16	0.14
	NEVPT2	−734.8134	—	—	—	—	—	—	0.21	—
	CAS(6,4)	−734.3082	—	—	—	0.90	0.10	<0.01	0.19	0.19
**P_2Al_2** (D_{2h})		^1A_g	$^3B_{2u}$	$^1B_{2u}$	^1A_g					
state-av.	CAS(2,2)	−1168.7992	−1168.7576	−1168.6789	−1168.6075	0.96	0.04	—	0.09	0.09
	MRCI	−1169.3136	−1169.2623	−1169.2116	−1169.1433	0.80	0.03	<0.01	0.07	0.06
	NEVPT2	−1169.2682	−1169.2137	−1169.1720	−1169.1092	—	—	—	—	—
state-specific	CAS(2,2)	−1168.8073	—	—	—	0.95	0.05	—	0.10	0.10
	MRCI	−1169.3158	—	—	—	0.80	0.03	<0.01	0.09	0.07
	NEVPT2	−1169.2680	—	—	—	—	—	—	0.12	—
	CAS(6,4)	−1168.8073	—	—	—	0.95	0.05	<0.01	0.10	0.10

Table S3 continued.

		S_0	T_1	S_1	S_2	w_1	w_2	w_3	n_{LUNO}	β
**P_2Ga_2** (C_{2h})		^1A_g	^3B_u	^1B_u	^1A_g					
state-av.	CAS(2,2)	−4531.4392	−4531.4119	−4531.3201	−4531.2677	0.92	0.08	—	0.16	0.16
	MRCI	−4532.2028	−4532.1681	−4532.1033	−4532.0533	0.78	0.05	<0.01	0.13	0.12
	NEVPT2	−4532.2115	−4532.1741	−4532.1240	−4532.0819	—	—	—	—	—
state-specific	CAS(2,2)	−4531.4510	—	—	—	0.90	0.10	—	0.19	0.19
	MRCI	−4532.2050	—	—	—	0.77	0.07	<0.01	0.17	0.16
	NEVPT2	−4532.2097	—	—	—	—	—	—	0.21	—
	CAS(6,4)	−4531.4510	—	—	—	0.90	0.10	<0.01	0.19	0.19
**S_2N_2** (D_{2h})		^1A_g	$^3B_{2u}$	$^1B_{2u}$	^1A_g					
state-av.	CAS(2,2)	−903.9078	−903.8227	−903.6352	−903.4948	0.95	0.05	—	0.11	0.11
	MRCI	−904.6517	−904.5418	−904.4454	−904.3321	0.80	0.03	<0.01	0.11	0.07
	NEVPT2	−904.6603	−904.5422	−904.4703	−904.4360[a]	—	—	—	—	—
state-specific	CAS(2,2)	−903.9351	—	—	—	0.92	0.08	—	0.17	0.17
	MRCI	−904.6561	—	—	—	0.80	0.04	<0.01	0.14	0.10
	NEVPT2	−904.6541	—	—	—	—	—	—	0.21	—
	CAS(6,4)	−903.9387	—	—	—	0.92	0.08	0.01	0.17	0.16
**O_3** (C_{2v})		$^1A_1$	$^3B_2$	$^1B_2$	$^1A_1$					
state-av.	CAS(2,2)	−224.3910	−224.3779	−224.0695	−223.9548	0.85	0.15	—	0.29	0.29
	MRCI	−225.1133	−225.0524	−224.9209	−224.8400	0.78	0.09	<0.01	0.25	0.21
	NEVPT2	−225.1229	−225.0519	−224.9270	−224.9388[a]	—	—	—	—	—
state-specific	CAS(2,2)	−224.4440	—	—	—	0.79	0.21	—	0.42	0.42
	MRCI	−225.1157	—	—	—	0.76	0.12	<0.01	0.31	0.28
	NEVPT2	−225.0975	—	—	—	—	—	—	0.45	—
	CAS(4,3)	−224.4474	—	—	—	0.80	0.19	<0.01	0.39	0.39
**N_2C_2** (C_i)		^1A_g	^3A_u	^1A_u	^1A_g					
state-av.	CAS(2,2)	−186.9008	−186.8353	−186.6473	−186.5417	0.93	0.07	—	0.15	0.15
	MRCI	−187.6327	−187.5466	−187.4337	−187.3307	0.81	0.04	<0.01	0.13	0.10
	NEVPT2	−187.6308	−187.5430	−187.4501	−187.3615	—	—	—	—	—
state-specific	CAS(2,2)	−186.9262	—	—	—	0.90	0.10	—	0.21	0.21
	MRCI	−187.6340	—	—	—	0.80	0.06	<0.01	0.17	0.15
	NEVPT2	−187.6254	—	—	—	—	—	—	0.23	—
	CAS(6,4)	−186.9298	—	—	—	0.90	0.10	<0.01	0.20	0.19
	CAS(22,12)	−186.9301	—	—	—	0.90	0.09	<0.01	0.20	0.19

[a] The energy lowering due to the NEVPT2 transition energy correction is unusually large (> 3 eV).

Table S3 continued.

		S_0	T_1	S_1	S_2	w_1	w_2	w_3	n_{LUNO}	β
**N_2Si_2** (C_{2h})		^1A_g	^3B_u	^1B_u	^1A_g					
state-av.	CAS(2,2)	−689.1640	−689.1466	−688.9605	−688.9289	0.81	0.19	—	0.39	0.39
	MRCI	−689.8016	−689.7759	−689.6527	−689.6202	0.72	0.14	<0.01	0.34	0.32
	NEVPT2	−689.7918	−689.7667	−689.6602	−689.6424	—	—	—	—	—
state-specific	CAS(2,2)	−689.1854	—	—	—	0.76	0.24	—	0.48	0.48
	MRCI	−689.8032	—	—	—	0.69	0.17	<0.01	0.41	0.40
	NEVPT2	−689.7854	—	—	—	—	—	—	0.49	—
	CAS(6,4)	−689.1861	—	—	—	0.76	0.24	<0.01	0.48	0.47
	CAS(22,12)	−689.1861	—	—	—	0.76	0.24	<0.01	0.47	0.47
**N_2Si_2** (D_{2h})		^1A_g	$^3B_{1u}$	$^1B_{1u}$	^1A_g					
state-av.	CAS(2,2)	−689.0641	−689.0486	−688.9150	−688.8862	0.83	0.17	—	0.34	0.34
	MRCI	−689.7094	−689.6828	−689.5961	−689.5601	0.74	0.11	<0.01	0.27	0.25
	NEVPT2	−689.6996	−689.6731	−689.5989	−689.5615	—	—	—	—	—
state-specific	CAS(2,2)	−689.0752	—	—	—	0.81	0.19	—	0.37	0.37
	MRCI	−689.7103	—	—	—	0.73	0.13	<0.01	0.31	0.30
	NEVPT2	−689.6980	—	—	—	—	—	—	0.39	—
	CAS(6,4)	−689.0762	—	—	—	0.82	0.18	<0.01	0.37	0.36
**N_2Ge_2** (C_i)		^1A_g	^3A_u	^1A_u	^1A_g					
state-av.	CAS(2,2)	−4261.9691	−4261.9498	−4261.7813	−4261.7444	0.83	0.17	—	0.34	0.34
	MRCI	−4262.8388	−4262.8098	−4262.6951	−4262.6559	0.73	0.12	<0.01	0.29	0.28
	NEVPT2	−4262.8755	−4262.8450	−4262.7593	-4262.7934[a]	—	—	—	—	—
state-specific	CAS(2,2)	−4261.9871	—	—	—	0.79	0.21	—	0.42	0.42
	MRCI	−4262.8394	—	—	—	0.70	0.15	<0.01	0.36	0.35
	NEVPT2	−4262.8671	—	—	—	—	—	—	0.44	—
	CAS(6,4)	−4261.9884	—	—	—	0.80	0.20	<0.01	0.41	0.40
**N_2Sn_2** (C_i)		^1A_g	^3A_u	^1A_u	^1A_g					
state-av.	CAS(2,2)	−537.9124	−537.8977	−537.7499	−537.7185	0.82	0.18	—	0.35	0.35
	MRCI	−538.9066	−538.8825	−538.7817	−538.7470	0.72	0.12	<0.01	0.30	0.28
	NEVPT2	−538.9779	−538.9512	−538.8880	−538.9316[a]	—	—	—	—	—
state-specific	CAS(2,2)	−537.9290	—	—	—	0.78	0.22	—	0.45	0.45
	MRCI	−538.9071	—	—	—	0.69	0.16	<0.01	0.38	0.37
	NEVPT2	−538.9673	—	—	—	—	—	—	0.46	—
	CAS(6,4)	−537.9306	—	—	—	0.79	0.21	<0.01	0.42	0.42

[a] The energy lowering due to the NEVPT2 transition energy correction is unusually large (> 3 eV).

Table S3 continued.

		S_0	T_1	S_1	S_2	w_1	w_2	w_3	n_{LUNO}	β
**$NOGe_2$** (C_2)		^1A	^3B	^1B	^1A					
state-av.	CAS(2,2)	−4281.8253	−4281.8119	−4281.6387	−4281.6073	0.80	0.20	—	0.39	0.39
	MRCI	−4282.7153	−4282.6923	−4282.5738	−4282.5396	0.71	0.14	<0.01	0.34	0.32
	NEVPT2	−4282.7580	−4282.7336	−4282.6451	−4282.6503[a]	—	—	—	—	—
state-specific	CAS(2,2)	−4281.8444	—	—	—	0.76	0.24	—	0.48	0.48
	MRCI	−4282.7164	—	—	—	0.68	0.17	<0.01	0.41	0.40
	NEVPT2	−4282.7495	—	—	—	—	—	—	0.49	—
	CAS(6,4)	−4281.8457	—	—	—	0.77	0.23	<0.01	0.46	0.46
**O_2Ge_2** (C_{2h})		^1A_g	^3B_u	^1B_u	^1A_g					
state-av.	CAS(2,2)	−4301.6826	−4301.6748	−4301.4911	−4301.4689	0.76	0.24	—	0.49	0.49
	MRCI	−4302.5922	−4302.5761	−4302.4502	−4302.4243	0.68	0.17	<0.01	0.42	0.41
	NEVPT2	−4302.6406	−4302.6238	−4302.5291	−4302.5104	—	—	—	—	—
state-specific	CAS(2,2)	−4301.7014	—	—	—	0.72	0.28	—	0.56	0.56
	MRCI	−4302.5933	—	—	—	0.65	0.21	<0.01	0.49	0.48
	NEVPT2	−4302.6325	—	—	—	—	—	—	0.57	—
	CAS(6,4)	−4301.7025	—	—	—	0.73	0.27	<0.01	0.55	0.54
**$F_2Ge_2^{2+}$** (C_{2h})		^1A_g	^3B_u	^1B_u	^1A_g					
state-av.	CAS(2,2)	−4350.1178	−4350.1173	−4349.8870	−4349.8820	0.61	0.39	—	0.79	0.79
	MRCI	−4351.0188	−4351.0160	−4350.8432	−4350.8373	0.55	0.33	<0.01	0.75	0.74
	NEVPT2	−4351.0492	−4351.0465	−4350.8926	−4350.8838	—	—	—	—	—
state-specific	CAS(2,2)	−4350.1323	—	—	—	0.59	0.41	—	0.81	0.81
	MRCI	−4351.0195	—	—	—	0.54	0.34	<0.01	0.77	0.77
	NEVPT2	−4351.0432	—	—	—	—	—	—	0.81	—
	CAS(6,4)	−4350.1325	—	—	—	0.60	0.40	<0.01	0.81	0.81
**$Si_2Si_2^{I}$** (D_{2h})		^1A_g	$^3B_{3u}$	$^1B_{3u}$	^1A_g					
state-av.	CAS(2,2)	−1751.4317	−1751.3919	−1751.2799	−1751.2194	0.92	0.08	—	0.15	0.15
	MRCI	−1752.7155	−1752.6703	−1752.5880	−1752.5283	0.77	0.05	<0.01	0.13	0.12
	NEVPT2	−1752.8773	−1752.8277	−1752.7785	−1752.7257	—	—	—	—	—
state-specific	CAS(2,2)	−1751.4409	—	—	—	0.91	0.09	—	0.17	0.17
	MRCI	−1752.7181	—	—	—	0.76	0.06	<0.01	0.15	0.14
	NEVPT2	−1752.8759	—	—	—	—	—	—	0.20	—
	CAS(6,4)	−1751.4409	—	—	—	0.91	0.09	<0.01	0.17	0.17

Table S3 continued.

		S_0	T_1	S_1	S_2	w_1	w_2	w_3	n_{LUNO}	β
**Si_2Si_2** (D_{2h})		^1A_g	$^3B_{3u}$	$^1B_{3u}$	^1A_g					
state-av.	CAS(2,2)	−1159.1714	−1159.1458	−1159.0235	−1158.9782	0.89	0.11	—	0.23	0.23
	MRCI	−1159.6751	−1159.6428	−1159.5661	−1159.5235	0.75	0.07	<0.01	0.19	0.18
	NEVPT2	−1159.6263	−1159.5921	−1159.5289	−1159.4857	—	—	—	—	—
state-specific	CAS(2,2)	−1159.1807	—	—	—	0.87	0.13	—	0.25	0.25
	MRCI	−1159.6754	—	—	—	0.75	0.08	<0.01	0.22	0.20
	NEVPT2	−1159.6263	—	—	—	—	—	—	0.27	—
	CAS(6,4)	−1159.1807	—	—	—	0.87	0.13	<0.01	0.25	0.25
**P_2P_2** (C_{2h})		^1A_g	^3A_u	^1A_u	^1A_g					
state-av.	CAS(2,2)	−1364.1121	−1364.0731	−1363.9865	−1363.9034	0.95	0.05	—	0.09	0.09
	MRCI	−1364.7196	−1364.6690	−1364.6187	−1364.5472	0.79	0.03	<0.01	0.11	0.07
	NEVPT2	−1364.7059	−1364.6546	−1364.6176	−1364.5639	—	—	—	—	—
state-specific	CAS(2,2)	−1364.1331	—	—	—	0.91	0.09	—	0.18	0.18
	MRCI	−1364.7206	—	—	—	0.77	0.05	<0.01	0.16	0.13
	NEVPT2	−1364.7024	—	—	—	—	—	—	0.22	—
	CAS(6,4)	−1364.1367	—	—	—	0.91	0.08	0.01	0.17	0.15
**P_2P_2** (C_s)		$^1A'$	$^3A''$	$^1A''$	$^1A'$					
state-av.	CAS(2,2)	−1364.1130	−1364.0689	−1363.9801	−1363.8879	0.96	0.04	—	0.08	0.08
	MRCI	−1364.7200	−1364.6644	−1364.6130	−1364.5349	0.79	0.02	<0.01	0.10	0.06
	NEVPT2	−1364.7065	−1364.6495	−1364.6115	−1364.5547	—	—	—	—	—
state-specific	CAS(2,2)	−1364.1334	—	—	—	0.92	0.08	—	0.16	0.16
	MRCI	−1364.7211	—	—	—	0.77	0.05	<0.01	0.15	0.12
	NEVPT2	−1364.7034	—	—	—	—	—	—	0.21	—
	CAS(6,4)	−1364.1368	—	—	—	0.92	0.07	0.01	0.15	0.14
**N_2PAs**[b] (C_{2v})		$^1A_1$	$^3A_1$	$^1A_1$	$^1A_1$					
state-av.	CAS(2,2)	−2685.0444	−2685.0093	−2684.8731	−2684.7790	0.89	0.11	—	0.21	0.21
	MRCI	−2685.8301	−2685.7787	−2685.6950	−2685.6023	0.78	0.06	<0.01	0.17	0.15
	NEVPT2	−2685.8514	−2685.7958	−2685.7355	−2685.6451	—	—	—	—	—
state-specific	CAS(2,2)	−2685.0560	—	—	—	0.88	0.12	—	0.24	0.24
	MRCI	−2685.8319	—	—	—	0.78	0.07	<0.01	0.19	0.17
	NEVPT2	−2685.8476	—	—	—	—	—	—	0.27	—
	CAS(6,4)	−2685.0588	—	—	—	0.89	0.11	<0.01	0.22	0.22

[b] Biradical states of a formal 1,3-diyl.

Table S3 continued.

		S_0	T_1	S_1	S_2	w_1	w_2	w_3	n_{LUNO}	β
**N_2PAs**[c] (C_{2v})		$^1A_1$	$^3A_2$	$^1A_2$	$^1A_1$					
state-av.	CAS(2,3)	−2685.0462	−2684.9583	−2684.9383	−2684.8250	0.88	0.12	<0.01	<0.01[d]	<0.01[e]
	MRCI	−2685.8319	−2685.7585	−2685.7430	−2685.6346	0.78	0.07	<0.01	0.02[d]	<0.01[e]
	NEVPT2	−2685.8505	−2685.7862	−2685.7509	−2685.6752	—	—	—	—	—
state-specif.	CAS(2,3)	−2685.0564	—	—	—	0.88	0.12	<0.01	0.00	<0.01[e]
	MRCI	−2685.8323	—	—	—	0.78	0.07	<0.01	0.02	<0.01[e]
	NEVPT2	−2685.8445	—	—	—	—	—	—	0.03	—
**N_2CP** [b] (C_2)		^1A	^3A	^1A	^1A					
state-av.	CAS(2,2)	−489.2467	−489.1767	−489.0673	−488.8675	0.93	0.04	—	0.09	0.09
	MRCI	−489.9631	−489.8695	−489.8067	−489.6324	0.82	0.02	<0.01	0.09	0.05
	NEVPT2	−489.9653	−489.8661	−489.8265	−489.6639	—	—	—	—	—
state-specific	CAS(2,2)	−489.2691	—	—	—	0.96	0.04	—	0.09	0.09
	MRCI	−489.9677	—	—	—	0.82	0.03	<0.01	0.09	0.06
	NEVPT2	−489.9621	—	—	—	—	—	—	0.13	—
	CAS(6,4)	−489.2746	—	—	—	0.96	0.03	0.01	0.08	0.05
**N_2CP** [f] (C_2)		^1A	^3A	^1A	^1A					
state-av.	CAS(2,2)[g]	−489.2449	−489.1851	−489.1559	−489.0144	1.00	<0.01	—	0.01	0.01
	MRCI	−489.9574	−489.8816	−489.8604	−489.7235	0.85	0.00	<0.01	0.04	0.00
	NEVPT2	−489.9554	−489.8809	−489.8628	−489.7190	—	—	—	—	—
state-specif.	CAS(2,2)	−489.2513	—	—	—	1.00	0.00	—	0.01	0.01
	MRCI	−489.9598	—	—	—	0.85	0.00	<0.01	0.04	0.01
	NEVPT2	−489.9547	—	—	—	—	—	—	0.06	—
**$C_2P_2^{CH_2}$** (D_{2h})[h]		^1A_g	$^3B_{2u}$	$^1B_{2u}$	^1A_g					
state-av.	CAS(2,2)	−835.2069	−835.2448	−835.1464	−835.1085	0.51	0.49	—	0.98	0.98
	MRCI	−836.0925	−836.1172	−836.0574	−836.0194	0.43	0.37	<0.01	0.92	0.92
	NEVPT2	−836.1166	−836.1383	−836.1112	−836.0675	—	—	—	—	—
state-specific	CAS(2,2)	−835.2416	—	—	—	0.50	0.50	—	0.99	0.99
	MRCI	−836.1027	—	—	—	0.42	0.39	<0.01	0.96	0.97
	NEVPT2	−836.1114	—	—	—	—	—	—	0.98	—
	CAS(6,6)	−835.2950	—	—	—	0.46	0.45	<0.01	0.99	0.99

[b] Biradical states of a formal 1,3-diyl. [c] Arsinidene states. [d] n_{LUNO+1}. [e] $\beta=2w_3/(w_1+w_3)$. [f] Phosphinidene states. [g] Due to the low transannular biradical character, only a CASSCF(2,2) wavefunction with altered active space was used for description of the phosphinidene character. [h] Computations were performed using the DFT geometries.

Table S3 continued.

		S_0	T_1	S_1	S_2	w_1	w_2	w_3	n_{LUNO}	β
**$C_2P_2^{NHC1}$** (C_{2h}) [h]		^1A_g	^3B_u	^1B_u	^1A_g					
st.-av.	CAS(2,2)	−1209.3267	−1209.3220	−1209.2208	−1209.1590	0.90	0.10	—	0.20	0.20
	NEVPT2	−1211.7006	−1211.6760	−1211.6480	−1211.5945	—	—	—	—	—
state-specific	CAS(2,2)	−1209.3511	—	—	—	0.86	0.14	—	0.27	0.27
	NEVPT2	−1211.6916	—	—	—	—	—	—	0.30	—
	CAS(6,6)	−1209.3787				0.81	0.16	<0.01	0.33	0.32
	CAS(14,10)	−1209.3855	—	—	—	0.81	0.14	<0.01	0.31	0.30
**$C_2P_2^{NHC2}$** (D_{2h}) [h]		^1A_g	$^3B_{2u}$	$^1B_{2u}$	^1A_g					
st.-av.	CAS(2,2)	−1206.9705	−1206.9549	−1206.8560	−1206.7856	0.92	0.08	—	0.16	0.16
	NEVPT2	−1209.3126	−1209.2748	−1209.2467	−1209.1849	—	—	—	—	—
state-specific	CAS(2,2)	−1206.9929	—	—	—	0.89	0.11	—	0.22	0.22
	NEVPT2	−1209.3043	—	—	—	—	—	—	0.25	—
	CAS(6,6)	−1207.0175				0.85	0.12	<0.01	0.25	0.24
	CAS(18,12)	−1207.0878	—	—	—	0.79	0.08	<0.01	0.22	0.19
**O_2** ($D_{\infty h}$)		$^1\Delta_g$ [i]	$^3\Sigma_g^-$ [j]	$^1\Delta_g$ [i]	$^3\Sigma_g^+$					
state-av.	CAS(2,2)	−149.6123	−149.6590	−149.6123	−149.5655	0.50	0.50	—	1.00	1.00
	MRCI	−150.0858	−150.1223	−150.0858	−150.0621	0.45	0.45	0.02[k]	1.02	1.00
	NEVPT2	−150.0906	−150.1325	−150.0906	−150.0527	—	—	—	—	—
state-specific	CAS(2,2)	−149.6129	—	—	—	0.50	0.50	—	1.00	1.00
	MRCI	−150.0826	—	—	—	0.45	0.45	0.02[k]	1.02	1.00
	NEVPT2	−150.0906	—	—	—	—	—	—	1.01	—
	CAS(6,4)	−149.6697	—	—	—	0.46	0.46	0.08[k]	1.08	1.00
**C_2C_2** (C_{2h})		^1A_g	^3B_u [j]	^1B_u	^1A_g					
state-av.	CAS(2,2)	−154.8507	−154.8606	−154.6636	−154.6532	0.54	0.46	—	0.93	0.93
	MRCI	−155.5102	−155.5150	−155.3912	−155.3799	0.45	0.40	<0.01	0.94	0.94
	NEVPT2	−155.4859	−155.4910	−155.3910	−155.3817	—	—	—	—	—
state-specific	CAS(2,2)	−154.8768	—	—	—	0.54	0.46	—	0.92	0.92
	MRCI	−155.5141	—	—	—	0.46	0.40	<0.01	0.92	0.92
	NEVPT2	−155.4831	—	—	—	—	—	—	0.91	—
	CAS(6,4)	−154.8770	—	—	—	0.54	0.46	<0.01	0.92	0.92

[h] Computations were performed using the DFT geometries. [i] The first two singlet states are degenerate. [j] Triplet ground state. [k] Sum of the weights of two symmetry-equivalent determinants.

Table S3 continued.

		S_0	T_1	S_2	S_3	w_1	w_2	w_3	n_{LUNO}	β
**$C_2C_2^F$** (D_{2h})		^1A_g	$^3B_{1u}$	$^1B_{1u}$	^1A_g					
state-av.	CAS(2,2)	−550.4981	−550.4605	−550.2334	−550.1759	0.85	0.15	—	0.29	0.29
	MRCI	−551.9968	−551.9527	−551.7900	−551.7326	0.73	0.10	<0.01	0.25	0.25
	NEVPT2	−552.0868	−552.0398	−551.9243	−551.8696	—	—	—	—	—
state-specific	CAS(2,2)	−550.5130	—	—	—	0.83	0.17	—	0.33	0.33
	MRCI	−552.0022	—	—	—	0.71	0.12	<0.01	0.28	0.28
	NEVPT2	−552.0848	—	—	—	—	—	—	0.34	—
	CAS(6,4)	−550.5130	—	—	—	0.83	0.17	0.00	0.33	0.33
**$C_2C_2^{OH}$** (D_2)		^1A	$^3B_1$	$^1B_1$	^1A					
state-av.	CAS(2,2)	−454.4551	−454.4295	−454.2054	−454.1635	0.82	0.18	—	0.36	0.36
	MRCI	−455.9151	−455.8837	−455.7238	−455.6810	0.69	0.12	<0.01	0.32	0.31
	NEVPT2	−456.0166	−455.9829	−455.8759	−455.8316	—	—	—	—	—
state-specific	CAS(2,2)	−454.4720	—	—	—	0.80	0.20	—	0.41	0.41
	MRCI	−455.9213	—	—	—	0.67	0.14	<0.01	0.35	0.35
	NEVPT2	−456.0139	—	—	—	—	—	—	0.41	—
	CAS(6,4)	−454.4721	—	—	—	0.80	0.20	0.00	0.41	0.41
**$C_3C_2^F$** (C_{2v})		$^1A_1$	$^3B_2$	$^1B_2$	$^1A_1$					
state-av.	CAS(2,2)	−391.7690	−391.7528	−391.5248	−391.4956	0.78	0.22	—	0.45	0.45
	MRCI	−393.0054	−392.9845	−392.8245	−392.7960	0.66	0.16	<0.01	0.40	0.40
	NEVPT2	−393.0513	−393.0291	−392.9138	−392.8873	—	—	—	—	—
state-specific	CAS(2,2)	−391.7861	—	—	—	0.74	0.26	—	0.51	0.51
	MRCI	−393.0109	—	—	—	0.64	0.19	<0.01	0.45	0.45
	NEVPT2	−393.0476	—	—	—	—	—	—	0.52	—
	CAS(8,5)	−391.7862	—	—	—	0.74	0.26	0.00	0.51	0.51
**Allyl$^-$**[k] (C_{2v})		$^1A_1$	$^3B_2$	$^1B_2$	$^1A_1$					
state-av.	CAS(2,2)	−116.4468	−116.3976	−116.3833	−116.1959	1.00	<0.01	—	<0.01	<0.01
	MRCI	−117.0164	−116.9351	−116.9303	−116.7483	0.86	<0.01	<0.01	0.03	<0.01
	NEVPT2	−117.0062	−116.9170	−116.9165	−116.7194	—	—	—	—	—
state-specific	CAS(2,2)	−116.4805	—	—	—	0.98	0.02	—	0.05	0.05
	MRCI	−117.0234	—	—	—	0.85	0.02	<0.01	0.06	0.04
	NEVPT2	−117.0068	—	—	—	—	—	—	0.08	—
	CAS(6,4)	−116.4850	—	—	—	0.98	0.02	0.01	0.05	0.04

[k] These calculations applied the aug-cc-pVTZ[59] basis set and aug-cc-pVTZ/C[70] correlation fitting basis set to improve the description of the anionic charge.

The ST gaps computed with MRCI, NEVPT2 and CCSD(T) agree reasonably well (mean signed error, MSE, and mean absolute error, MAE: CCSD(T) vs. MRCI MSE = −1.1, MAE = 6.6 kJ/mol, CCSD(T) vs. NEVPT2 MSE = 4.6, MAE = 6.7 kJ/mol, NEVPT2 vs. MRCI MSE = −5.8, MAE = 8.4 kJ/mol; see also Table S5). Note, however, that the vertical excitation energies $S_0 \rightarrow S_1$ and $S_0 \rightarrow S_2$ can differ quite significantly between MRCI and NEVPT2 calculations. It is likely that larger basis sets (with diffuse functions) and/or larger active spaces are needed for an improved description of those states. Since they were not of interest in our current study, this matter was not further investigated, though.

The state-specific CASSCF computations including all formal π-type orbitals were used to obtain a set of localized orbitals for the lowest-energy singlet state of each species. The localized orbitals were used to derive Lewis-type resonance structures of the formal π-bonding system (or the radical electrons and through-bond interactions) and determine the bond order between the formal radical sites (Table S4).

Table S4. Summary of orbital localization[71,72] of the lowest-energy singlet state (w_{11} = weight of the "biradical" or "covalent" resonance structure; BO = bond order between the formal radical sites [off-diagonal density matrix element]; *e* = number of electrons at each formal radical site [diagonal density matrix element]). All computations used the def2-TZVP basis, unless noted otherwise.

Compound	PG	Method	w_{11}	BO	*e*
**N_2P_2**	D_{2h}	CASSCF(6,4)	0.59	−0.60	1.18
**N_2As_2**	D_{2h}	CASSCF(6,4)	0.62	−0.57	1.17
**N_2Sb_2**	D_{2h}	CASSCF(6,4)	0.70	−0.52	1.13
**N_2Bi_2**	C_{2h}	CASSCF(6,4)	0.71	−0.47	1.13
**$P_2C_2^{Cl}$**	C_i	CASSCF(6,4)	0.59	−0.51	1.20[a]
**P_2C_2**	C_i	CASSCF(6,4)	0.36	−0.45	1.38
**P_2B_2**	D_{2h}	CASSCF(6,4)	0.78	+0.81	1.00
**P_2Al_2**	D_{2h}	CASSCF(6,4)	0.72	+0.90	1.00
**P_2Ga_2**	C_{2h}	CASSCF(6,4)	0.79	+0.81	1.00[a]
**S_2N_2**	D_{2h}	CASSCF(6,4)	0.33	−0.45	1.40
**O_3**	C_{2v}	CASSCF(4,3)	0.61	−0.42	1.19
**N_2C_2**	C_i	CASSCF(6,4)	0.46	−0.53	1.28[a]
**N_2C_2**	C_i	CASSCF(22,12)	0.40	−0.49	1.34
**N_2Si_2**	C_{2h}	CASSCF(6,4)	0.87	−0.49	1.03[a]
**N_2Si_2**	C_{2h}	CASSCF(22,12)	0.69	−0.40	1.15
**N_2Si_2**	D_{2h}	CASSCF(6,4)	0.76	−0.55	1.09
**N_2Ge_2**	C_i	CASSCF(6,4)	0.76	−0.50	1.10[a]
**N_2Sn_2**	C_i	CASSCF(6,4)	0.74	−0.47	1.11[a]
**$NOGe_2$**	C_2	CASSCF(6,4)	0.80	−0.46	1.08[a]
**O_2Ge_2**	C_{2h}	CASSCF(6,4)	0.84	−0.39	1.07[a]
**$F_2Ge_2^{2+}$**	C_{2h}	CASSCF(6,4)	0.96	−0.17	1.02[a]
**$Si_2Si_2^{I}$**	D_{2h}	CASSCF(6,4)	0.78	+0.83	1.00
**Si_2Si_2**	D_{2h}	CASSCF(6,4)	0.82	+0.75	1.00
**P_2P_2**	C_{2h}	CASSCF(6,4)	0.38	−0.50	1.35
**P_2P_2**	C_s	CASSCF(6,4)	0.37	−0.52	1.35
**N_2PAs**	C_{2v}	CASSCF(6,4)	0.57	−0.60	1.10 (P), 1.26 (As)
**N_2CP**	C_2	CASSCF(6,4)	0.24	−0.52	1.66 (P), 1.03 (C)
**$C_2P_2^{CH2}$**	D_{2h}	CASSCF(6,6)	0.79[b]	−0.01	0.98
**$C_2P_2^{NHC1}$**	C_{2h}	CASSCF(6,6)	0.59[b]	−0.50	1.18
**$C_2P_2^{NHC1}$**	C_{2h}	CASSCF(14,10)	—[c]	−0.51	1.20
**$C_2P_2^{NHC2}$**	D_{2h}	CASSCF(6,6)	0.51[b]	−0.52	1.24
**$C_2P_2^{NHC2}$**	D_{2h}	CASSCF(18,12)	—[c]	−0.54	1.27
**O_2**	$D_{\infty h}$	CASSCF(2,2)	1.00	0.00[d]	1.00

[a] The radical electrons are partly delocalized into the E-H (E-X) bond. [b] Due to delocalization in the backbone, there are several "diradical" resonance structures; the sum of weights of these structures is reported. [c] Not evaluated due to extensive delocalization in the backbone.

Table S4 continued.

Compound	PG	Method	w_{11}	BO	e
**O_2**	$D_{\infty h}$	CASSCF(6,4)	0.77[e,f]	+0.84[e,f]	1.50[e]
**C_2C_2**	C_{2h}	CASSCF(6,4)	0.90	+0.13	1.05
**$C_2C_2^F$**	D_{2h}	CASSCF(6,4)	0.86	+0.67	1.00
**$C_2C_2^{OH}$**	D_2	CASSCF(6,4)	0.88	+0.60	1.01
**$C_3C_2^F$**	C_{2v}	CASSCF(8,5)	0.85	+0.47	1.05
**Allyl$^-$**[g]	C_{2v}	CASSCF(4,3)	0.15	−0.45	1.52

[d] This is *not* the bond order between the O atoms, since the two radical electrons are delocalized. [e] The localization includes the π_x, π_x*, π_y, and π_y* orbitals. [f] Summed over two symmetry-equivalent determinants. [g] aug-cc-pVTZ[59] basis set and aug-cc-pVTZ/C[70] correlation fitting basis set.

The descriptors of biradical character identified by us are summarized in Table S5. The results obtained from DFT (PBE-D3/def2-TZVP), coupled-cluster (CCSD(T)/def2-TZVP), multireference CI (MRCI+Q/def2-TZVP), multireference perturbation theory (NEVPT2/def2-TZVP), and CASSCF computations agree reasonably well.

Table S5. Summary of the descriptors of biradical character: Vertical (ΔE_{ST}^{v}) and adiabatic (ΔE_{ST}^{a}) singlet-triplet energy gap in kJ/mol, biradical character $\beta = 2c_2^2$ [66,69] from CASSCF(2,2) calculations, LUNO occupancy n_{LUNO}, and bond order (BO).

	DFT			CCSD(T)			MRCI		NEVPT2		CASSCF	
	PG	ΔE_{ST}^{v}	ΔE_{ST}^{a}	PG	ΔE_{ST}^{v}	ΔE_{ST}^{a}	ΔE_{ST}^{v}	n_{LUNO}	ΔE_{ST}^{v}	n_{LUNO}	β	BO
**N_2P_2**	D_{2h}	−157.9	−126.0	D_{2h}	−148.7	−122.9	−144.6	0.20	−151.5	0.27	0.24	−0.60
**N_2As_2**	C_{2h}	−129.3	−94.7	D_{2h}	−122.8	−91.3	−114.0	0.23	−126.1	0.32	0.29	−0.57
**N_2Sb_2**	C_{2h}	−84.1	−64.5	D_{2h}	−80.1	−62.0	−70.2	0.31	−81.8	0.40	0.38	−0.52
**N_2Bi_2**	C_{2h}	−70.1	−48.9	C_{2h}	−64.1	−43.3	−58.9	0.37	−71.4	0.45	0.43	−0.47
**$P_2C_2^{Cl}$**	C_i	−86.6	−13.4	C_i	−83.7	−4.6	−71.7	0.26	−81.0	0.33	0.31	−0.51
**P_2C_2**	C_i	−136.8	−59.2	C_i	−134.2	−49.9	−124.1	0.16	−132.9	0.23	0.19	−0.45
**P_2B_2**	D_{2h}	−104.0	−84.4	D_{2h}	−113.7	−96.1	−119.0	0.16	−122.7	0.21	0.19	+0.81
**P_2Al_2**	D_{2h}	−127.1	−31.7	D_{2h}	−136.9	−25.8	−134.9	0.09	−143.2	0.12	0.10	+0.90
**P_2Ga_2**	C_{2h}	−91.2	−21.3	C_{2h}	−93.1	−14.5	−91.0	0.17	−98.2	0.21	0.19	+0.81
**S_2N_2**	D_{2h}	−300.4	−240.1	D_{2h}	−295.2	−243.6	−288.6	0.14	−310.0	0.21	0.17	−0.45
**O_3**	C_{2v}	−184.4	−136.4	C_{2v}	−155.6	−115.8	−160.0	0.31	−186.5	0.45	0.42	−0.42
**N_2C_2**	C_i	−238.4	−188.6	C_i	−228.1	−175.6	−226.2	0.17	−230.7	0.23	0.21	−0.53
**N_2Si_2**	C_{2h}	−72.6	−69.7	C_{2h}	−67.1	−66.0	−67.6	0.41	−65.9	0.49	0.48	−0.49
**N_2Si_2**	D_{2h}	−67.5	148.2	D_{2h}	−74.0	164.0	−69.7	0.31	−69.7	0.39	0.37	−0.55
**N_2Ge_2**	C_i	−96.7	−90.0	C_i	−79.0	−74.7	−76.2	0.36	−80.0	0.44	0.42	−0.50

Table S5 continued.

	DFT			CCSD(T)			MRCI		NEVPT2		CASSCF	
	PG	ΔE_{ST}^{v}	ΔE_{ST}^{a}	PG	ΔE_{ST}^{v}	ΔE_{ST}^{a}	ΔE_{ST}^{v}	n_{LUNO}	ΔE_{ST}^{v}	n_{LUNO}	β	BO
**N_2Sn_2**	C_i	−81.8	−74.7	C_i	−67.5	−63.3	−63.3	0.38	−70.0	0.46	0.45	−0.47
**$NOGe_2$**	C_2	−80.5	−74.7	C_2	−62.9	−60.6	−60.4	0.41	−63.9	0.49	0.48	−0.46
**O_2Ge_2**	C_{2h}	−59.1	−56.0	C_{2h}	−43.2	−41.6	−42.5	0.49	−44.0	0.57	0.56	−0.39
**$F_2Ge_2^{2+}$**	C_{2h}	−15.5	−14.9	C_{2h}	−8.2	−8.0	−7.3	0.77	−7.0	0.81	0.81	−0.17
**$Si_2Si_2^{I}$**	D_{2h}	−118.4	−31.5	D_{2h}	−122.2	−42.1	−118.7	0.15	−130.3	0.20	0.17	+0.83
**Si_2Si_2**	D_{2h}	−77.0	−26.2	D_{2h}	−81.0	−34.6	−84.7	0.22	−89.8	0.27	0.25	+0.75
**P_2P_2**	C_{2h}	−133.6	−63.4	C_{2h}	−134.3	−57.9	−132.8	0.16	−134.9	0.22	0.18	−0.50
**P_2P_2**	C_s	—[a]	—[a]	C_s	−146.1	−58.4	−146.2	0.15	−149.6	0.21	0.16	−0.52
**N_2PAs**[b]	C_2	−149.7	−115.4	C_{2v}	−142.5	−111.6	−134.9	0.19	−145.9	0.27	0.24	−0.60
**N_2PAs**[c]	C_2	−157.5	−85.9	C_{2v}	−175.6	—	−192.7	0.02	−168.9	0.03	0.00	*n/a*
**N_2CP** [b]	C_2	−220.4	−169.4	C_2	−252.8	−164.1	−245.6	0.09	−260.5	0.13	0.09	−0.52
**N_2CP** [d]	C_2	−198.8	−52.2	C_2	−211.4	—	−199.1	0.04	−195.5	0.06	0.01	*n/a*
**$C_2P_2^{CH2}$**	D_{2h}	+41.9	+40.8	—	—	—	+64.6	0.96	+56.8	0.98	0.99	−0.01
**$C_2P_2^{NHC1}$**	C_{2h}	−61.8	−47.6	—	—	—	—	—	−64.6	0.30	0.27	−0.50
**$C_2P_2^{NHC2}$**	D_{2h}	−91.3	−80.6	—	—	—	—	—	−99.2	0.25	0.22	−0.52
**O_2**	$D_{\infty h}$	+74.0	—	$D_{\infty h}$	+125.7	—	+95.7	1.02	+109.9	1.01	1.00	0.00
**C_2C_2**	D_{2h}	+13.3	—	C_{2h}	+30.7	—	+12.6	0.92	+13.3	0.91	0.92	+0.13
**$C_2C_2^{F}$**	D_{2h}	−114.3	−86.0	D_{2h}	−114.3	—	−115.7	0.28	−123.5	0.34	0.33	+0.67
**$C_2C_2^{OH}$**	D_2	−84.6	−51.6	D_2	−79.8	—	−82.3	0.35	−88.5	0.41	0.41	+0.60
**$C_3C_2^{F}$**	C_{2v}	−70.9	−58.7	C_{2v}	−48.4	—	−55.0	0.45	−58.4	0.52	0.51	+0.47
**Allyl^{-}**	C_{2v}	−199.6	−194.1	C_{2v}	−230.1	—	−213.3	0.06	−234.2	0.08	0.05	−0.45

[a] Not a stationary point on the PES at this level of theory. [b] 1,3-diyl. [c] Arsinidene. [d] Phosphinidene.

DFT singlet-triplet gaps obtained with the aid of unrestricted (broken-symmetry) singlet wavefunctions were calculated using the formula[73]

$$\Delta E_{\mathrm{ST}} = \frac{\langle S^2 \rangle_{\mathrm{T}}}{\langle S^2 \rangle_{\mathrm{T}} - \langle S^2 \rangle_{\mathrm{BS}}} \cdot (E_{\mathrm{BS}} - E_{\mathrm{T}})$$

where $\langle S^2 \rangle_{\mathrm{T}}$ and $\langle S^2 \rangle_{\mathrm{BS}}$ are the expectation values of the S^2 operator for the triplet and broken-symmetry singlet wavefunction, respectively, and E_{T} as well as E_{BS} are the corresponding Kohn-Sham SCF energies.

3 Optimized structures

Table S6 shows all optimized structures at the DFT and coupled-cluster levels of theory. Table S7 lists the relevant structural data.

Table S6. Graphical representation of the optimized structures at the PBE-D3/def2-TZVP and CCSD(T)/def2-TZVP levels of theory (allyl anion at PBE-D3/aug-cc-pVTZ and CCSD(T)/aug-cc-pVTZ).

	singlet state		triplet state	
	DFT	CCSD(T)	DFT	CCSD(T)
**N_2P_2**	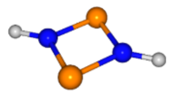	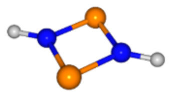	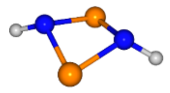	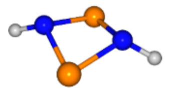
**N_2As_2**	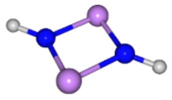	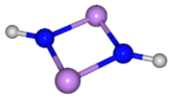	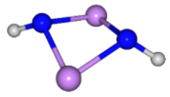	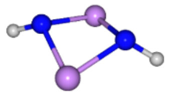
**N_2Sb_2**	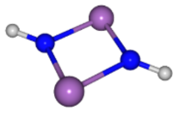	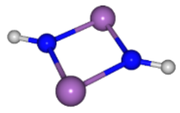	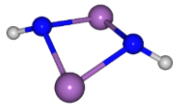	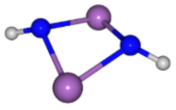
**N_2Bi_2**	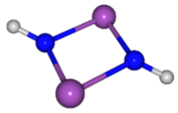	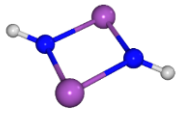	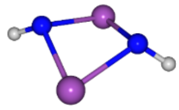	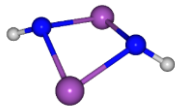
**$P_2C_2^{Cl}$**	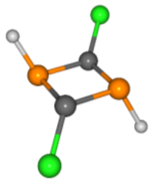	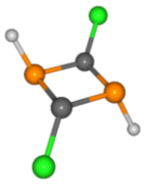	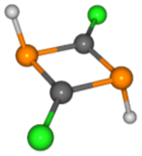	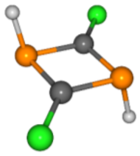
**P_2C_2**	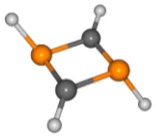	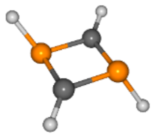	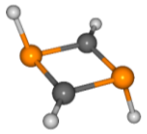	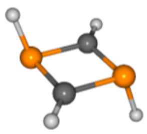

Table S6 continued.

	singlet state		triplet state	
	DFT	CCSD(T)	DFT	CCSD(T)
**P_2B_2**	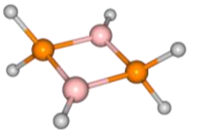	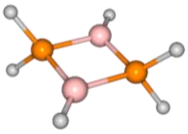	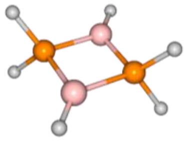	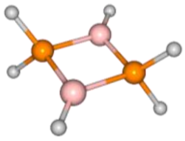
**P_2Al_2**	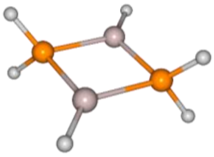	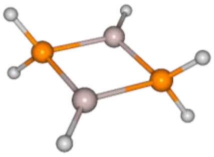	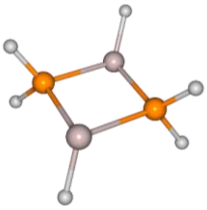	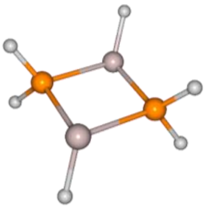
**P_2Ga_2**	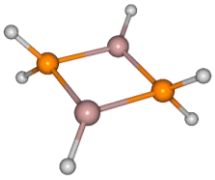	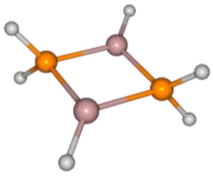	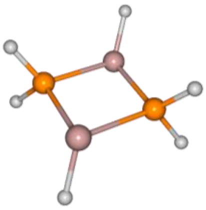	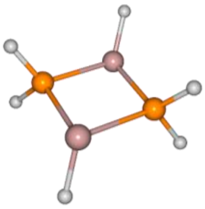
**S_2N_2**	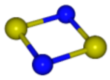	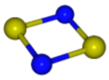	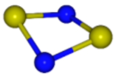	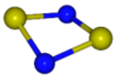
**O_3**	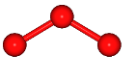	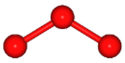	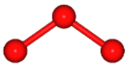	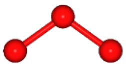
**N_2C_2**	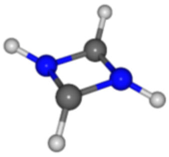	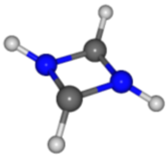	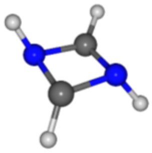	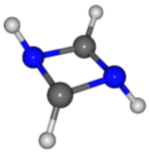
**N_2Si_2**	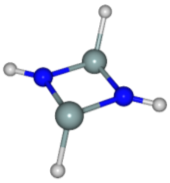	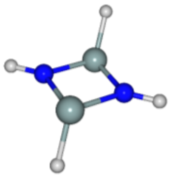	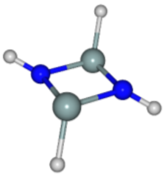	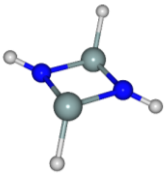
**N_2Si_2** (D_{2h})	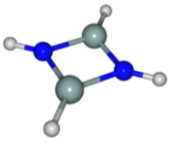	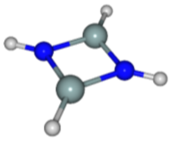	–	–

Table S6 continued.

	singlet state		triplet state	
	DFT	CCSD(T)	DFT	CCSD(T)
**N_2Ge_2**	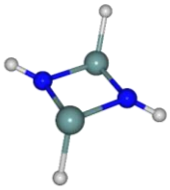	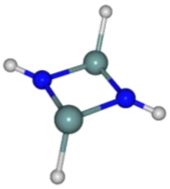	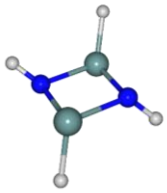	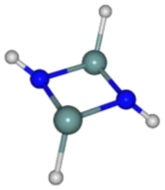
**N_2Sn_2**	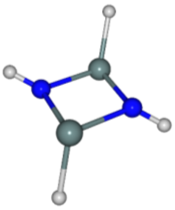	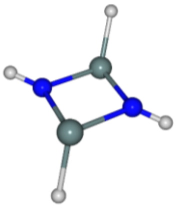	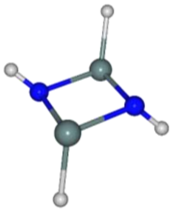	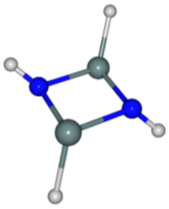
**$NOGe_2$**	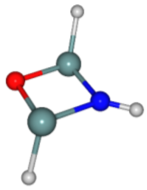	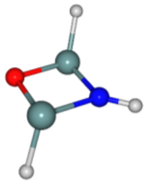	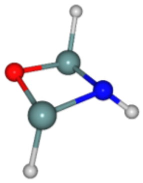	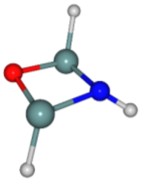
**O_2Ge_2**	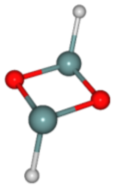	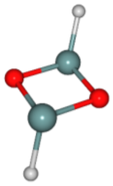	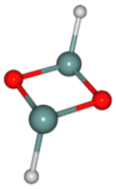	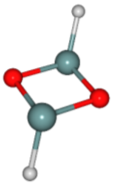
**$F_2Ge_2^{2+}$**	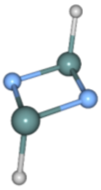	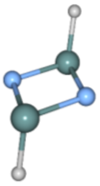	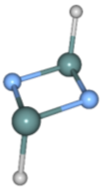	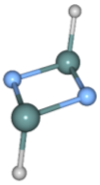
**$Si_2Si_2^I$**	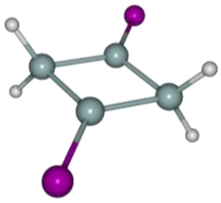	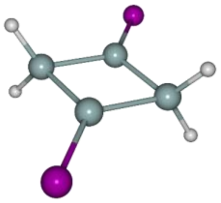	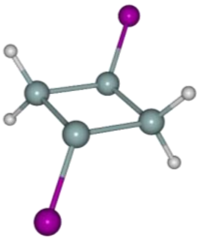	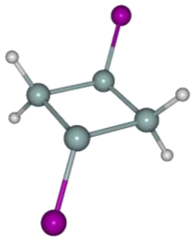
**Si_2Si_2**	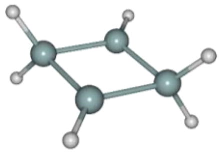	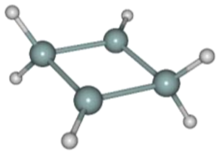	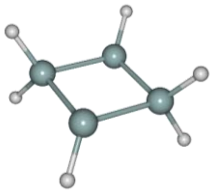	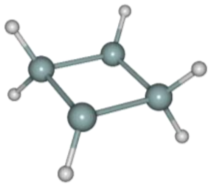

Table S6 continued.

	singlet state		triplet state	
	DFT	CCSD(T)	DFT	CCSD(T)
**P_2P_2** (C_{2h})	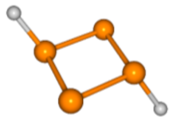	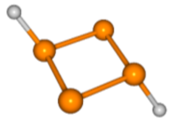	–	–
**P_2P_2** (C_s)	–	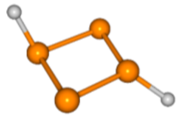	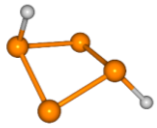	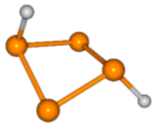
**N_2PAs**	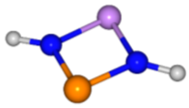	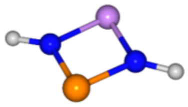	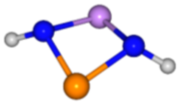	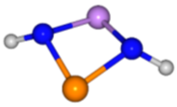
**N_2CP**	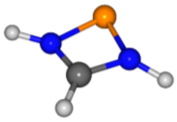	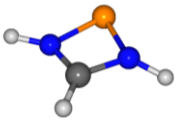	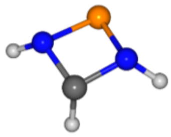	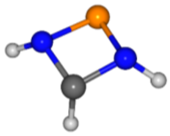
**$C_2P_2^{CH2}$**	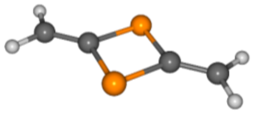	–	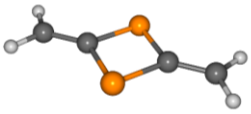	–
**$C_2P_2^{NHC1}$**	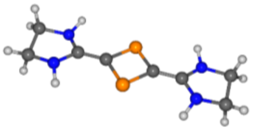	–	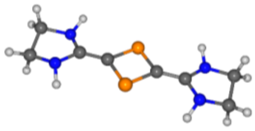	–
**$C_2P_2^{NHC2}$**	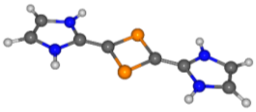	–	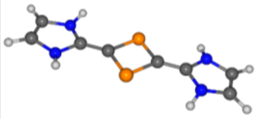	–
**O_2**	–	–	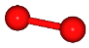	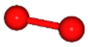

Table S6 continued.

	singlet state		triplet state	
	DFT	CCSD(T)	DFT	CCSD(T)
**C_2C_2**	–	–	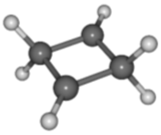	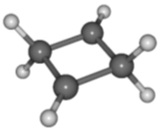
**$C_2C_2^F$**	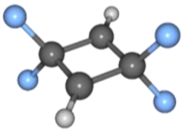	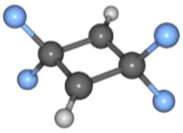	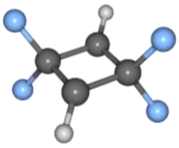	–
**$C_2C_2^{OH}$**	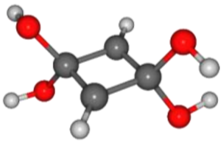	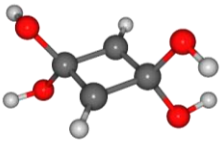	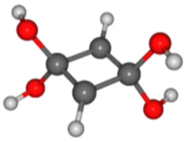	–
**$C_3C_2^F$**	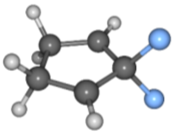	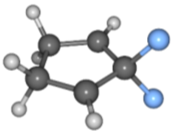	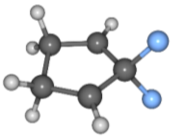	–
**Allyl$^-$**	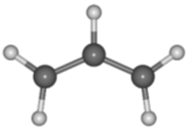	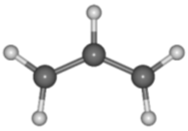	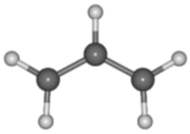	–

Table S7. Selected bond lengths and (dihedral) angles of the optimized structures at the PBE-D3/def2-TZVP and CCSD(T)/def2-TZVP levels of theory (allyl anion at PBE-D3/aug-cc-pVTZ and CCSD(T)/aug-cc-pVTZ).

		singlet state		triplet state	
	Parameter	DFT	CCSD(T)	DFT	CCSD(T)
**N_2P_2**	N–P	1.7137	1.7078	1.7625	1.7507
	N⋯N′	2.1914	2.1833	2.2983	2.2748
	P⋯P′	2.6354	2.6266	2.5215	2.5340
	P–N–P′	100.5099	100.5334	91.3421	92.7219
	N–P–N′	79.4901	79.4666	81.3872	81.0326
	∑(∡N)	360.0000	360.0000	328.4874	332.1750
	N–P–P′–N′	180.0000	180.0000	137.8399	140.5718
**N_2As_2**	N–As	1.8500	1.8342	1.9059	1.8840
	N⋯N	2.3276	2.3038	2.4445	2.4126
	As⋯As′	2.8761	2.8548	2.7469	2.7402
	As–N–As′	102.0350	102.1921	92.2138	93.3075
	N–As–N′	77.9650	77.8079	79.7809	79.6243
	∑(∡N)	357.5009	360.0000	324.2593	328.0879
	N–As–As′–N′	180.0000	180.0000	135.3390	137.7482
**N_2Sb_2**	N–Sb	2.0399	2.0178	2.0893	2.0594
	N⋯N′	2.5142	2.4828	2.6193	2.5753
	Sb⋯Sb′	3.2132	3.1816	3.0803	3.0712
	Sb–N–Sb′	103.9162	104.0655	94.9805	96.4316
	N–Sb–N′	76.0838	75.9345	77.6343	77.4026
	∑(∡N)	357.6603	360.0000	328.9370	334.1198
	N–Sb–Sb′–N′	180.0000	180.0000	136.1471	139.5590
**N_2Bi_2**	N–Bi	2.1425	2.1229	2.1954	2.1707
	N⋯N′	2.6298	2.6000	2.7411	2.6976
	Bi⋯Bi′	3.3831	3.3566	3.2331	3.2215
	Bi–N–Bi′	104.2828	104.4766	94.8402	95.8155
	N–Bi–N′	75.7172	75.5234	77.2588	76.8339
	∑(∡N)	353.5401	355.3990	323.8615	327.4034
	N–Bi–Bi′–N′	180.0000	180.0000	134.6331	135.9380

Table S7 continued.

	Parameter	singlet state DFT	singlet state CCSD(T)	triplet state DFT	triplet state CCSD(T)
**$P_2C_2^{Cl}$**	P–C	1.7702	1.7709	1.8090	1.8153
	P–C′	1.7892	1.7938	1.8090	1.8153
	P⋯P′	2.5316	2.5458	2.7871	2.8066
	C⋯C′	2.5021	2.4953	2.3069	2.3033
	C–P–C′	89.3288	88.8528	79.2290	78.7495
	P–C–P′	90.6712	91.1472	100.7710	101.2505
	∑(∡P)	313.2694	312.9536	286.6385	284.1636
	∑(∡C)	342.8632	342.5042	360.0000	360.0000
	N–P–P′–N′	180.0000	180.0000	180.0000	180.0000
**P_2C_2**	P–C	1.7452	1.7465	1.7991	1.8056
	P–C′	1.7517	1.7552	1.7991	1.8056
	P⋯P′	2.4313	2.4432	2.7028	2.7231
	C⋯C′	2.5134	2.5086	2.3752	2.3718
	C–P–C′	91.9020	91.5121	82.6188	82.1115
	P–C–P′	88.0980	88.4879	97.3812	97.8885
	∑(∡P)	321.8302	322.1857	294.1686	292.0556
	∑(∡C)	351.4738	351.5135	360.0000	360.0000
	N–P–P′–N′	180.0000	180.0000	180.0000	180.0000
**P_2B_2**	P–B	1.8810	1.8856	1.9141	1.9198
	P⋯P′	2.7560	2.7704	2.6594	2.6939
	B⋯B′	2.5607	2.5587	2.7537	2.7359
	B–P–B′	85.7921	85.4489	91.9944	90.8856
	P–B–P′	94.2079	94.5511	88.0056	89.1144
	∑(∡P)	659.2501	659.1042	658.5322	658.3945
	∑(∡B)	360.0000	360.0000	359.2556	359.2457
	P–B–B′–P′	180.0000	180.0000	180.0000	180.0000
**P_2Al_2**	P–Al	2.2996	2.3054	2.4201	2.4411
	P⋯P′	3.4924	3.5016	3.2852	3.3257
	Al⋯Al′	2.9926	2.9995	3.5545	3.5742
	Al–P–Al′	81.1858	81.1670	94.5082	94.1254
	P–Al–P′	98.8142	98.8330	85.4918	85.8746
	∑(∡P)	659.0795	658.8493	658.4200	658.1874
	∑(∡Al)	360.0000	360.0000	318.1163	315.0093
	P–Al–Al′–P′	180.0000	180.0000	180.0000	180.0000

Table S7 continued.

	Parameter	singlet state DFT	singlet state CCSD(T)	triplet state DFT	triplet state CCSD(T)
**P_2Ga_2**	P–Ga	2.3165	2.3088	2.4322	2.4286
	P···P′	3.3984	3.4111	3.2640	3.2870
	Ga···Ga′	3.1488	3.1123	3.6068	3.5761
	Ga–P–Ga′	85.6335	84.7549	95.7112	94.8232
	P–Ga–P′	94.3665	95.2451	84.2888	85.1768
	∑(∡P)	660.7476	659.7066	658.4021	658.1910
	∑(∡Ga)	355.9505	357.0917	316.4874	315.7131
	P–Ga–Ga′–P′	180.0000	180.0000	180.0000	180.0000
**S_2N_2**	S–N	1.6621	1.6553	1.7373	1.7339
	S···S′	2.3199	2.3190	2.5190	2.5072
	N···N′	2.3808	2.3626	2.2725	2.2999
	N–S–N′	91.4860	91.0672	81.6959	83.0939
	S–N–S′	88.5140	88.9328	92.9346	92.6047
	S–N–N′–S′	180.0000	180.0000	146.8318	150.0500
**O_3**	O–O′	1.2736	1.2743	1.3685	1.3584
	O′···O″	2.1858	2.1739	2.2372	2.2051
	O′–O–O″	118.2112	117.0722	109.6480	108.5180
**N_2C_2**	N–C	1.4028	1.4037	1.4536	1.4549
	N–C′	1.3969	1.3959	1.4497	1.4488
	N′–C	1.3969	1.3959	1.4365	1.4415
	N′–C′	1.4028	1.4037	1.4577	1.4594
	N···N′	1.9571	1.9592	2.1281	2.1274
	C···C′	2.0020	1.9998	1.9576	1.9680
	C–N–C′	91.2978	91.1744	84.7927	85.3391
	C–N′–C′	91.2978	91.1744	85.1197	85.4377
	N–C–N′	88.7022	88.8256	94.8369	94.5307
	N–C′–N′	88.7022	88.8256	94.1016	94.0225
	∑(∡N)	349.7497	349.6479	318.2864	317.9303
	∑(∡N′)	349.7497	349.6479	316.8111	315.8173
	∑(∡C)	338.6032	338.5849	349.2191	346.3270
	∑(∡C′)	338.6032	338.5849	341.5303	341.1921
	N–C–C′–N′	180.0000	180.0000	169.0002	171.5750

Table S7 continued.

	Parameter	singlet state DFT	singlet state CCSD(T)	triplet state DFT	triplet state CCSD(T)
**N_2Si_2**	N–Si	1.7516	1.7439	1.7591	1.7497
	N–Si′	1.7516	1.7439	1.7592	1.7502
	N⋯N′	2.3783	2.3702	2.4265	2.4004
	Si⋯Si′	2.5723	2.5587	2.5476	2.5470
	Si–N–Si′	94.4873	94.3813	92.7887	93.3926
	N–Si–N′	85.5127	85.6187	87.2113	86.6074
	∑(∡N)	360.0000	360.0000	351.2543	354.6978
	∑(∡Si)	312.2501	315.4621	316.6554	317.6634
	N–Si–Si′–N′	180.0000	180.0000	180.0000	180.0000
**N_2Si_2** (D_{2h})	N–Si	1.7377	1.7349	—	—
	N⋯N′	2.3828	2.3771	—	—
	Si⋯Si′	2.5300	2.5277	—	—
	Si–N–Si′	93.4320	93.5169	—	—
	N–Si–N′	86.5680	86.4831	—	—
	∑(∡N)	360.0000	360.0000	—	—
	∑(∡Si)	360.0000	360.0000	—	—
	N–Si–Si′–N′	180.0000	180.0000	—	—
**N_2Ge_2**	N–Ge	1.8764	1.8564	1.8884	1.8672
	N–Ge′	1.8691	1.8517	1.8928	1.8672
	N⋯N′	2.4876	2.4693	2.5938	2.5521
	Ge⋯Ge′	2.8002	2.7663	2.7513	2.7262
	Ge–N–Ge′	96.7680	96.4933	93.3759	93.7801
	N–Ge–N′	83.2320	83.5067	86.6241	86.2199
	∑(∡N)	354.0864	356.3281	339.2957	343.3592
	∑(∡Ge)	306.5192	310.4373	313.7539	315.4695
	N–Ge–Ge′–N′	180.0000	180.0000	180.0000	180.0000
**N_2Sn_2**	N–Sn	2.0740	2.0459	2.0832	2.0548
	N–Sn′	2.0675	2.0422	2.1052	2.0548
	N⋯N′	2.6808	2.6495	2.8092	2.7395
	Sn⋯Sn′	3.1568	3.1133	3.1067	3.0633
	Sn–N–Sn′	99.3215	99.2039	95.7588	96.3880
	N–Sn–N′	80.6785	80.7961	84.2412	83.6120
	∑(∡N)	353.4042	356.1385	337.5580	343.3180
	∑(∡Sn)	302.7351	305.7442	308.1531	310.8559
	N–Sn–Sn′–N′	180.0000	180.0000	180.0000	180.0000

Table S7 continued.

	Parameter	singlet state DFT	singlet state CCSD(T)	triplet state DFT	triplet state CCSD(T)
**$NOGe_2$**	N–Ge	1.8698	1.8510	1.8926	1.8651
	N–Ge′	1.8698	1.8510	1.9033	1.8704
	O–Ge	1.8346	1.8114	1.8440	1.8160
	O–Ge′	1.8346	1.8114	1.8411	1.8152
	N⋯O	2.5031	2.4815	2.5841	2.5355
	Ge⋯Ge′	2.7304	2.6932	2.6701	2.6556
	Ge–N–Ge′	93.7969	93.3571	89.4037	90.6171
	Ge–O–Ge′	96.1759	96.0418	92.8681	93.9951
	N–Ge–O	85.0136	85.3006	87.4996	87.0559
	N–Ge′–O	85.0136	85.3006	87.2628	86.9232
	∑(∡N)	360.0000	360.0000	335.2213	344.3630
	∑(∡Ge)	306.2556	309.9179	310.1428	312.8968
	∑(∡Ge′)	306.2556	309.9179	313.5106	314.9006
	N–Ge–Ge′–O	180.0000	180.0000	161.2972	167.0072
**O_2Ge_2**	O–Ge	1.8349	1.8111	1.8452	1.8178
	O⋯O′	2.5302	2.4982	2.5967	2.5433
	Ge⋯Ge′	2.6580	2.6229	2.6221	2.5980
	Ge–O–Ge′	92.8212	92.7893	90.5590	91.2182
	O–Ge–O′	87.1788	87.2107	89.4410	88.7818
	∑(∡Ge)	306.8240	310.3322	312.4796	313.6703
	O–Ge–Ge′–O′	180.0000	180.0000	180.0000	180.0000
**$F_2Ge_2^{2+}$**	F–Ge	1.9571	1.9155	1.9613	1.9155
	F⋯F′	2.3559	2.2891	2.3824	2.3120
	Ge⋯Ge′	3.1258	3.0720	3.1161	3.0546
	Ge–F–Ge′	105.9890	106.6169	105.2011	105.7552
	F–Ge–F′	74.0110	73.3831	74.7989	74.2448
	∑(∡Ge)	284.9188	285.1393	284.2650	285.5530
	F–Ge–Ge′–F′	180.0000	180.0000	180.0000	180.0000
**$Si_2Si_2^{I}$**	Si–Si″	2.2886	2.2900	2.3415	2.3478
	Si⋯Si′	3.7371	3.7169	3.5420	3.5344
	Si″⋯Si‴	2.6430	2.6762	3.0634	3.0915
	Si″–Si–Si‴	70.5370	71.5094	81.7111	82.3506
	Si–Si″–Si′	109.4630	108.4906	98.2889	97.6494
	∑(∡Si)	653.0083	653.2462	655.0513	654.9411
	∑(∡Si″)	360.0000	360.0000	338.6937	339.1389
	Si–Si″–Si‴–Si′	180.0000	180.0000	180.0000	180.0000

Table S7 continued.

	Parameter	singlet state DFT	singlet state CCSD(T)	triplet state DFT	triplet state CCSD(T)
**Si_2Si_2**	Si–Si′′	2.2956	2.2996	2.3426	2.3501
	Si···Si′	3.6612	3.6398	3.4641	3.4665
	Si′′···Si′′′	2.7703	2.8116	3.1545	3.1743
	Si′′–Si–Si′′′	74.2276	75.3699	84.6433	84.9620
	Si–Si′′–Si′	105.7724	104.6301	95.3567	95.0380
	∑(∡Si)	654.5550	654.7240	655.8646	655.7233
	∑(∡Si′′)	360.0000	360.0000	338.9509	339.9041
	Si–Si′′–Si′′′–Si′	180.0000	180.0000	180.0000	180.0000
**P_2P_2** (C_{2h})	P–P′′	2.1309	2.1322	—	—
	P···P′	2.6217	2.6431	—	—
	P′′···P′′′	3.3601	3.3465	—	—
	P′′–P–P′′′	104.0729	103.3947	—	—
	P–P′′–P′	75.9271	76.6053	—	—
	∑(∡P)	331.3756	331.8538	—	—
	P–P′′–P′′′–P′	180.0000	180.0000	—	—
**P_2P_2** (C_s)	P–P′′	—	2.1949	2.1843	2.1886
	P′–P′′	—	2.0817	2.2405	2.2461
	P···P′	—	2.6474	2.9564	2.9614
	P′′···P′′′	—	3.3543	3.0289	3.0323
	P′′–P–P′′′	—	99.6525	87.7893	87.6957
	P′′–P′–P′′′	—	107.3497	85.0540	84.9090
	P–P′′–P′	—	76.4421	83.8366	83.7811
	∑(∡P)	—	311.3710	301.6176	300.6476
	∑(∡P′)	—	350.0661	278.9898	277.9337
	P–P′′–P′′′–P′	—	175.9316	132.8675	132.4705
**N_2PAs**	N–P	1.6882	1.6840	1.7607	1.7499
	N–As	1.8762	1.8597	1.9070	1.8843
	N···N′	2.2523	2.2417	2.3636	2.3381
	P···As	2.7583	2.7407	2.6379	2.6387
	P–N–As	101.2718	101.2088	91.8900	93.0437
	N–P–N′	83.6820	83.4549	84.3193	83.8335
	N–As–N′	73.7745	74.1276	76.5914	76.6939
	∑(∡N)	359.9858	360.0000	326.5296	330.0923
	N–P–As–N′	180.0000	180.0000	136.5415	139.0610

Table S7 continued.

	Parameter	singlet state DFT	singlet state CCSD(T)	triplet state DFT	triplet state CCSD(T)
**N_2CP**	N–C	1.3538	1.3498	1.4429	1.4437
	N–P	1.7738	1.7723	1.7671	1.7564
	N···N′	2.0670	2.0628	2.1717	2.1630
	C···P	2.3161	2.3120	2.2471	2.2487
	C–N–P	94.5992	94.5816	88.2539	88.7262
	N–C–N′	99.5293	99.6601	97.6224	97.0222
	N–P–N′	71.2723	71.1767	75.8283	76.0117
	∑(∡N)	356.0615	356.6611	326.2002	327.0438
	∑(∡C)	360.0000	360.0000	336.4847	335.8586
	N–C–P–N′	180.0000	180.0000	146.4246	147.1850
**$C_2P_2^{CH2}$**	C–P	1.8230	—	1.8147	—
	C···C′	2.3939	—	2.3751	—
	P···P′	2.7500	—	2.7443	—
	P–C–P′	97.9199	—	98.2493	—
	C–P–C′	82.0801	—	81.7507	—
	∑(∡C)	360.0000	—	360.0000	—
	C–P–P′–C′	180.0000	—	180.0000	—
**$C_2P_2^{NHC1}$**	C–P	1.7994	—	1.8046	—
	C···C′	2.2845	—	2.3847	—
	P···P′	2.7808	—	2.7092	—
	P–C–P′	101.1922	—	97.2901	—
	C–P–C′	78.8078	—	82.7099	—
	∑(∡C)	360.0000	—	360.0000	—
	C–P–P′–C′	180.0000	—	180.0000	—
**$C_2P_2^{NHC2}$**	C–P	1.7963	—	1.8038	—
	C···C′	2.2909	—	2.3922	—
	P···P′	2.7675	—	2.7005	—
	P–C–P′	100.7664	—	96.9270	—
	C–P–C′	79.2336	—	83.0730	—
	∑(∡C)	360.0000	—	359.8703	—
	C–P–P′–C′	180.0000	—	180.0000	—
**O_2**	O–O′	—	—	1.2188	1.2117

Table S7 continued.

	Parameter	singlet state DFT	CCSD(T)	triplet state DFT	CCSD(T)
**C_2C_2**	C–C″	—	—	1.5077	1.5150
	C···C′	—	—	2.2016	2.2155
	C″···C‴	—	—	2.0606	2.0670
	C″–C–C‴	—	—	86.2105	86.0298
	C–C″–C′	—	—	93.7895	93.9702
	∑(∡C)	—	—	656.6709	656.2515
	∑(∡C″)	—	—	360.0000	359.6055
	C–C″–C‴–C′	—	—	180.0000	180.0000
**$C_2C_2^F$**	C–C″	1.4736	1.4819	1.5150	—
	C···C′	2.1716	2.1830	2.1569	—
	C″···C‴	1.9924	2.0048	2.1280	—
	C″–C–C‴	85.0716	85.1284	89.2263	—
	C–C″–C′	94.9284	94.8716	90.7737	—
	∑(∡C)	657.1156	656.9520	656.6078	—
	∑(∡C″)	360.0000	360.0000	350.3526	—
	C–C″–C‴–C′	180.0000	180.0000	180.0000	—
**$C_2C_2^{OH}$**	C–C″	1.4911	1.4937	1.5185	—
	C···C′	2.2165	2.2298	2.1850	—
	C″···C‴	1.9951	1.9881	2.1034	—
	C″–C–C‴	83.9811	83.4407	87.6659	—
	C–C″–C′	96.0189	96.5593	92.1813	—
	∑(∡C)	655.5481	655.5271	655.2298	—
	∑(∡C″)	360.0000	360.0000	352.0183	—
	C–C″–C‴–C′	180.0000	180.0000	180.0000	—
**Allyl$^-$**	C–C′	1.3969	1.3980	1.3970	—
	C′···C″	2.5534	2.5527	2.4659	—
	C′–C–C″	132.1083	131.8373	123.9049	—
	∑(∡C)	360.0000	360.0000	360.0000	—
	∑(∡C′)	360.0000	360.0000	360.0000	—

The structural data show that the DFT and coupled-cluster geometries are typically very similar. If we consider the coupled-cluster optimized structures as reference, the mean signed error (MSE) of the PBE functional amounts to 0.0078 Å (taking into account all individual heavy-atom interatomic distances, including transannular distances). The maximum absolute deviation is 0.0686 Å. If only bond lengths are considered (i.e.,

excluding the transannular interatomic distances), these figures drop to 0.0072 Å (MSE) and 0.0437 Å (maximum absolute deviation). The MSE of the bond angles is −0.147°, the MSE of the fold angles of the four membered ring systems amounts to −0.442°.

Even the transannular distances between the formal radical centres are well described using DFT; the MSE amounts to 0.0085 Å and is therefore only slightly larger than the error in bond lengths. There is, however, a tendency of DFT to slightly overestimate the transannular distances, especially with increasing biradical character. This may be rationalized by the missing population of the (typically bonding) LUMO of these systems, i.e., the transannular antibonding character is overestimated by DFT.

4 Vibrational frequencies

Table S8 lists the computed harmonic frequencies.

Table S8. Computed harmonic frequencies (cm^{-1}) at the PBE-D3/def2-TZVP and CCSD(T)/def2-TZVP levels of theory (allyl anion at PBE-D3/aug-cc-pVTZ and CCSD(T)/aug-cc-pVTZ). Imaginary frequencies indicated by minus sign (-).

	singlet state		triplet state			singlet state		triplet state	
	DFT	CCSD(T)	DFT	CCSD(T)		DFT	CCSD(T)	DFT	CCSD(T)
**N_2P_2**	138	209	265	263	**N_2As_2**	248	55	200	203
	368	377	456	462		273	277	308	324
	569	589	471	478		342	357	453	457
	595	621	619	651		529	577	521	562
	678	691	687	694		587	615	597	610
	750	773	732	767		606	634	611	649
	787	802	753	770		663	689	642	657
	919	948	879	905		792	832	819	841
	1089	1137	1050	1103		1037	1086	1003	1058
	1112	1165	1091	1152		1054	1110	1030	1090
	3574	3636	3463	3544		3534	3612	3427	3507
	3578	3638	3466	3547		3538	3615	3428	3509
**N_2Sb_2**	216	42	150	150	**N_2Bi_2**	166	173	119	123
	223	219	230	242		188	189	180	186
	249	251	365	351		352	317	369	377
	500	538	479	505		435	467	438	472
	530	555	501	522		480	500	468	475
	533	618	551	566		490	540	508	527
	587	657	558	600		535	559	517	536
	678	715	725	740		612	638	701	714
	960	1006	936	985		917	956	890	930
	972	1035	954	1007		920	964	902	944
	3508	3581	3425	3506		3458	3524	3380	3450
	3512	3584	3425	3508		3463	3528	3382	3450

Table S8 continued.

	singlet state		triplet state			singlet state		triplet state	
	DFT	CCSD(T)	DFT	CCSD(T)		DFT	CCSD(T)	DFT	CCSD(T)
**$P_2C_2^{Cl}$**	92	93	59	—	**P_2C_2**	223	155	164	156
	155	152	112	—		372	371	362	381
	227	210	159	—		394	410	448	454
	236	222	170	—		499	504	478	468
	257	260	242	—		554	577	620	644
	398	407	321	—		675	707	679	690
	424	417	417	—		697	720	683	738
	457	457	467	—		754	772	729	751
	470	468	641	—		757	782	752	753
	652	662	688	—		810	831	763	801
	693	730	722	—		899	914	797	821
	774	805	728	—		942	950	839	848
	813	836	801	—		969	1010	1019	1071
	815	837	884	—		1012	1056	1065	1117
	937	964	961	—		2264	2389	2120	2263
	1014	1038	1053	—		2273	2394	2138	2275
	2288	2407	2128	—		3197	3252	3163	3214
	2294	2410	2148	—		3200	3254	3168	3217
**P_2B_2**	-193	-178	98	86	**P_2Al_2**	-148	-155	50	45
	355	373	155	156		163	171	186	191
	436	446	344	348		200	172	286	310
	563	586	390	404		216	211	306	312
	618	645	440	481		403	412	319	316
	631	654	497	536		419	430	323	333
	699	723	591	621		434	445	335	338
	700	724	604	633		437	463	353	358
	709	726	617	634		454	464	377	370
	723	745	625	644		503	533	417	458
	753	764	627	665		535	570	439	463
	757	767	664	712		550	575	466	500
	760	795	737	752		557	594	530	557
	805	849	798	841		578	600	592	610
	882	922	859	906		628	654	603	645
	944	984	871	907		654	674	614	653
	1176	1226	1050	1114		1138	1183	1041	1088
	1209	1258	1092	1152		1166	1210	1067	1109
	2193	2343	2275	2414		1895	1964	1823	1899
	2202	2350	2290	2423		1898	1967	1825	1901
	2247	2385	2296	2427		2218	2361	2344	2466
	2257	2391	2306	2433		2228	2367	2348	2466
	2606	2667	2652	2711		2277	2404	2348	2472
	2608	2667	2653	2712		2281	2406	2349	2474

Table S8 continued.

	singlet state		triplet state			singlet state		triplet state	
	DFT	CCSD(T)	DFT	CCSD(T)		DFT	CCSD(T)	DFT	CCSD(T)
**P_2Ga_2**	-213	-177	42	44	**S_2N_2**	465	475	331	275
	-60	-56	139	146		613	643	481	507
	153	149	240	250		653	651	567	578
	248	217	242	265		765	796	583	612
	296	316	263	280		896	917	647	648
	344	356	266	291		898	922	736	752
	350	370	283	310					
	400	411	346	360					
	413	450	348	361					
	475	511	420	451					
	511	552	428	456					
	515	557	454	498					
	536	590	537	571					
	610	644	605	632	**O_3**	714	717	203	568
	625	659	606	643		1066	1061	569	593
	656	674	608	644		1188	1153	1054	1064
	1138	1181	1039	1090					
	1165	1207	1065	1112					
	1918	2003	1823	1920					
	1931	2012	1826	1923					
	2230	2371	2344	2461					
	2239	2376	2344	2462					
	2281	2408	2348	2469					
	2284	2408	2352	2471					
**N_2C_2**	399	381	150	112	**N_2Si_2**	54	152	134	148
	487	509	537	643		281	284	331	279
	536	539	663	711		333	472	401	420
	678	679	778	816		506	525	507	529
	788	839	816	842		510	530	563	600
	957	979	947	983		579	623	602	637
	1017	1042	990	1009		661	678	668	705
	1029	1055	1000	1019		678	741	687	741
	1083	1107	1016	1042		739	762	702	750
	1211	1247	1127	1148		751	789	769	802
	1264	1279	1169	1214		819	852	824	854
	1300	1332	1237	1283		875	906	855	890
	1313	1354	1259	1305		942	993	967	1006
	1351	1393	1311	1358		993	1042	999	1048
	3088	3164	3053	3146		1997	2125	1971	2100
	3088	3166	3104	3174		1998	2128	1979	2108
	3550	3634	3232	3401		3604	3665	3571	3653
	3553	3636	3322	3439		3608	3667	3577	3657

Table S8 continued.

	singlet state		triplet state			singlet state		triplet state	
	DFT	CCSD(T)	DFT	CCSD(T)		DFT	CCSD(T)	DFT	CCSD(T)
**N_2Si_2**	-1197	-1334	—	—	**N_2Ge_2**	215	231	105	126
(D_{2h})	-1114	-1020	—	—		251	241	256	268
	53	128	—	—		277	321	358	341
	359	363	—	—		336	331	445	446
	506	515	—	—		453	467	474	504
	539	565	—	—		510	555	524	571
	578	621	—	—		543	569	532	601
	593	629	—	—		589	635	565	621
	682	711	—	—		623	666	593	638
	777	790	—	—		648	688	628	675
	890	919	—	—		658	688	648	690
	907	930	—	—		753	795	753	791
	964	1006	—	—		952	995	967	1020
	1007	1048	—	—		1006	1045	978	1034
	2071	2379	—	—		1854	1985	1799	1961
	2299	2381	—	—		1857	1986	1806	1968
	3613	3672	—	—		3523	3610	3491	3584
	3615	3673	—	—		3528	3613	3497	3588
**N_2Sn_2**	165	186	93	110	**$NOGe_2$**	211	243	133	137
	200	210	178	200		244	331	278	281
	231	249	323	329		307	347	415	382
	314	274	346	373		390	423	462	495
	383	422	407	413		500	554	505	560
	430	457	422	444		525	556	535	600
	450	472	434	505		585	637	566	629
	474	512	448	505		612	658	595	646
	515	555	484	534		644	678	625	669
	547	585	537	579		655	701	633	690
	550	596	546	605		726	772	706	753
	631	674	668	699		958	1007	943	996
	888	926	891	942		1863	1985	1788	1954
	949	985	905	958		1864	1988	1813	1977
	1629	1756	1565	1728		3502	3585	3470	3582
	1630	1760	1569	1733					
	3477	3558	3443	3542					
	3481	3562	3448	3546					

Table S8 continued.

	singlet state		triplet state			singlet state		triplet state	
	DFT	CCSD(T)	DFT	CCSD(T)		DFT	CCSD(T)	DFT	CCSD(T)
**O_2Ge_2**	213	268	118	156	**$F_2Ge_2^{2+}$**	155	-311	124	139
	264	344	303	334		235	126	229	254
	313	379	434	492		249	192	231	266
	495	542	503	557		326	249	356	419
	503	554	518	605		347	319	366	444
	582	656	590	664		406	410	412	459
	612	675	592	675		406	445	413	461
	634	676	620	698		493	453	489	552
	645	694	639	701		496	462	496	570
	692	748	659	725		502	555	502	586
	1867	2009	1795	1958		1571	1731	1502	1806
	1867	2013	1800	1993		1576	1780	1516	1810
**$Si_2Si_2^{I}$**	-108	—	6	—	**Si_2Si_2**	-274	-246	48	47
	-20	—	56	—		42	50	221	246
	65	—	66	—		247	252	231	262
	100	—	102	—		383	399	372	384
	104	—	108	—		398	422	376	395
	158	—	180	—		451	464	379	404
	217	—	237	—		455	465	414	425
	323	—	296	—		459	495	420	438
	369	—	399	—		503	514	422	440
	436	—	408	—		535	564	427	455
	442	—	423	—		550	583	492	527
	447	—	433	—		576	597	525	570
	498	—	444	—		591	609	537	574
	511	—	468	—		598	639	616	657
	540	—	500	—		613	676	675	707
	559	—	504	—		741	770	675	728
	596	—	542	—		943	984	882	933
	608	—	653	—		971	1009	904	944
	931	—	881	—		2064	2196	2091	2201
	965	—	905	—		2070	2200	2093	2201
	2128	—	2129	—		2115	2216	2118	2219
	2130	—	2132	—		2116	2216	2122	2220
	2132	—	2141	—		2117	2218	2127	2227
	2133	—	2142	—		2121	2221	2127	2227

Table S8 continued.

	singlet state		triplet state			singlet state		triplet state	
	DFT	CCSD(T)	DFT	CCSD(T)		DFT	CCSD(T)	DFT	CCSD(T)
**P_2P_2** (C_{2h})	52	-87	—	—	**P_2P_2** (C_s)	—	108	151	156
	220	226	—	—		—	208	232	248
	312	329	—	—		—	328	403	408
	494	508	—	—		—	471	405	418
	504	509	—	—		—	479	440	445
	521	525	—	—		—	535	454	454
	525	532	—	—		—	571	612	636
	640	662	—	—		—	677	617	662
	723	756	—	—		—	734	643	688
	754	791	—	—		—	822	703	745
	2287	2404	—	—		—	2399	2232	2345
	2294	2409	—	—		—	2410	2242	2358
**N_2PAs**	35	164	237	238	**N_2CP**	343	347	348	351
	316	329	379	399		425	437	516	524
	432	450	463	454		470	538	547	572
	573	606	549	583		614	623	673	704
	598	625	609	626		624	636	697	721
	640	659	708	736		653	662	840	900
	816	833	718	736		859	879	900	931
	881	908	848	871		1063	1094	1019	1037
	1059	1105	1028	1080		1133	1164	1043	1066
	1083	1135	1064	1122		1217	1262	1109	1158
	3564	3623	3443	3523		1342	1371	1235	1287
	3568	3626	3444	3526		1443	1486	1251	1300
						3168	3239	2935	3035
						3577	3649	3466	3543
						3579	3650	3469	3545

Table S8 continued.

	singlet state		triplet state	
	DFT	CCSD(T)	DFT	CCSD(T)
**$C_2P_2^{CH2}$**	45	—	73	—
	223	—	230	—
	287	—	288	—
	336	—	352	—
	371	—	380	—
	398	—	434	—
	528	—	520	—
	551	—	535	—
	565	—	551	—
	590	—	598	—
	654	—	707	—
	677	—	726	—
	878	—	862	—
	879	—	863	—
	1066	—	1072	—
	1079	—	1093	—
	1363	—	1317	—
	1386	—	1383	—
	1529	—	1477	—
	1572	—	1545	—
	3051	—	3050	—
	3052	—	3051	—
	3134	—	3134	—
	3134	—	3135	—
**$C_2P_2^{NHC1}$**	34	—	31	—
	49	—	56	—
	59	—	78	—
	64	—	104	—
	110	—	106	—
	134	—	127	—
	169	—	130	—
	222	—	227	—
	227	—	240	—
	249	—	253	—
	286	—	259	—
	337	—	265	—
	371	—	363	—
	377	—	364	—
	408	—	377	—
	503	—	477	—
	505	—	501	—
	507	—	502	—
**$C_2P_2^{NHC1}$**	528	—	565	—
cont'd.	554	—	587	—
	667	—	609	—
	667	—	614	—
	691	—	702	—
	693	—	703	—
	817	—	808	—
	854	—	822	—
	860	—	847	—
	867	—	862	—
	899	—	898	—
	903	—	901	—
	930	—	930	—
	934	—	931	—
	994	—	967	—
	1014	—	993	—
	1026	—	1030	—
	1028	—	1032	—
	1083	—	1076	—
	1085	—	1079	—
	1087	—	1089	—
	1103	—	1099	—
	1170	—	1166	—
	1170	—	1166	—
	1186	—	1185	—
	1193	—	1189	—
	1259	—	1242	—
	1259	—	1243	—
	1286	—	1285	—
	1295	—	1292	—
	1334	—	1331	—
	1335	—	1332	—
	1356	—	1378	—
	1394	—	1383	—
	1419	—	1384	—
	1420	—	1395	—
	1468	—	1463	—
	1471	—	1463	—
	1471	—	1467	—
	1471	—	1468	—
	1556	—	1583	—
	1628	—	1641	—

Table S8 continued.

	singlet state		triplet state			singlet state		triplet state	
	DFT	CCSD(T)	DFT	CCSD(T)		DFT	CCSD(T)	DFT	CCSD(T)
**$C_2P_2^{NHC1}$**	2919	—	2909	—	**$C_2P_2^{NHC2}$**	752	—	740	—
cont'd.	2920	—	2910	—	*cont'd.*	752	—	745	—
	2931	—	2922	—		838	—	755	—
	2931	—	2922	—		872	—	835	—
	3027	—	3021	—		872	—	856	—
	3027	—	3022	—		875	—	864	—
	3031	—	3024	—		903	—	891	—
	3031	—	3025	—		904	—	892	—
	3528	—	3495	—		994	—	968	—
	3529	—	3496	—		1029	—	1007	—
	3529	—	3496	—		1049	—	1040	—
	3530	—	3496	—		1051	—	1042	—
**$C_2P_2^{NHC2}$**	35	—	28	—		1113	—	1109	—
	42	—	53	—		1114	—	1110	—
	51	—	73	—		1118	—	1115	—
	55	—	94	—		1123	—	1115	—
	117	—	103	—		1243	—	1226	—
	122	—	118	—		1243	—	1226	—
	199	—	124	—		1328	—	1341	—
	245	—	222	—		1356	—	1342	—
	294	—	237	—		1357	—	1344	—
	340	—	267	—		1371	—	1369	—
	376	—	331	—		1392	—	1370	—
	379	—	332	—		1393	—	1371	—
	396	—	342	—		1549	—	1579	—
	414	—	356	—		1558	—	1580	—
	420	—	366	—		1584	—	1613	—
	464	—	373	—		1633	—	1649	—
	487	—	392	—		3214	—	3208	—
	504	—	477	—		3214	—	3208	—
	592	—	574	—		3233	—	3228	—
	594	—	575	—		3233	—	3228	—
	626	—	603	—		3576	—	3565	—
	632	—	611	—		3578	—	3565	—
	672	—	639	—		3578	—	3569	—
	682	—	640	—		3579	—	3569	—
**O_2**	—	—	1556	1585					

Table S8 continued.

	singlet state		triplet state			singlet state		triplet state	
	DFT	CCSD(T)	DFT	CCSD(T)		DFT	CCSD(T)	DFT	CCSD(T)
**C_2C_2**	—	—	168	105	**$C_2C_2^F$**	-318	-290	78	—
	—	—	196	139		150	163	198	—
	—	—	212	203		201	217	289	—
	—	—	725	756		354	378	290	—
	—	—	828	849		361	381	344	—
	—	—	890	902		380	400	360	—
	—	—	903	918		394	415	383	—
	—	—	909	948		503	536	485	—
	—	—	936	957		548	578	557	—
	—	—	956	1009		633	672	558	—
	—	—	994	1049		709	713	639	—
	—	—	1068	1067		788	820	657	—
	—	—	1152	1196		891	914	762	—
	—	—	1183	1235		938	972	937	—
	—	—	1234	1284		945	1044	947	—
	—	—	1282	1325		975	1047	987	—
	—	—	1371	1445		986	1102	1005	—
	—	—	1392	1465		1099	1131	1035	—
	—	—	2833	2947		1226	1263	1120	—
	—	—	2840	2955		1228	1265	1188	—
	—	—	2842	2964		1280	1333	1238	—
	—	—	2851	2968		1358	1426	1260	—
	—	—	3137	3211		3105	3188	3139	—
	—	—	3137	3212		3110	3194	3141	—
**$C_2C_2^{OH}$**	-328	—	67	—	**$C_2C_2^{OH}$**	897	—	931	—
	99	—	199	—	*cont'd.*	937	—	941	—
	161	—	229	—		937	—	956	—
	201	—	266	—		1021	—	984	—
	265	—	276	—		1043	—	1028	—
	329	—	287	—		1074	—	1098	—
	334	—	325	—		1173	—	1133	—
	381	—	336	—		1204	—	1174	—
	385	—	340	—		1277	—	1293	—
	437	—	390	—		1312	—	1301	—
	449	—	415	—		1325	—	1324	—
	505	—	512	—		1375	—	1364	—
	523	—	528	—		3082	—	3114	—
	614	—	560	—		3086	—	3115	—
	673	—	640	—		3642	—	3639	—
	774	—	643	—		3642	—	3639	—
	816	—	765	—		3645	—	3651	—
	852	—	914	—		3646	—	3652	—

Table S8 continued.

	singlet state		triplet state			singlet state		triplet state	
	DFT	CCSD(T)	DFT	CCSD(T)		DFT	CCSD(T)	DFT	CCSD(T)
**$C_3C_2^F$**	35	-110	90	—	**Allyl⁻**	339	313	266	—
	146	45	123	—		413	391	356	—
	297	311	245	—		414	416	397	—
	377	369	356	—		474	471	421	—
	403	427	398	—		627	639	685	—
	415	430	428	—		886	907	885	—
	572	583	442	—		930	958	939	—
	602	646	515	—		985	1004	964	—
	708	724	624	—		1184	1218	1177	—
	736	747	643	—		1231	1251	1202	—
	753	815	783	—		1361	1406	1366	—
	824	852	847	—		1425	1474	1448	—
	831	937	887	—		1520	1548	1471	—
	907	961	895	—		2945	3025	3044	—
	921	961	924	—		3027	3094	3068	—
	966	972	951	—		3035	3100	3076	—
	988	1071	1024	—		3108	3176	3152	—
	1056	1092	1048	—		3113	3180	3157	—
	1078	1134	1073	—					
	1129	1202	1181	—					
	1219	1252	1189	—					
	1232	1293	1231	—					
	1292	1296	1271	—					
	1328	1374	1306	—					
	1384	1439	1344	—					
	1393	1472	1399	—					
	1402	1491	1413	—					
	2923	2997	2879	—					
	2923	3000	2886	—					
	2930	3026	2983	—					
	2939	3039	2986	—					
	3133	3185	3161	—					
	3135	3189	3163	—					

5 Active space orbitals

Figures S1-S16 show the orbitals in the active space for selected examples of the biradicaloids, both in the delocalized and localized pictures. The orbitals were rendered from the state-specific calculations; the state-averaged orbitals are qualitatively very similar. All orbitals are plotted at iso = 0.04 a.u.

Figure S1. Active space orbitals of $\mathbf{N_2P_2}$ (analogous: $\mathbf{N_2As_2}$, $\mathbf{N_2Sb_2}$, $\mathbf{N_2Bi_2}$, $\mathbf{N_2PAs}$).

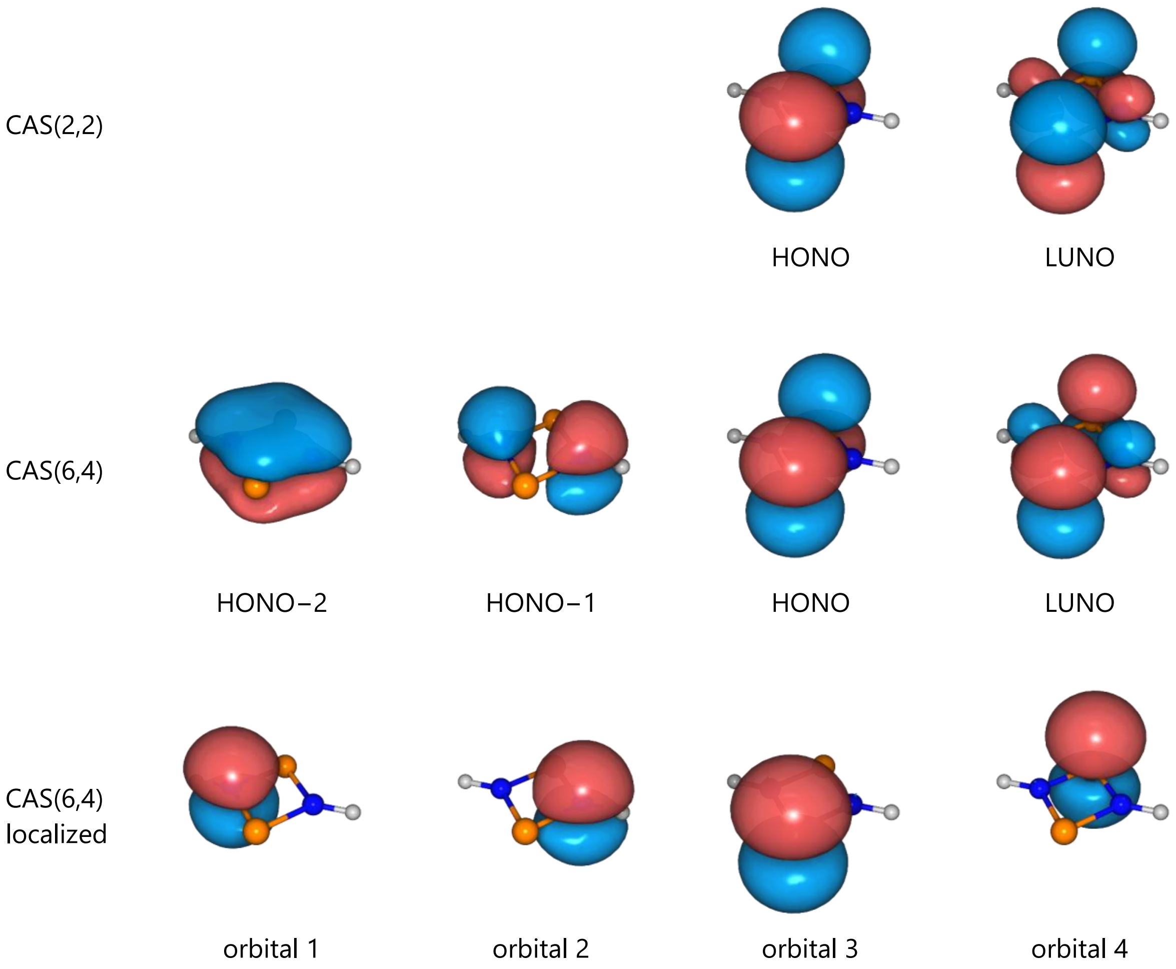

Figure S2. Active space orbitals of $\mathbf{N_2Si_2}$ (D_{2h}; see Figure S9 for C_{2h}).

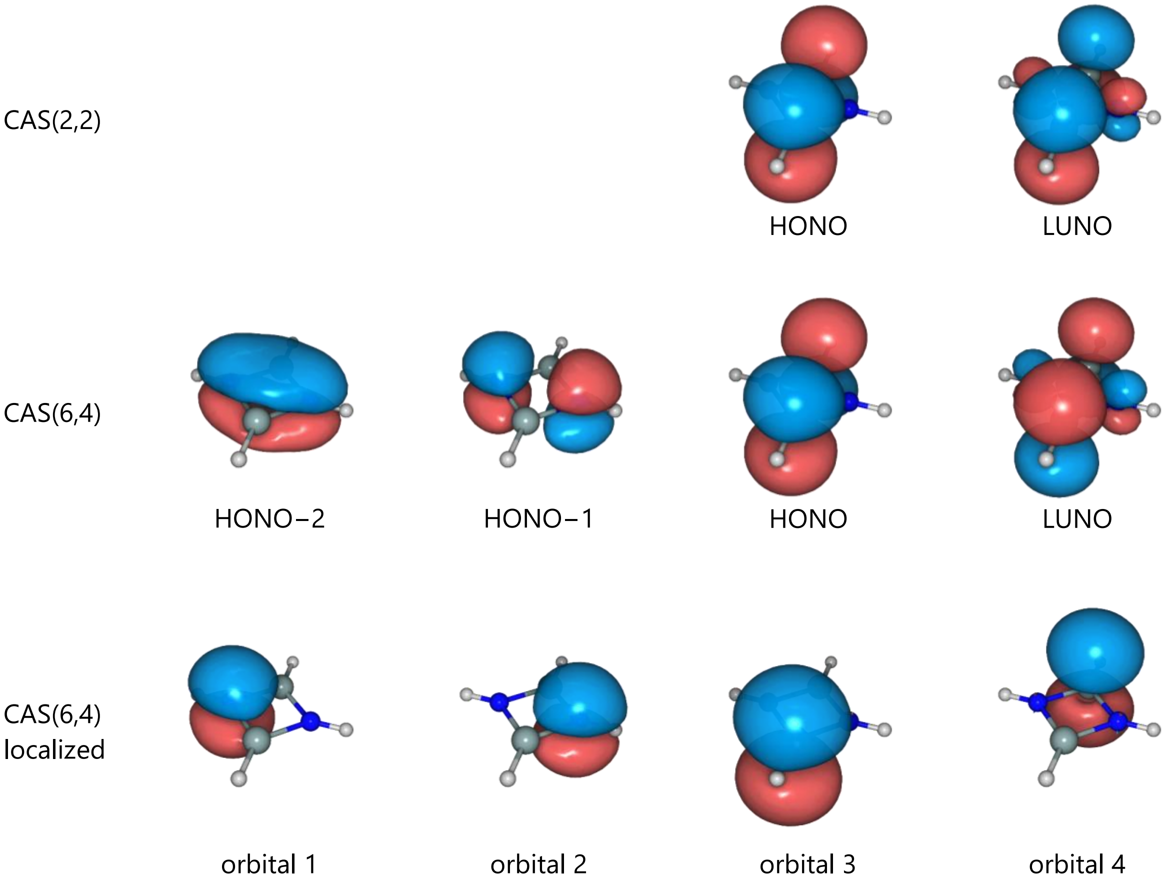

Figure S3. Active space orbitals of $\mathbf{S_2N_2}$.

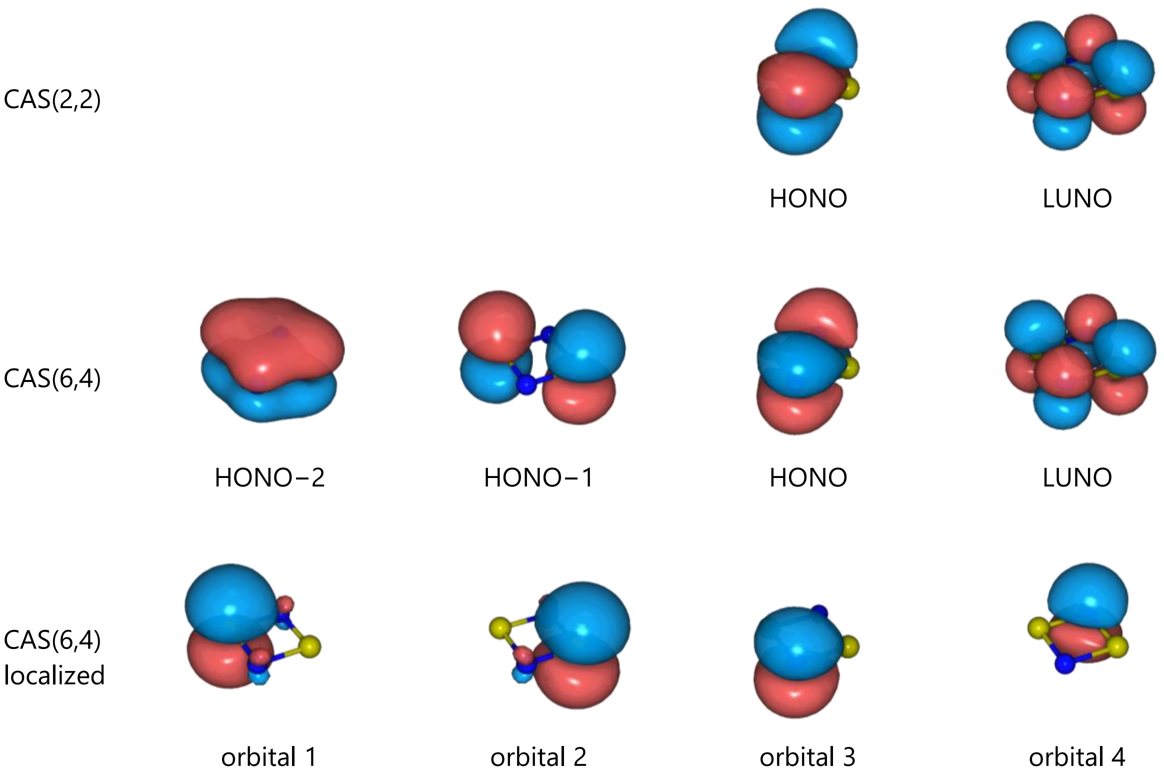

Figure S4. Active space orbitals of **N_2CP**.

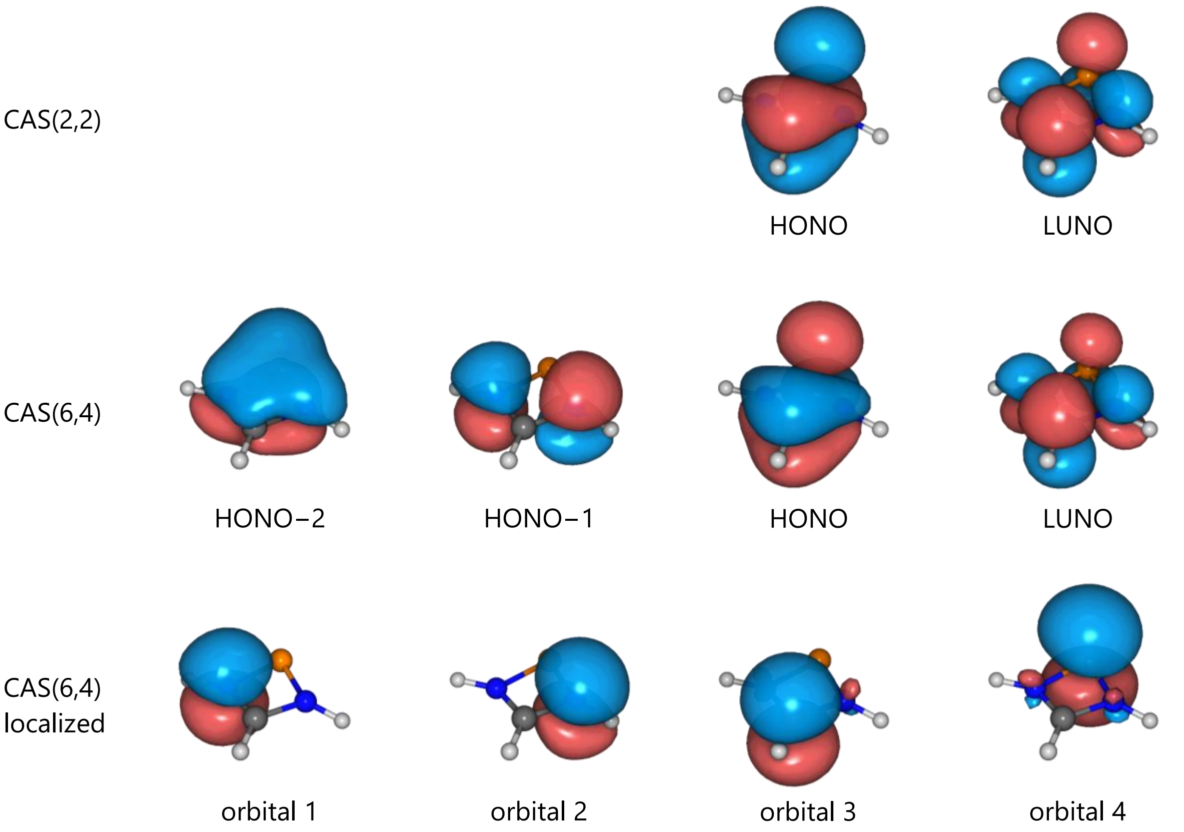

Figure S5. Active space orbitals of **P_2C_2** (analogous: **$P_2C_2^{Cl}$**, **P_2P_2** C_{2h}).

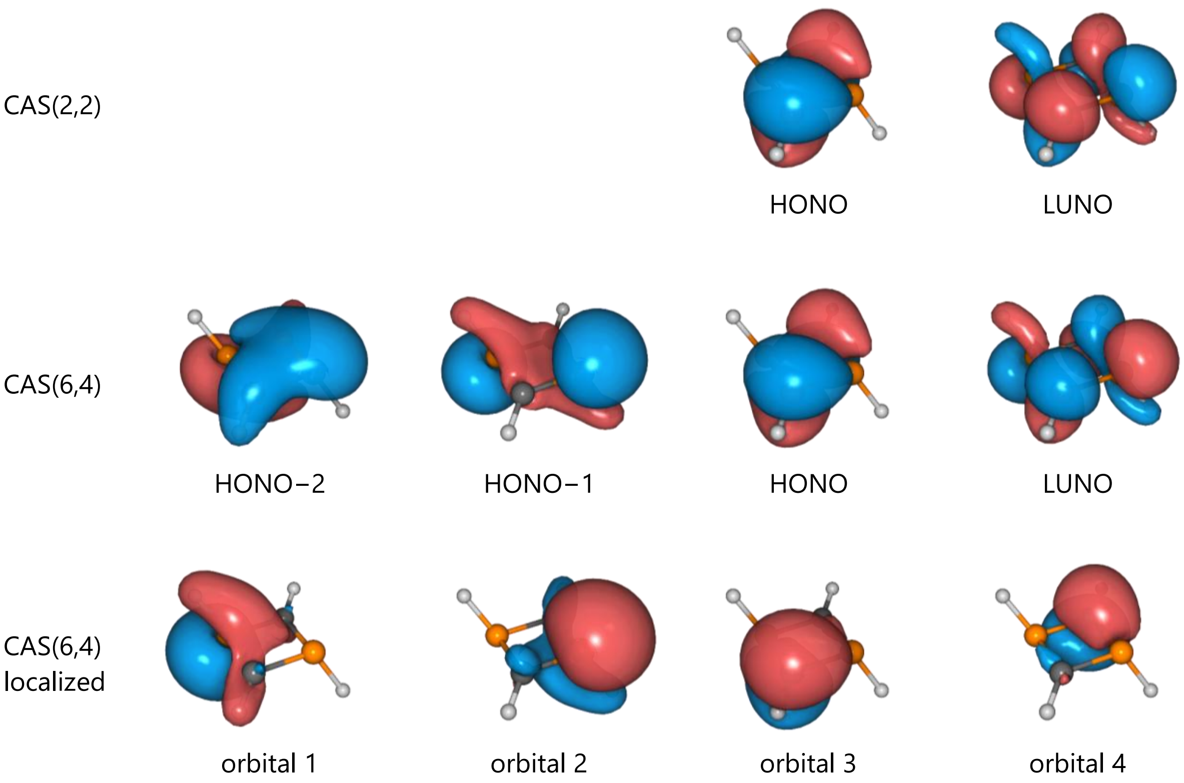

Figure S6. Active space orbitals of **P_2P_2** (C_S).

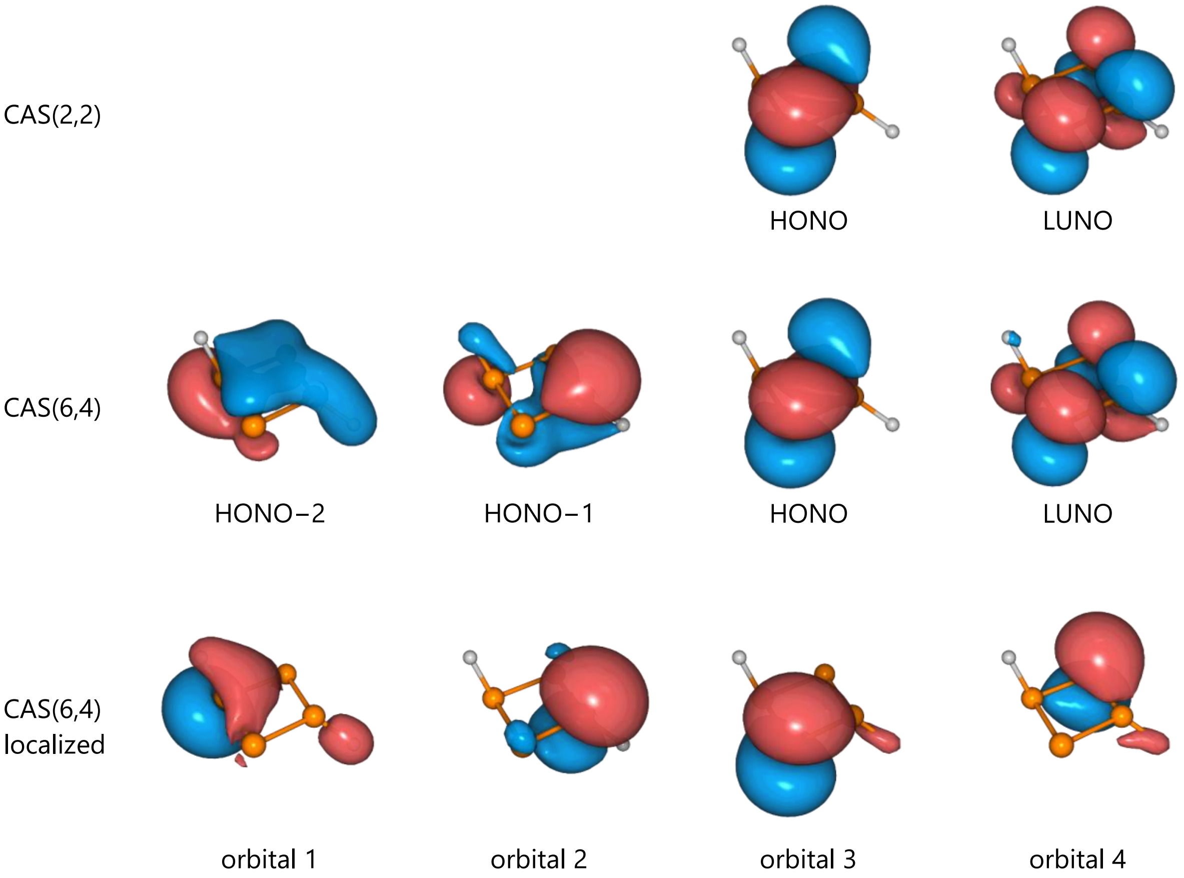

Figure S7. Active space orbitals of **O_3** (analogous: **allyl**$^-$).

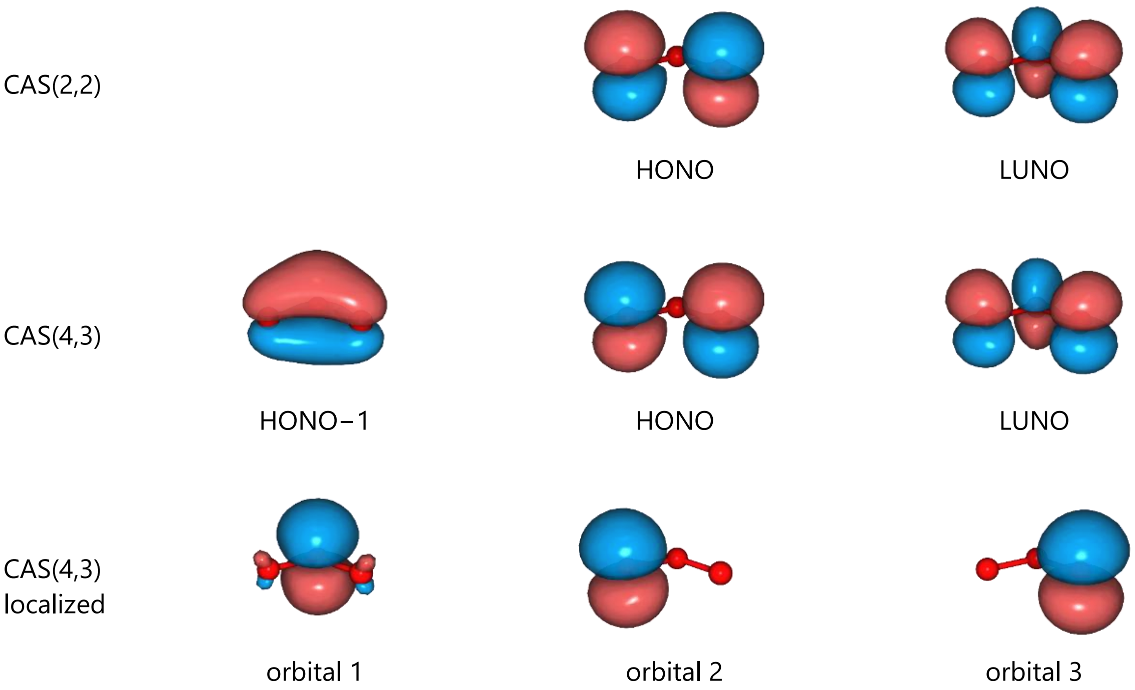

Figure S8. Active space orbitals of **N_2C_2**.

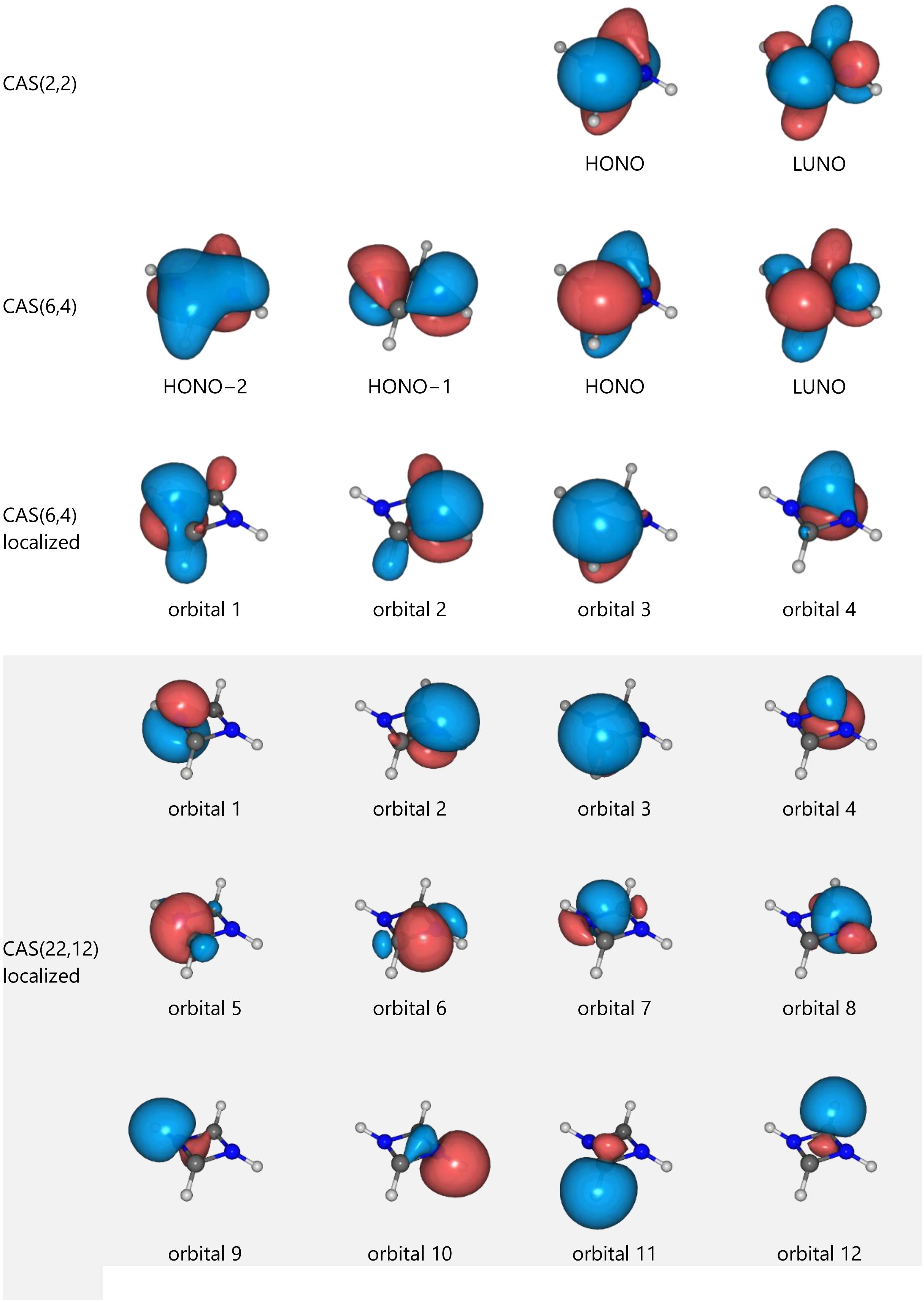

Figure S9. Active space orbitals of $\mathbf{N_2Si_2}$ (analogous: $\mathbf{N_2Ge_2}$, $\mathbf{N_2Sn_2}$, $\mathbf{NOGe_2}$, $\mathbf{O_2Ge_2}$, $\mathbf{F_2Ge_2^{2+}}$).

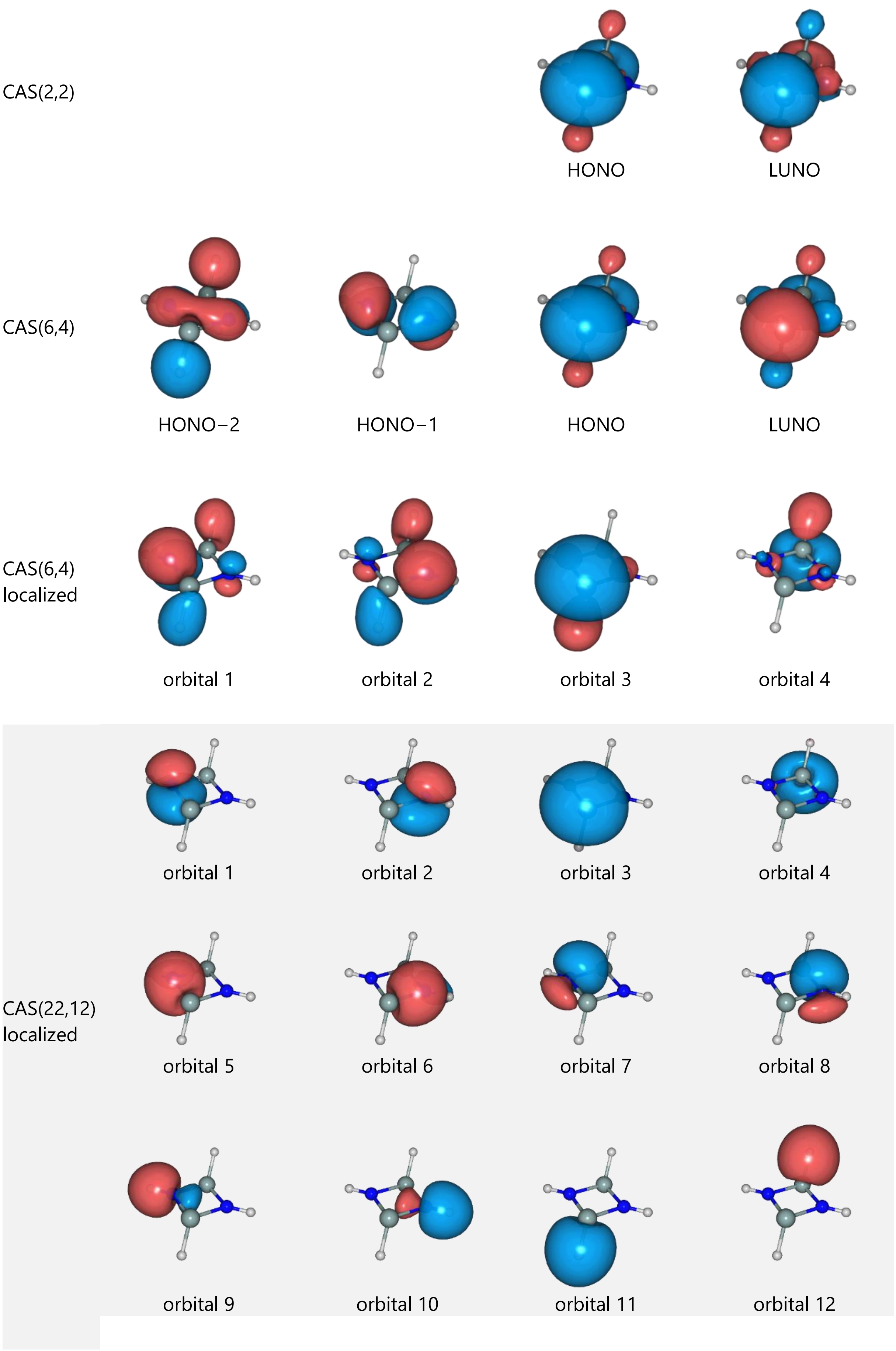

Figure S10. Active space orbitals of $\mathbf{P_2B_2}$ (analogous: $\mathbf{P_2Al_2}$, $\mathbf{P_2Ga_2}$, $\mathbf{Si_2Si_2}$, $\mathbf{Si_2Si_2^{I}}$).

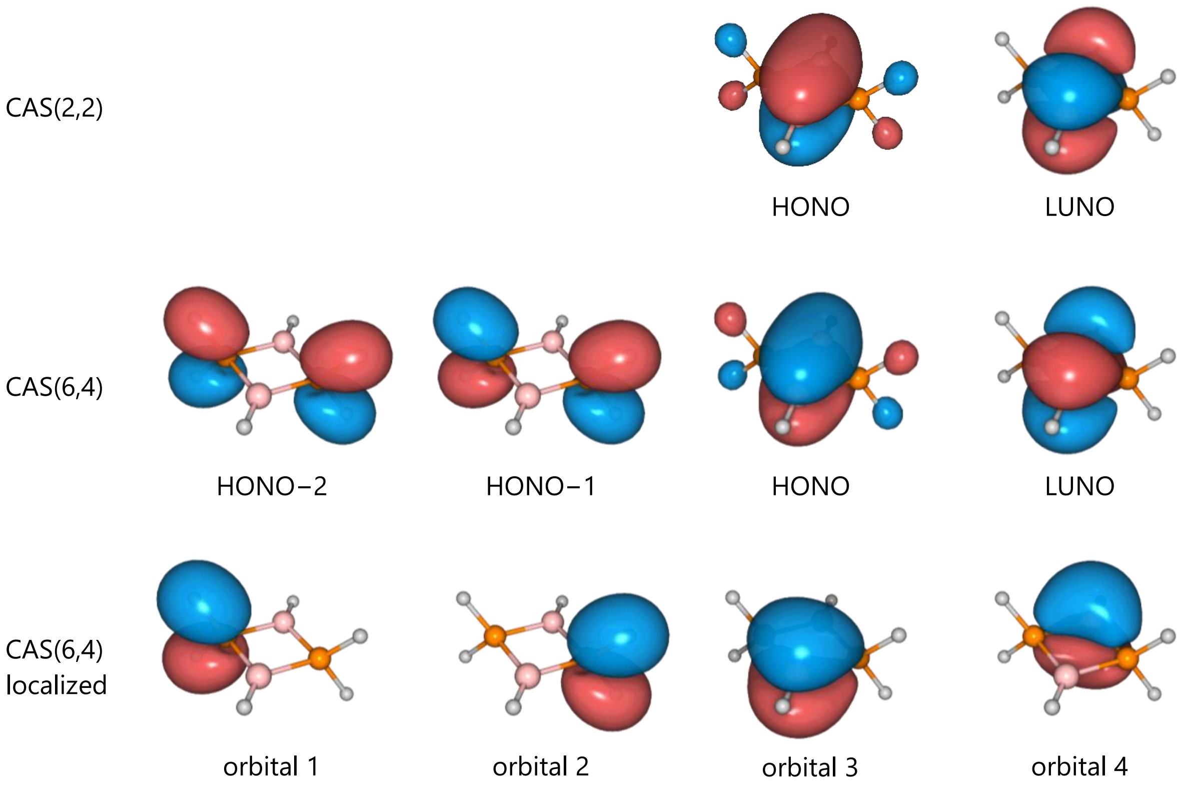

Figure S11. Active space orbitals of $\mathbf{C_2P_2^{CH_2}}$.

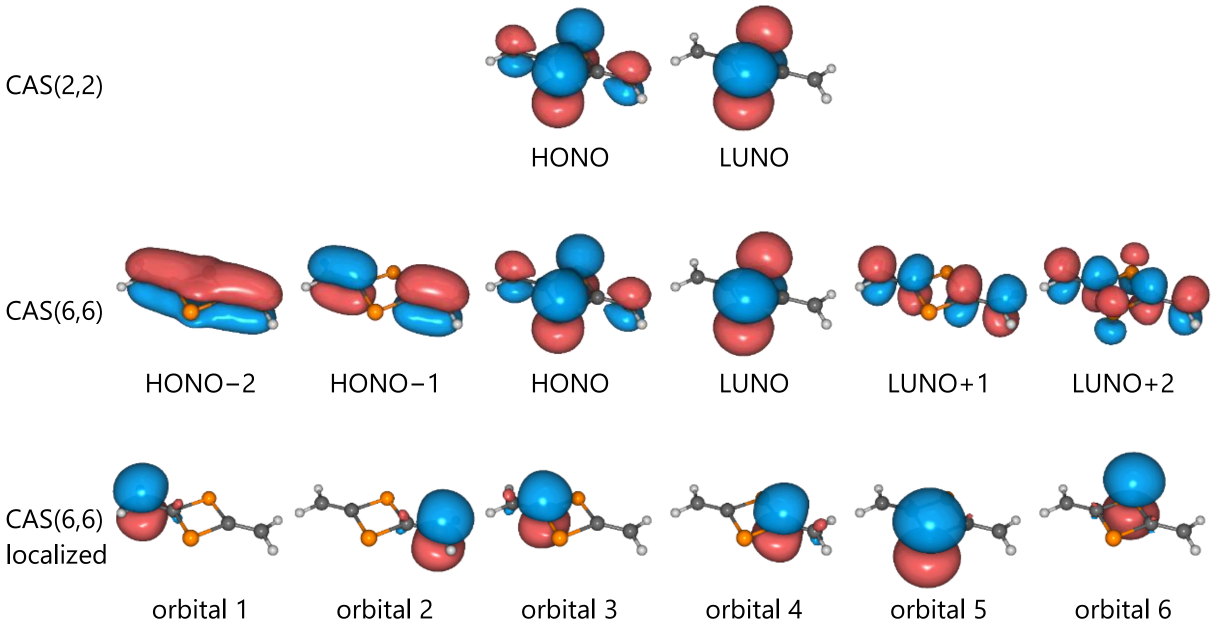

Figure S12. Active space orbitals of $\mathbf{C_2P_2}^{\mathbf{NHC1}}$ (analogous: $\mathbf{C_2P_2}^{\mathbf{NHC2}}$).

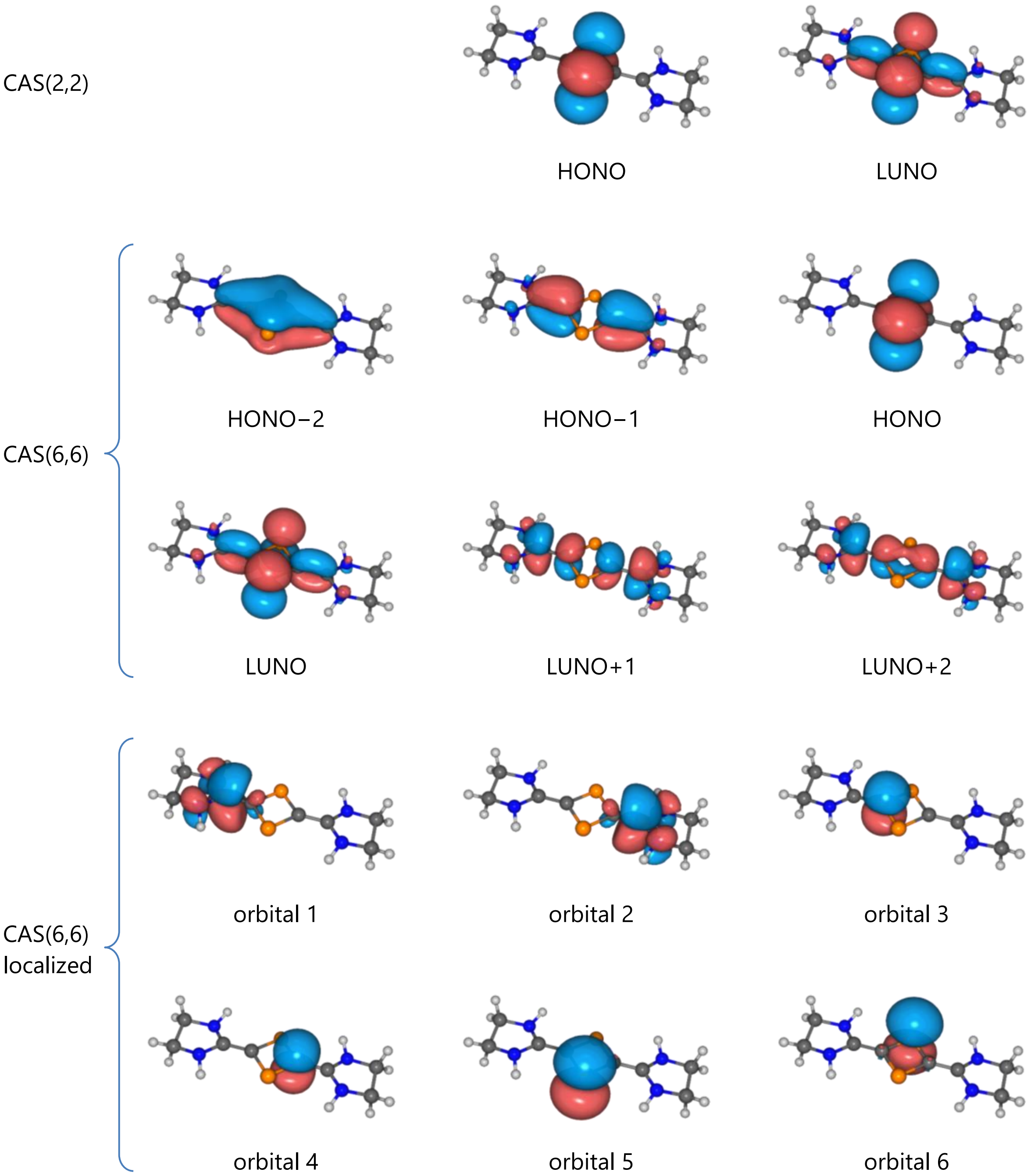

Figure S13. Active space orbitals of $\mathbf{O_2}$.

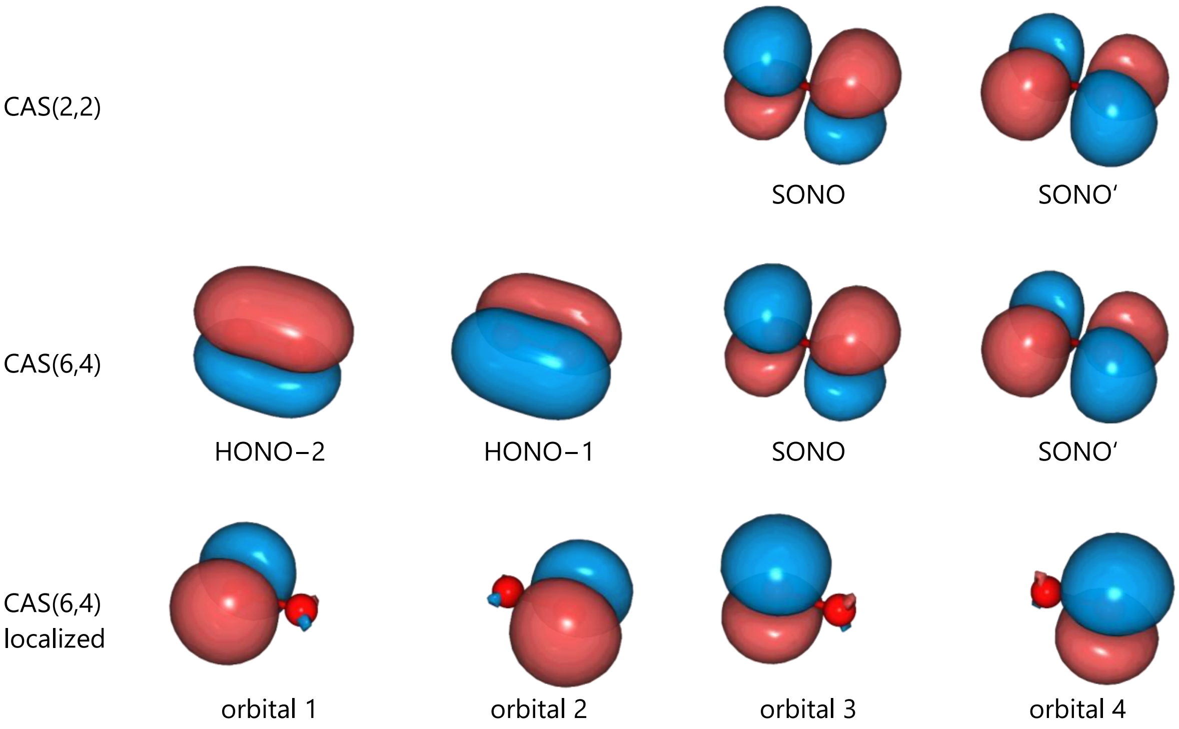

Figure S14. Active space orbitals of $\mathbf{C_2C_2}$ (analogous: $\mathbf{C_2C_2^F}$, $\mathbf{C_2C_2^{OH}}$).

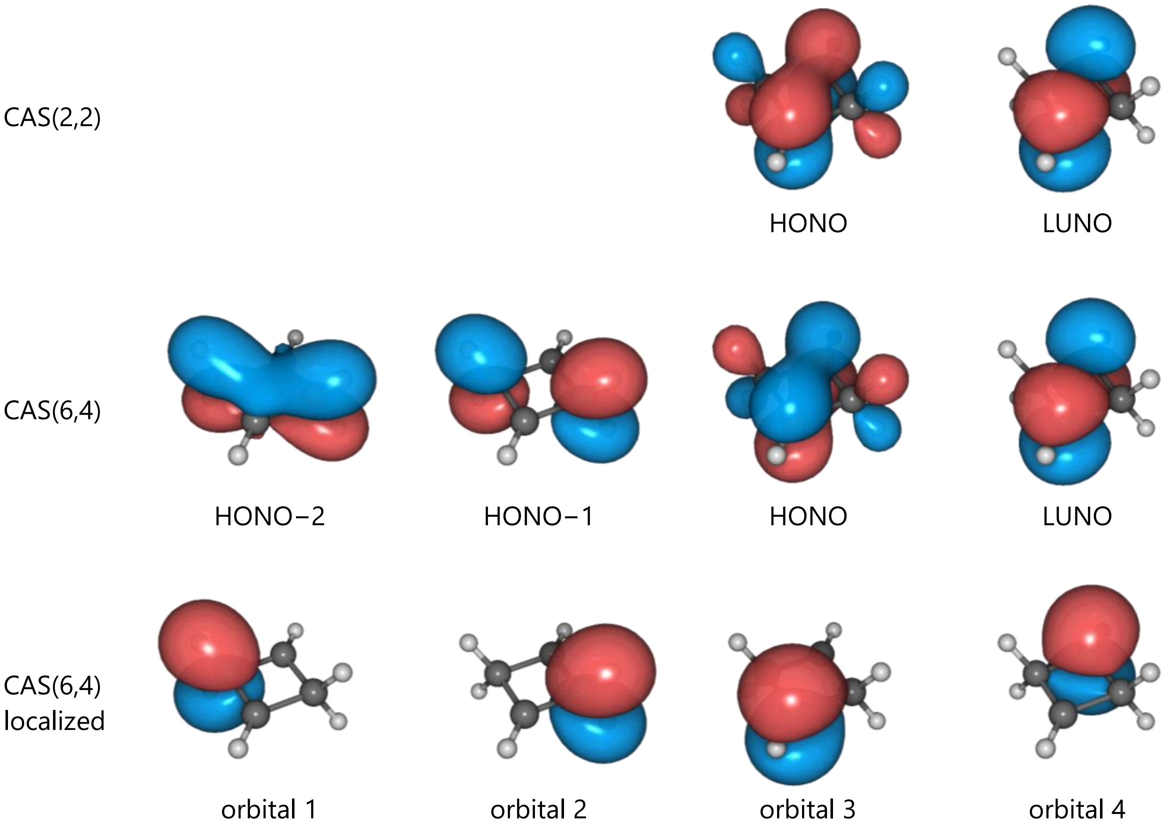

Figure S15. Active space orbitals of $\mathbf{C_3C_2^F}$.

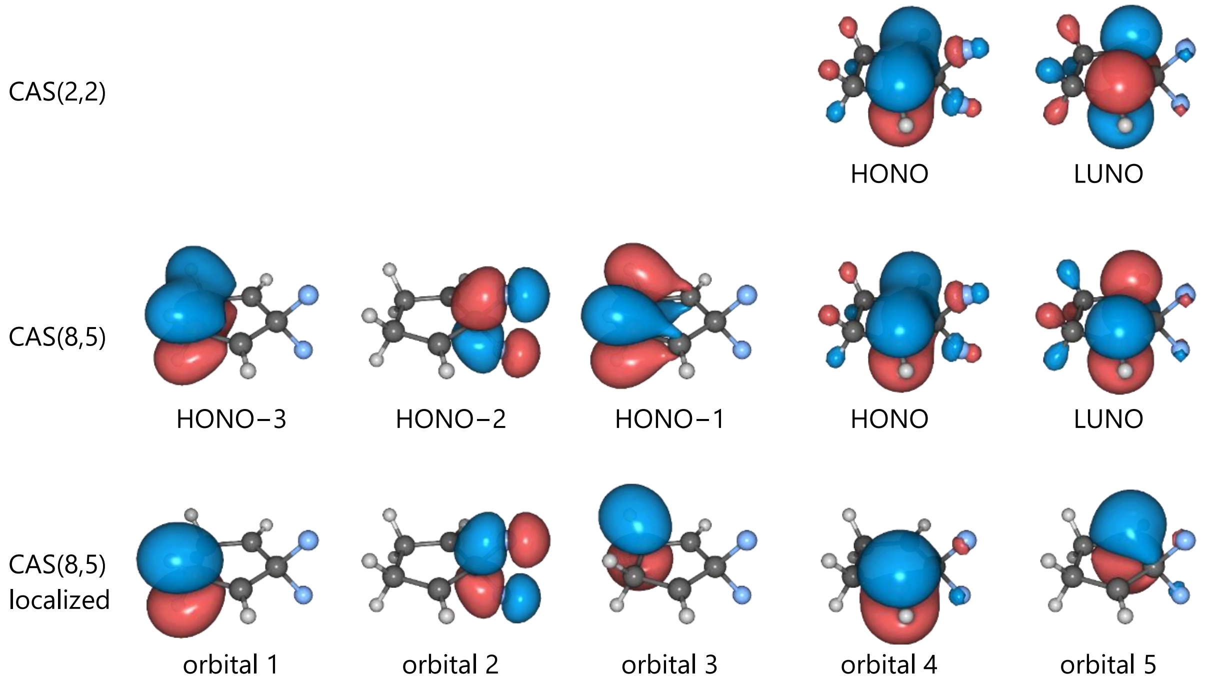

Figure S16. Active space orbitals of $\mathbf{N_2PAs}$ (arsinidene character; analogous: $\mathbf{N_2CP}$).

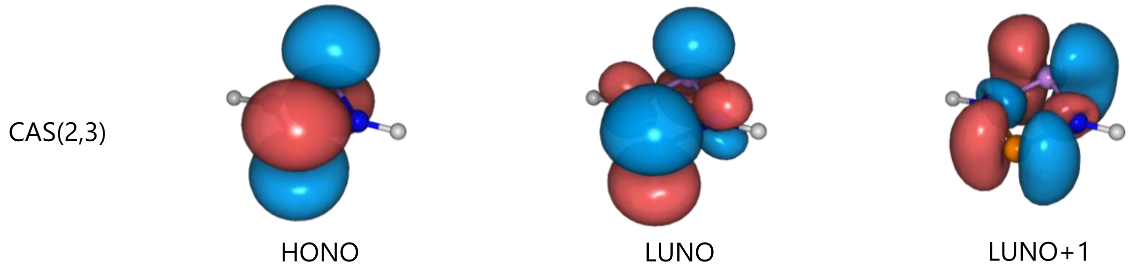

6 Lewis resonance schemes

The formal π-bonding electrons were localized using Foster-Boys localization[71,72] of the active space (including all said π-type orbitals, i.e. employing a CASSCF(6,4) wavefunction for the majority of the structures; see also section 2). In case of planar or nearly planar molecules, this procedure typically resulted in a set of orthogonal orbitals that are nicely localized on single atoms, with negligible "orthogonalization tails" at the other atoms. In those instances where the substituents are significantly bent out of the plane of the four-membered ring, typically a mixing of the π and σ bonding systems is observed, resulting in less well localized orbitals (at least when restricting the active space to six electrons in four orbitals). Much larger active spaces are then needed to obtain orbitals that are exclusively localized at single atoms, which severely hampers the evaluation of the wavefunction in terms of a Lewis resonance scheme. The effect of different active spaces was exemplarily investigated for **N_2C_2** and **N_2Si_2** indicating only a moderate effect on the relative weights of different Lewis structures as well as the biradical descriptors listed in Table S4.

In any case, electron localization is always somewhat arbitrary and a variety of other methods to generate a valence-bond (VB) type description of the wavefunction exist, so for reasons of simplicity and consistency, we opted to use a minimal active space (i.e. (6,4) in most cases). We do want to stress that the reported weights should be regarded as a guideline, rather than as an exact figure.

Schemes S1 and S2 show the important resonance structures that describe the electronic structure of the formal π-bonding system. Resonance structures with weights lower than 1% are omitted. For symmetry equivalent resonance structures, the sum of weights is reported. Single π* bonds (π-antibonds) are indicated by "•⋯•", while single π-bonds are shown as "•—•".

Scheme S1. Lewis-type resonance structures and weights according to the localization of the CASSCF(6,4)/def2-TZVP wavefunction (unless noted otherwise). For reasons of clarity, the H atoms of E-H bonds are not printed (i.e., only the bonding electrons are shown). •····• = single π-antibond, •—• = single π-bond (threshold: $|BO/w_i| > 0.2$). Sums of weights are reported for symmetry equivalent structures.

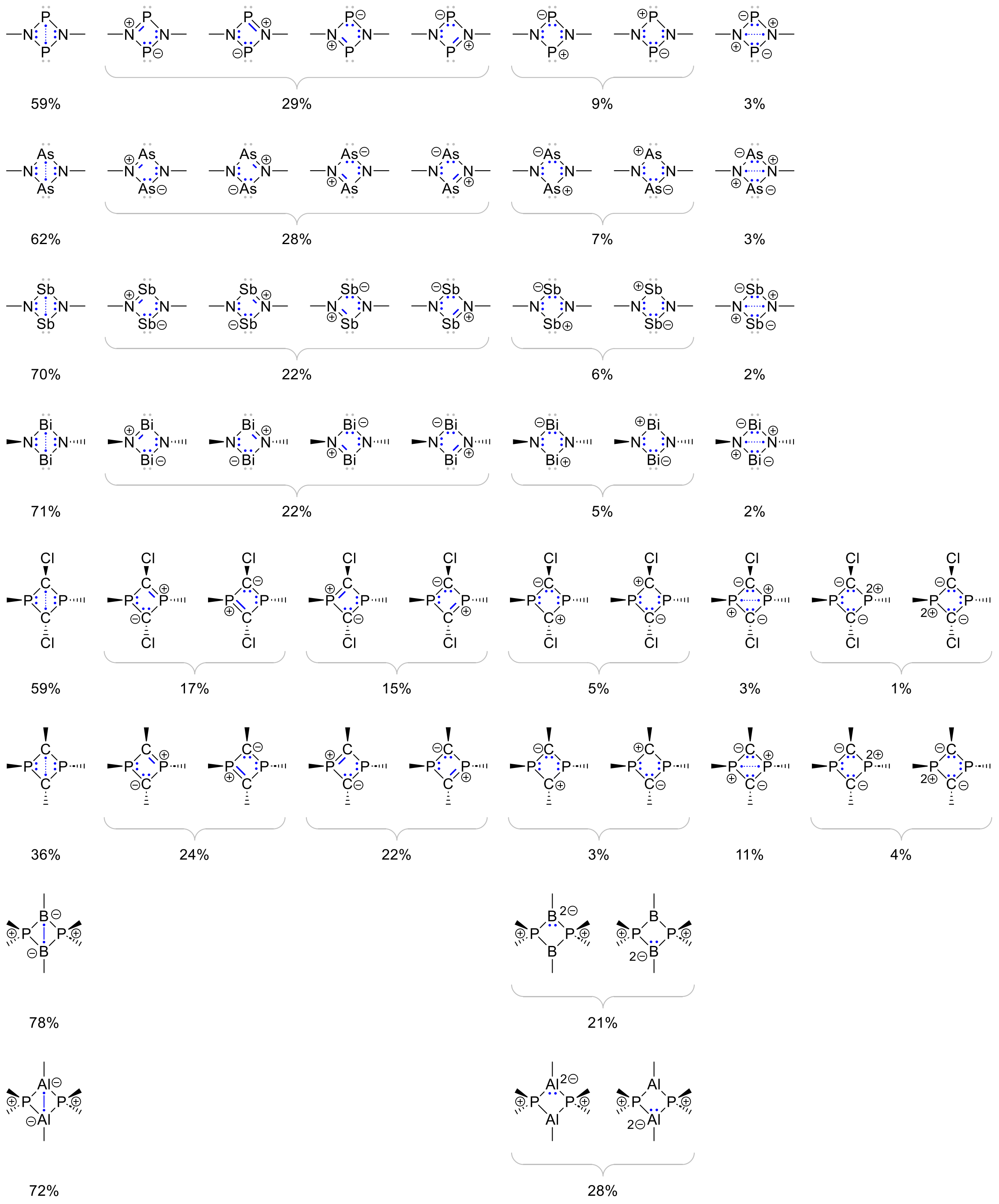

Scheme S1 continued.

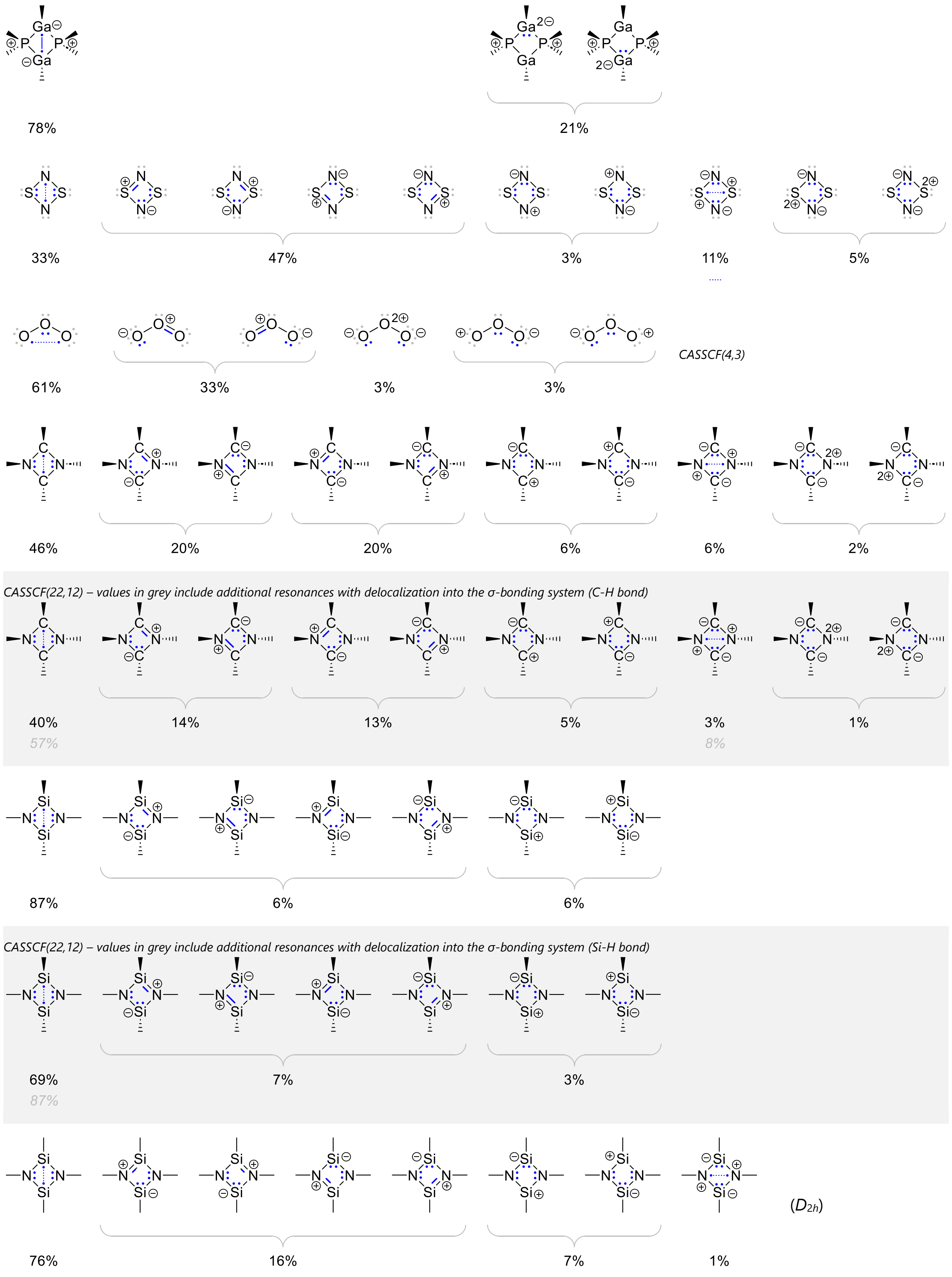

Scheme S1 continued.

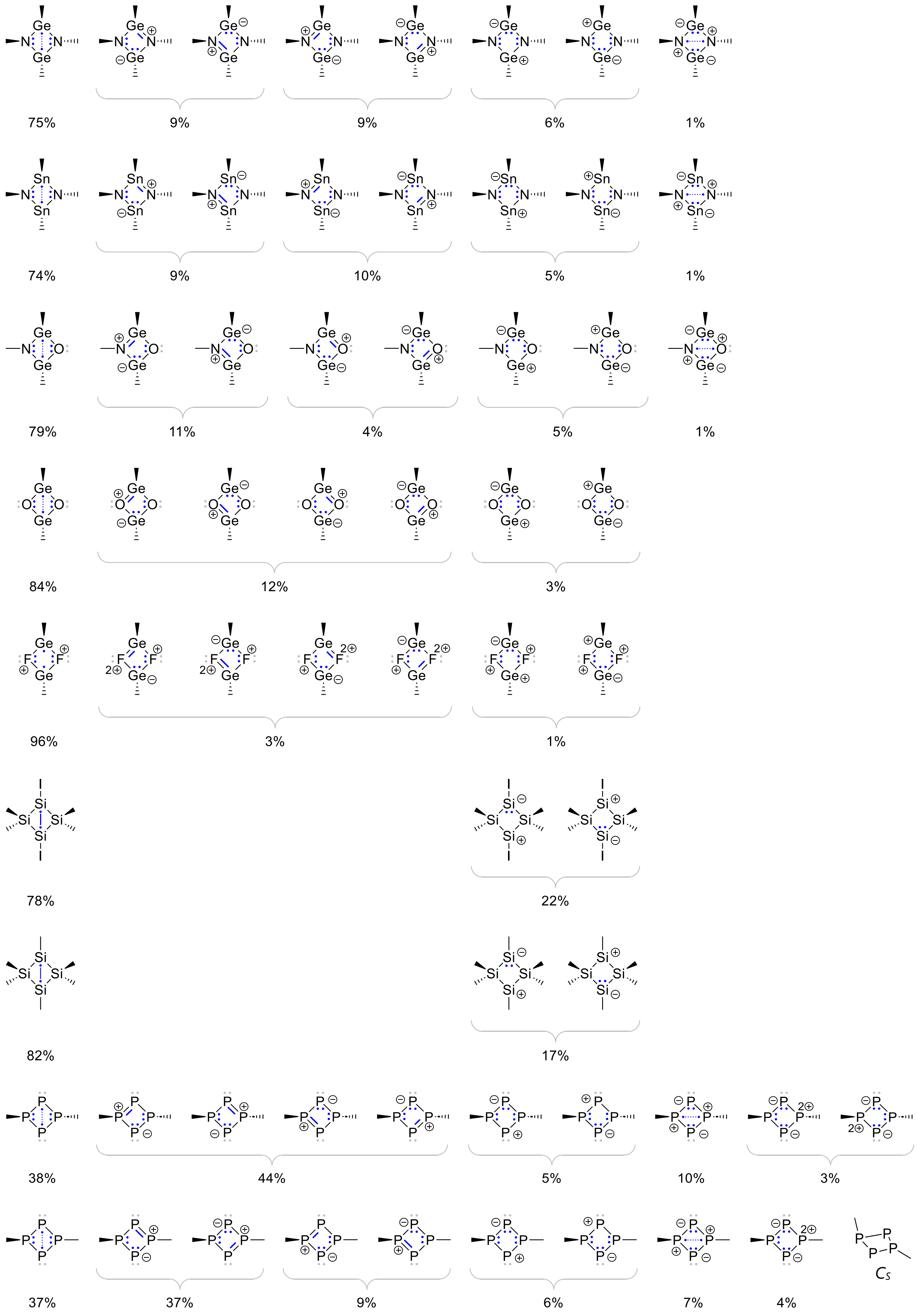

Scheme S1 continued.

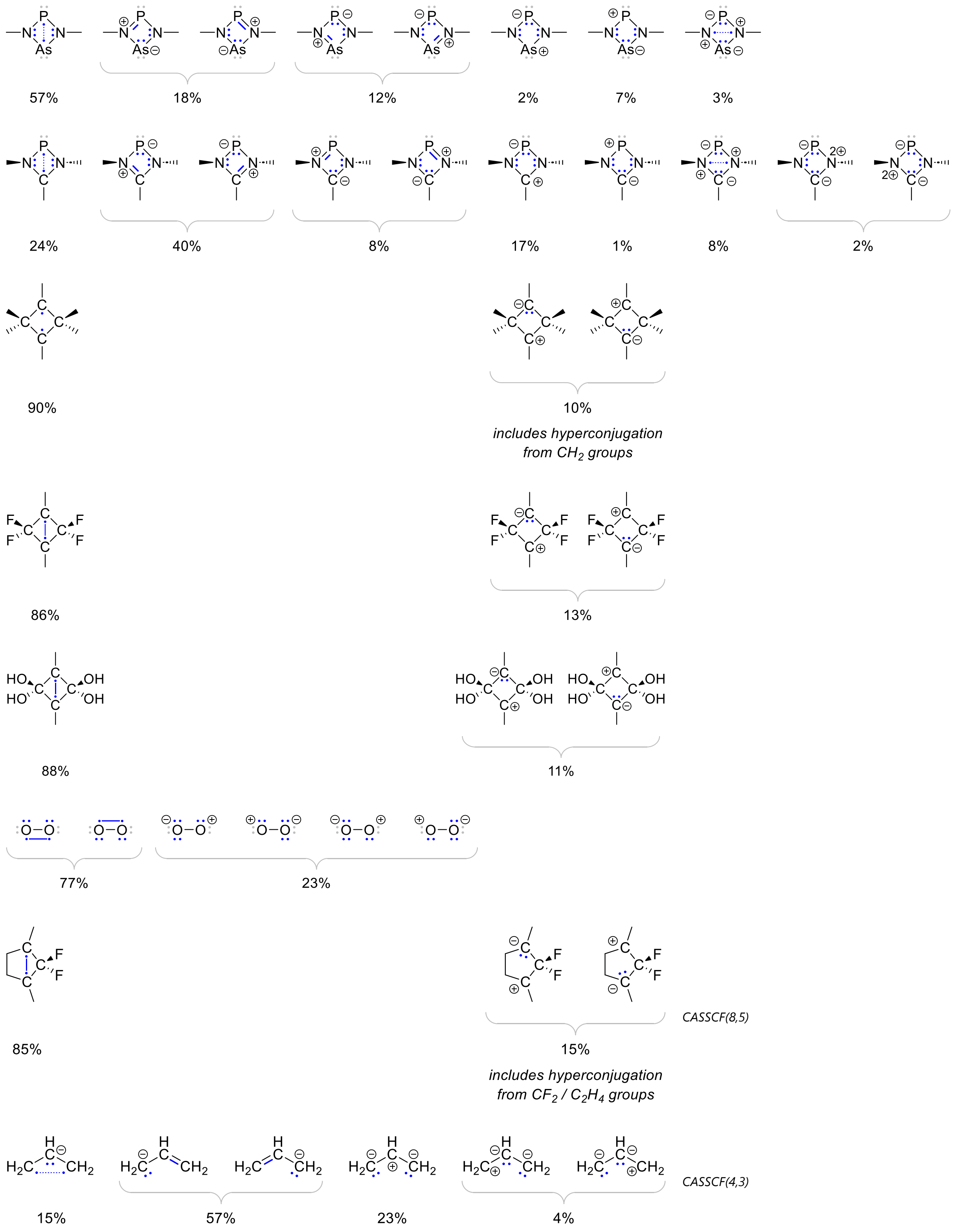

Scheme S2. Lewis-type resonance structures and weights of **$C_2P_2^R$** according to the localization of the CASSCF(6,6)/def2-TZVP wavefunction. Due to extensive delocalization, only the two most important structure types are shown. The weights were summed over several resonance structures of the same type to include delocalization in the C=C bond and/or backbone R. •····• = single π-antibond, •—• = single π-bond (threshold: $|BO/w_i|$ > 0.2). Sums of weights are reported for symmetry equivalent structures.

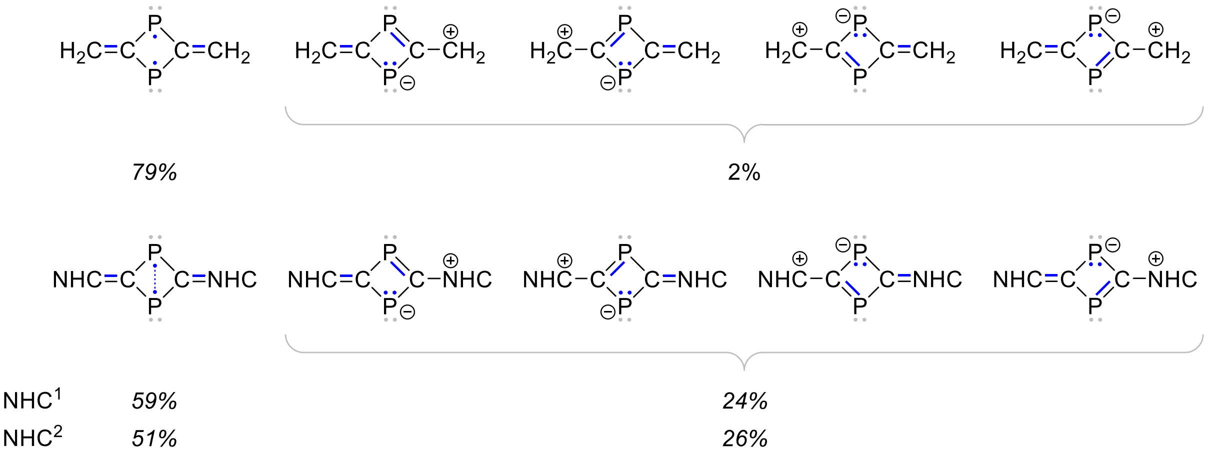

7 Model processes varying the biradical character

In addition to the data points generated from the investigation of the individual model molecules, we wanted to include three continuous processes that vary the biradical character of a molecule. To this end, we chose two classical examples – the dissociation of H_2 and twisting of the double bond of ethylene $H_2C=CH_2$ (cf. Tables S10-S11). However, as these two processes only encompass bonding interactions (either the σ-bond in H_2 or π-bond in ethylene), we included bending of O_3 as a third example (cf. Table S12), since it includes both the bonding and antibonding regime. It is a bit more involved than the first two processes, which is why we quickly discuss it here.

Figure S17. Hyperslice through the PES of ozone. Starting from the equilibrium structure, the OOO angle *a*(OOO) was varied. Using a state-averaged CASSCF(6,4) reference incorporating two formal σ and two formal π orbitals, two singlet and two triplet states were computed at the MRCI+Q/def2-TZVP level of theory. Note this is not a "complete" set of (excited) electronic states due to the limited active space.

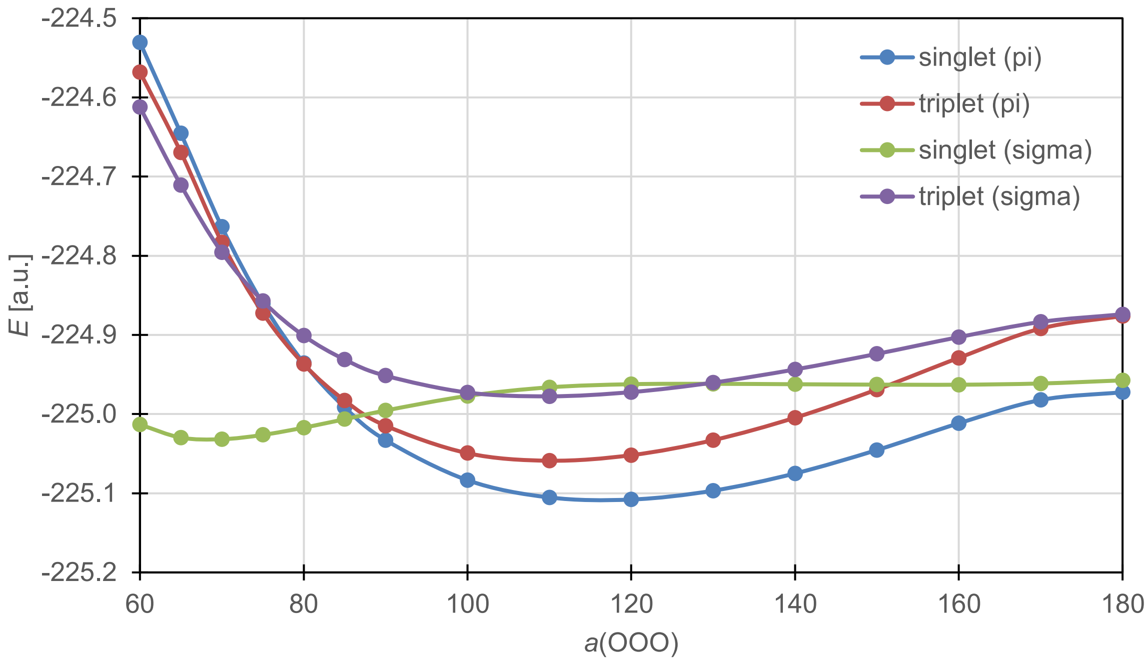

We varied the OOO angle starting from the equilibrium structure at 117.1° (Figure S17). At low angles, a second minimum is found that corresponds to a cyclic isomer of O_3[19] which, however, belongs to a different electronic state with a formal σ-bond between

the terminal O atoms. Hence, the σ* orbital between the two "terminal" O atoms is unoccupied in cyclic ozone. In ozone at its equilibrium structure, however, both the σ and σ* orbitals are doubly occupied, resulting in a net non-bonding situation in the formal σ-bonding system (Figure S18; the two σ-orbitals describe lone pairs at the terminal O atoms). The two singlet states intersect at an angle of ca. 86°. For our study, only the singlet and triplet state involving the π orbitals are relevant (Table S9).

Figure S18. Active space orbitals used for the scan of the OOO angle in ozone, cf. Figure S17. Orbitals plotted at an OOO angle of 90°.

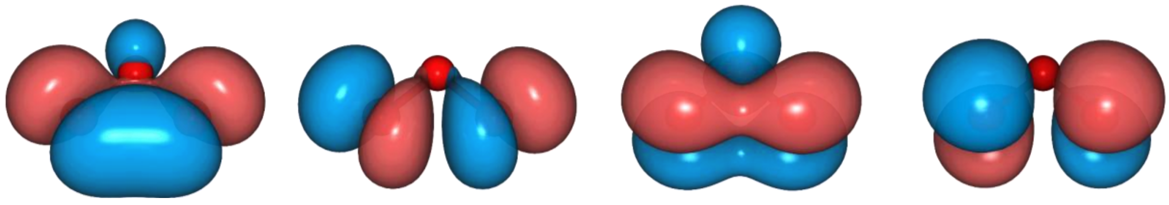

σ (a_1) σ* (b_2) π (b_1) π* (a_2)

Table S9. Relevant natural orbital occupancies depending on the OOO bond angle (MRCI+Q/def2-TZVP).

reference	state-averaged CASSCF(6,4)				state-specific CASSCF(2,2)	
	singlet (σ)		singlet (π)		singlet (π)	
OOO angle [°]	σ (a_1)	σ* (b_2)	π (b_1)	π* (a_2)	π (b_1)	π* (a_2)
60	1.94	0.06	1.38	0.62	1.53	0.48
65	1.92	0.08	1.25	0.75	1.30	0.70
70	1.89	0.10	1.01	0.99	1.01	0.98
75	1.85	0.14	0.81	1.19	0.78	1.22
80	1.80	0.19	0.67	1.33	0.63	1.37
85	1.74	0.26	0.58	1.42	0.53	1.47
90	1.65	0.35	0.51	1.50	0.46	1.54
100	1.41	0.59	0.42	1.58	0.38	1.62
110	1.11	0.88	0.38	1.63	0.34	1.66
120	0.84	1.16	0.34	1.66	0.31	1.69
130	0.63	1.37	0.32	1.68	0.29	1.72
140	0.47	1.53	0.30	1.71	0.27	1.74
150	0.37	1.64	0.29	1.73	0.25	1.75
160	0.33	1.71	0.30	1.75	0.23	1.77

For an accurate description of the singlet state involving the π orbitals, a state-specific MRCI+Q/def2-TZVP energy profile using a CASSCF(2,2) reference was computed. The LUNO occupancy was utilized as singlet biradical character (Table S9); the π bond order was derived from analogous CASSCF(4,3) calculations with localized orbitals.

Table S10. Relevant descriptors of the dissociation of H_2 (MRCI+Q/def2-TZVP; w_{11} and BO: CASSCF(2,2)/def2-TZVP).

r_{HH} [Å]	E_{tot} (S0) [a.u.]	ΔE_{ST} [kJ/mol]	n_{LUNO}	w_{11}	BO
0.5	−1.0974	−1383.0	0.01	0.57	0.99
0.7	−1.1678	−1085.7	0.02	0.60	0.98
0.9	−1.1568	−812.0	0.03	0.64	0.96
1.1	−1.1252	−579.5	0.06	0.68	0.93
1.3	−1.0916	−396.3	0.11	0.74	0.88
1.5	−1.0627	−260.0	0.17	0.79	0.81
1.7	−1.0404	−163.8	0.27	0.85	0.71
1.9	−1.0248	−99.3	0.38	0.90	0.61
2.1	−1.0146	−58.3	0.49	0.93	0.49
2.3	−1.0085	−33.3	0.60	0.96	0.39
2.5	−1.0049	−18.7	0.69	0.98	0.30
2.7	−1.0030	−10.4	0.77	0.99	0.23
2.9	−1.0019	−5.7	0.83	0.99	0.17
3.1	−1.0013	−3.1	0.87	1.00	0.13
3.3	−1.0010	−1.7	0.90	1.00	0.10
3.5	−1.0008	−0.9	0.93	1.00	0.07
3.7	−1.0007	−0.5	0.95	1.00	0.05
3.9	−1.0007	−0.3	0.96	1.00	0.04
4.1	−1.0007	−0.1	0.97	1.00	0.03
4.3	−1.0006	−0.1	0.98	1.00	0.02

Table S11. Relevant descriptors of the torsion of $H_2C=CH_2$ (MRCI+Q/def2-TZVP; w_{11} and BO: CASSCF(6,4)/def2-TZVP).

d_{HCCH} [°]	E_{tot} (S0) [a.u.]	ΔE_{ST} [kJ/mol]	n_{LUNO}	w_{11}	BO
0	−78.4232	−442.4	0.08	0.70	0.92
9	−78.4217	−434.5	0.08	0.70	0.91
18	−78.4171	−410.3	0.08	0.70	0.91
27	−78.4094	−371.5	0.09	0.71	0.90
36	−78.3987	−321.4	0.10	0.72	0.88
45	−78.3854	−262.9	0.11	0.73	0.86
54	−78.3698	−199.8	0.14	0.75	0.82
63	−78.3526	−135.1	0.19	0.78	0.75
72	−78.3358	−69.6	0.32	0.84	0.60
81	−78.3220	−24.6	0.58	0.90	0.35
90	−78.3165	−7.6	0.99	0.93	0.00

Table S12. Relevant descriptors of the bending of O_3 (MRCI+Q/def2-TZVP; w_{11} and BO: CASSCF(4,3)/def2-TZVP).

d_{HCCH} [°]	E_{tot} (S0) [a.u.]	ΔE_{ST} [kJ/mol]	n_{LUNO}	w_{11}	BO
60	−224.5324	98.4	0.48	0.84	0.55
65	−224.6454	62.8	0.70	0.87	0.36
70	−224.7636	51.5	0.98	0.86	0.15
75	−224.8617	31.0	0.78	0.82	−0.03
80	−224.9375	4.0	0.63	0.78	−0.16
85	−224.9942	−22.8	0.53	0.74	−0.24
90	−225.0357	−47.9	0.46	0.71	−0.30
100	−225.0863	−89.6	0.38	0.67	−0.37
110	−225.1078	−121.8	0.34	0.63	−0.40
120	−225.1102	−147.1	0.31	0.60	−0.43
130	−225.0988	−167.5	0.29	0.57	−0.44
140	−225.0769	−184.8	0.27	0.54	−0.45
150	−225.0471	−200.7	0.25	0.50	−0.46
160	−225.0129	−217.1	0.23	0.46	−0.46

Notice that in Figure 9 in the main manuscript, 100% biradical character (based on n_{LUNO}) and BO = 0 do not coincide in the case of bent ozone. Several effects may be at work here: First, the bond order was computed from "simple" CAS(4,3) calculations which do not include dynamical correlation, while n_{LUNO} was computed from MRCI+Q

calculations which do include dynamical correlation effects. As we noticed before, the biradical character of ozone is exceptionally strongly influenced by dynamical correlation, so it is to be expected that dynamical correlation would also influence the bond order. Secondly, since we compute the bond order strictly between the terminal O atoms but the radical electrons are somewhat delocalized (notice how the b_1 orbital in Figure S18 is not only localized on the terminal O atoms but also on the central one), there might actually be an offset between 100% biradical character and zero bond order, if the bonding and antibonding properties of the two active orbitals do not cancel out exactly when they are equally occupied.

8 Derivation of equation 20

Starting from equations 14

$$\Delta E_{\mathrm{ST}} = C + K_{\mathrm{ab}} - \frac{1}{2}\sqrt{\Delta E_{\mathrm{ab}}^2 + 4K_{\mathrm{ab}}^2}$$

and 19

$$\beta = 1 - \frac{\Delta E_{\mathrm{ab}}}{\sqrt{\Delta E_{\mathrm{ab}}^2 + 4K_{\mathrm{ab}}^2}}$$

we define

$$D = \sqrt{\Delta E_{\mathrm{ab}}^2 + 4K_{\mathrm{ab}}^2}$$

for simplicity, which gives

$$\Delta E_{\mathrm{ST}} = C + K_{\mathrm{ab}} - \frac{D}{2}$$

$$\beta = 1 - \frac{\Delta E_{\mathrm{ab}}}{D} \;\Rightarrow\; \Delta E_{\mathrm{ab}} = D(1-\beta)$$

We now substitute $\Delta E_{\mathrm{ab}} = D(1-\beta)$ into $D^2 = \Delta E_{\mathrm{ab}}^2 + 4K_{\mathrm{ab}}^2$:

$$D^2 = D^2(1-\beta)^2 + 4K_{\mathrm{ab}}^2$$

$$D^2 \underbrace{\left[1-(1-\beta)^2\right]}_{\beta(2-\beta)} = 4K_{\mathrm{ab}}^2$$

$$D^2 \cdot \beta(2-\beta) = 4K_{\mathrm{ab}}^2$$

$$D = \frac{2K_{\mathrm{ab}}}{\sqrt{\beta(2-\beta)}}$$

D is now substituted into ΔE_{ST}

$$\Delta E_{\mathrm{ST}} = C + K_{\mathrm{ab}} - \frac{1}{2}\left(\frac{2K_{\mathrm{ab}}}{\sqrt{\beta(2-\beta)}}\right)$$

$$\Delta E_{\mathrm{ST}} = C + K_{\mathrm{ab}}\left(1 - \frac{1}{\sqrt{\beta(2-\beta)}}\right)$$

9 Additional biradical indices

In the main manuscript, we mainly discuss the LUNO occupancy n_{LUNO} from MRCI calculations as biradical indicator. As shown in Figure 5, the LUNO occupancy from a simple CASSCF(2,2) calculation is a reasonable (albeit less accurate) substitute in those instances where MRCI is not feasible. Estimates are also possible using the FOD formalism,[74,75] although it should be noted that this method is somewhat more qualitative (Figure S19, Table S13).

Moreover, we calculated the Yamaguchi and Head-Gordon biradical indices $N_D^{Y}/2$, $N_D^{HG\text{-}I}/2$ and $N_D^{HG\text{-}II}/2$ from the MRCI natural orbital occupancies:[20,76–78]

$$N_{\mathrm{D}}^{\mathrm{Y}} = 2n_{\mathrm{LUNO}}(2 - n_{\mathrm{LUNO}})$$

$$N_{\mathrm{D}}^{\mathrm{HG\text{-}I}} = \sum_i \min\,(n_i, 2 - n_i)$$

$$N_{\mathrm{D}}^{\mathrm{HG\text{-}II}} = \sum_i n_i^2(2 - n_i)^2 \approx \frac{\left(N_{\mathrm{D}}^{\mathrm{Y}}\right)^2}{2}$$

Figure S19. Comparison between different biradical indicators. Left: LUNO occupancy n_{LUNO} (MRCI) vs. $N_{FOD}/2$. Right: LUNO occupancy n_{LUNO} (MRCI) vs. $N_D/2$ as defined by Yamaguchi and Head-Gordon.

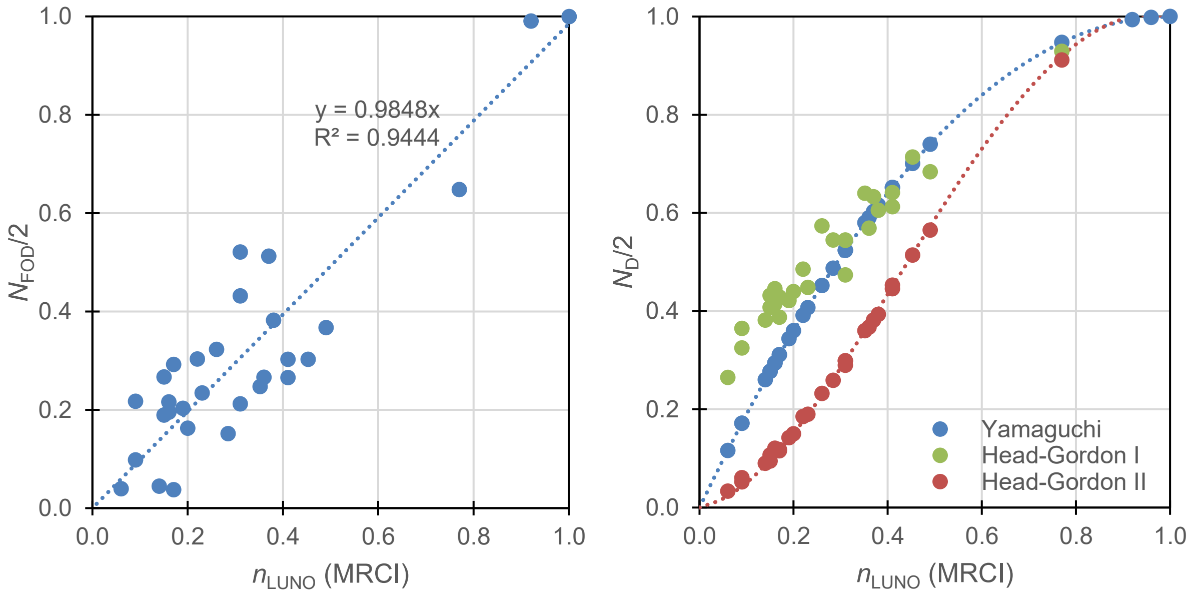

Table S13. Comparison of some additional biradical indices with n_{LUNO} (MRCI).

Compd.	PG	n_{LUNO} (MRCI)	$N_D^Y/2$	$N_D^{HG-I}/2$	$N_D^{HG-II}/2$	$N_{FOD}/2$
**N_2P_2**	D_{2h}	0.20	0.36	0.44	0.15	0.16
**N_2As_2**	C_{2h}	0.23	0.41	0.45	0.19	0.23
**N_2Sb_2**	C_{2h}	0.31	0.52	0.54	0.30	0.43
**N_2Bi_2**	C_{2h}	0.37	0.60	0.63	0.38	0.51
**$P_2C_2^{Cl}$**	C_i	0.26	0.45	0.57	0.23	0.32
**P_2C_2**	C_i	0.16	0.29	0.42	0.12	0.20
**P_2B_2**	D_{2h}	0.16	0.29	0.42	0.12	0.20
**P_2Al_2**	D_{2h}	0.09	0.17	0.36	0.06	0.22
**P_2Ga_2**	C_{2h}	0.17	0.31	0.43	0.12	0.29
**S_2N_2**	D_{2h}	0.14	0.26	0.38	0.09	0.04
**O_3**	C_{2v}	0.31	0.52	0.47	0.29	0.21
**N_2C_2**	C_i	0.17	0.31	0.39	0.12	0.04
**N_2Si_2**	C_{2h}	0.41	0.65	0.64	0.45	0.27
**N_2Si_2**	D_{2h}	0.31	0.52	0.55	0.30	0.52
**N_2Ge_2**	C_i	0.36	0.59	0.57	0.37	0.27
**N_2Sn_2**	C_i	0.38	0.62	0.61	0.39	0.38
**$NOGe_2$**	C_2	0.41	0.65	0.61	0.45	0.30
**O_2Ge_2**	C_{2h}	0.49	0.74	0.68	0.57	0.37
**$F_2Ge_2^{2+}$**	C_{2h}	0.77	0.95	0.93	0.91	0.65
**$Si_2Si_2^{I}$**	D_{2h}	0.15	0.28	0.41	0.09	0.27
**Si_2Si_2**	D_{2h}	0.22	0.39	0.49	0.19	0.30
**P_2P_2**	C_{2h}	0.16	0.29	0.45	0.12	0.22
**P_2P_2**	C_s	0.15	0.28	0.43	0.11	0.19
**N_2PAs**	C_2	0.19	0.34	0.42	0.14	0.20
**N_2CP**	C_2	0.09	0.17	0.32	0.05	0.10
**$C_2P_2^{CH_2}$**	D_{2h}	0.96	1.00	1.26	1.02	1.05
**O_2**	$D_{\infty h}$	1.00	1.00	1.11	1.01	1.00
**C_2C_2**	D_{2h}	0.92	0.99	1.15	1.01	0.99
**$C_2C_2^{F}$**	D_{2h}	0.06	0.12	0.27	0.03	0.04
**$C_2C_2^{OH}$**	D_2	0.28	0.49	0.54	0.26	0.15
**$C_3C_2^{F}$**	C_{2v}	0.45	0.70	0.71	0.51	0.30
**$Allyl^{-}$**	C_{2v}	0.35	0.58	0.64	0.36	0.25

10 References

[1] *Gaussian 09, Revision E.01*, M. J. Frisch, G. W. Trucks, H. B. Schlegel, G. E. Scuseria, M. A. Robb, J. R. Cheeseman, G. Scalmani, V. Barone, B. Mennucci, G. A. Petersson, H. Nakatsuji, M. Caricato, X. Li, H. P. Hratchian, A. F. Izmaylov, J. Bloino, G. Zheng, J. L. Sonnenberg, M. Hada, M. Ehara, K. Toyota, R. Fukuda, J. Hasegawa, M. Ishida, T. Nakajima, Y. Honda, O. Kitao, H. Nakai, T. Vreven, J. A. Montgomery Jr., J. E. Peralta, F. Ogliaro, M. Bearpark, J. J. Heyd, E. Brothers, K. N. Kudin, V. N. Staroverov, T. Keith, R. Kobayashi, J. Normand, K. Raghavachari, A. Rendell, J. C. Burant, S. S. Iyengar, J. Tomasi, M. Cossi, N. Rega, J. M. Millam, M. Klene, J. E. Knox, J. B. Cross, V. Bakken, C. Adamo, J. Jaramillo, R. Gomperts, R. E. Stratmann, O. Yazyev, A. J. Austin, R. Cammi, C. Pomelli, J. W. Ochterski, R. L. Martin, K. Morokuma, V. G. Zakrzewski, G. A. Voth, P. Salvador, J. J. Dannenberg, S. Dapprich, A. D. Daniels, O. Farkas, J. B. Foresman, J. V. Ortiz, J. Cioslowski, D. J. Fox, Gaussian, Inc., Wallingford CT, **2013**.

[2] *Gaussian 16, Revision C.02*, M. J. Frisch, G. W. Trucks, H. B. Schlegel, G. E. Scuseria, M. A. Robb, J. R. Cheeseman, G. Scalmani, V. Barone, G. A. Petersson, H. Nakatsuji, X. Li, M. Caricato, A. V. Marenich, J. Bloino, B. G. Janesko, R. Gomperts, B. Mennucci, H. P. Hratchian, J. V. Ortiz, A. F. Izmaylov, J. L. Sonnenberg, D. Williams-Young, F. Ding, F. Lipparini, F. Egidi, J. Goings, B. Peng, A. Petrone, T. Henderson, D. Ranasinghe, V. G. Zakrzewski, J. Gao, N. Rega, G. Zheng, W. Liang, M. Hada, M. Ehara, K. Toyota, R. Fukuda, J. Hasegawa, M. Ishida, T. Nakajima, Y. Honda, O. Kitao, H. Nakai, T. Vreven, K. Throssell, J. A. Montgomery, Jr., J. E. Peralta, F. Ogliaro, M. J. Bearpark, J. J. Heyd, E. N. Brothers, K. N. Kudin, V. N. Staroverov, T. A. Keith, R. Kobayashi, J. Normand, K. Raghavachari, A. P. Rendell, J. C. Burant, S. S. Iyengar, J. Tomasi, M. Cossi, J. M. Millam, M. Klene, C. Adamo, R. Cammi, J. W. Ochterski, R. L. Martin, K. Morokuma, O. Farkas, J. B. Foresman, and D. J. Fox, Gaussian, Inc., Wallingford CT, **2019**.

[3] F. Neese, *WIREs Comput. Mol. Sci.* **2012**, *2*, 73–78.

[4] F. Neese, *WIREs Comput. Mol. Sci.* **2018**, *8*, e1327.

[5] F. Neese, *WIREs Comput. Mol. Sci.* **2022**, *12*, e1606.

[6] F. Neese, F. Wennmohs, U. Becker, C. Riplinger, *J. Chem. Phys.* **2020**, *152*, 224108.

[7] E. D. Glendening, J. K. Badenhoop, A. E. Reed, J. E. Carpenter, J. A. Bohmann, C. M. Morales, C. R. Landis, F. Weinhold, *NBO 6.0*, Theoretical Chemistry Institute, University of Wisconsin, Madison, **2013**.

[8] J. E. Carpenter, F. Weinhold, *J. Mol. Struct. THEOCHEM* **1988**, *169*, 41–62.

[9] F. Weinhold, J. E. Carpenter, in *The Structure of Small Molecules and Ions* (Eds.: R. Naaman, Z. Vager), Springer, Boston, MA, **1988**, pp. 227–236.

[10] F. Weinhold, C. R. Landis, *Valency and Bonding. A Natural Bond Orbital Donor-Acceptor Perspective*, Cambridge University Press, **2005**.

[11] J. P. Perdew, K. Burke, M. Ernzerhof, *Phys. Rev. Lett.* **1996**, *77*, 3865–3868.

[12] J. P. Perdew, K. Burke, M. Ernzerhof, *Phys. Rev. Lett.* **1997**, *78*, 1396–1396.

[13] S. Grimme, J. Antony, S. Ehrlich, H. Krieg, *J. Chem. Phys.* **2010**, *132*, 154104.

[14] S. Grimme, S. Ehrlich, L. Goerigk, *J. Comput. Chem.* **2011**, *32*, 1456–1465.

[15] F. Weigend, R. Ahlrichs, *Phys. Chem. Chem. Phys.* **2005**, *7*, 3297–305.

[16] F. Weigend, *Phys. Chem. Chem. Phys.* **2006**, *8*, 1057.

[17] J. Bresien, D. Michalik, A. Schulz, A. Villinger, E. Zander, *Angew. Chem. Int. Ed.* **2021**, *60*, 1507–1512.

[18] T. Völzer, H. Beer, A. Schulz, S. Lochbrunner, J. Bresien, *Phys. Chem. Chem. Phys.* **2021**, *23*, 7434–7441.

[19] A. Hinz, J. Bresien, F. Breher, A. Schulz, *Chem. Rev.* **2023**, *123*, 10468–10526.

[20] T. Stuyver, B. Chen, T. Zeng, P. Geerlings, F. De Proft, R. Hoffmann, *Chem. Rev.* **2019**, *119*, 11291–11351.

[21] D. Herebian, K. E. Wieghardt, F. Neese, *J. Am. Chem. Soc.* **2003**, *125*, 10997–11005.

[22] C. J. Cramer, *Essentials of Computational Chemistry: Theories and Models*, John Wiley & Sons, Ltd, Chichester, UK, **2004**.

[23] J. Čížek, in *Advances in Chemical Physics* (Eds.: R. LeFebvre, C. Moser), John Wiley & Sons, **1969**, pp. 35–89.

[24] R. J. Bartlett, G. D. Purvis, *Int. J. Quantum Chem.* **1978**, *14*, 561–581.

[25] G. E. Scuseria, C. L. Janssen, H. F. Schaefer, *J. Chem. Phys.* **1988**, *89*, 7382–7387.

[26] G. D. Purvis, R. J. Bartlett, *J. Chem. Phys.* **1982**, *76*, 1910–1918.

[27] J. A. Pople, M. Head-Gordon, K. Raghavachari, *J. Chem. Phys.* **1987**, *87*, 5968–5975.

[28] T. J. Lee, P. R. Taylor, *Int. J. Quantum Chem.* **1989**, *36*, 199–207.

[29] D. Jayatilaka, T. J. Lee, *J. Chem. Phys.* **1993**, *98*, 9734–9747.

[30] T. J. Lee, *Chem. Phys. Lett.* **2003**, *372*, 362–367.

[31] G. Das, A. C. Wahl, *J. Chem. Phys.* **1966**, *44*, 87–96.

[32] B. O. Roos, *Int. J. Quantum Chem.* **1980**, *18*, 175–189.

[33] B. O. Roos, P. R. Taylor, P. E. M. Sigbahn, *Chem. Phys.* **1980**, *48*, 157–173.

[34] B. O. Roos, R. Lindh, P. Å. Malmqvist, V. Veryazov, P. Widmark, *Multiconfigurational Quantum Chemistry*, John Wiley & Sons, Inc., Hoboken, New Jersey, **2016**.

[35] A. Hellweg, C. Hättig, S. Höfener, W. Klopper, *Theor. Chem. Acc.* **2007**, *117*, 587–597.

[36] C. Angeli, R. Cimiraglia, J.-P. Malrieu, *Chem. Phys. Lett.* **2001**, *350*, 297–305.

[37] C. Angeli, R. Cimiraglia, S. Evangelisti, T. Leininger, J.-P. Malrieu, *J. Chem. Phys.*

2001, *114*, 10252–10264.
[38] C. Angeli, R. Cimiraglia, J.-P. Malrieu, *J. Chem. Phys.* **2002**, *117*, 9138–9153.
[39] S. R. Langhoff, E. R. Davidson, *Int. J. Quantum Chem.* **1974**, *8*, 61–72.
[40] L. Meissner, *Chem. Phys. Lett.* **1988**, *146*, 204–210.
[41] M. Goehring, D. Voigt, *Naturwissenschaften* **1953**, *40*, 482–482.
[42] M. Goehring, D. Voigt, *Z. Anorg. Allg. Chem.* **1956**, *285*, 181–190.
[43] K. Takeuchi, M. Ichinohe, A. Sekiguchi, *J. Am. Chem. Soc.* **2011**, *133*, 12478–12481.
[44] S.-H. Zhang, H.-W. Xi, K. H. Lim, Q. Meng, M.-B. Huang, C.-W. So, *Chem. Eur. J.* **2012**, *18*, 4258–4263.
[45] S. Demeshko, C. Godemann, R. Kuzora, A. Schulz, A. Villinger, *Angew. Chem. Int. Ed.* **2013**, *52*, 2105–2108.
[46] D. Rottschäfer, B. Neumann, H.-G. Stammler, R. S. Ghadwal, *Chem. Eur. J.* **2017**, *23*, 9044–9047.
[47] Z. Li, X. Chen, D. M. Andrada, G. Frenking, Z. Benkő, Y. Li, J. R. Harmer, C.-Y. Su, H. Grützmacher, *Angew. Chem. Int. Ed.* **2017**, *56*, 5744–5749.
[48] E. Niecke, A. Fuchs, F. Baumeister, M. Nieger, W. W. Schoeller, *Angew. Chem. Int. Ed. Engl.* **1995**, *34*, 555–557.
[49] D. Scheschkewitz, H. Amii, H. Gornitzka, W. W. Schoeller, D. Bourissou, G. Bertrand, *Science* **2002**, *295*, 1880–1881.
[50] M. Abe, C. Ishihara, M. Nojima, *J. Org. Chem.* **2003**, *68*, 1618–1621.
[51] H. Cox, P. B. Hitchcock, M. F. Lappert, L. J. M. Pierssens, *Angew. Chem. Int. Ed.* **2004**, *43*, 4500–4504.
[52] C. Cui, M. Brynda, M. M. Olmstead, P. P. Power, *J. Am. Chem. Soc.* **2004**, *126*, 6510–6511.
[53] P. Henke, T. Pankewitz, W. Klopper, F. Breher, H. Schnöckel, *Angew. Chem. Int. Ed.* **2009**, *48*, 8141–8145.
[54] X. Wang, Y. Peng, M. M. Olmstead, J. C. Fettinger, P. P. Power, *J. Am. Chem. Soc.* **2009**, *131*, 14164–14165.
[55] T. Beweries, R. Kuzora, U. Rosenthal, A. Schulz, A. Villinger, *Angew. Chem. Int. Ed.* **2011**, *50*, 8974–8978.
[56] A. Hinz, A. Schulz, A. Villinger, *Angew. Chem. Int. Ed.* **2015**, *54*, 668–672.
[57] T. Suhrbier, J. Bresien, A. Villinger, A. Schulz, *Cell Rep. Phys. Sci.* **2022**, *3*, 100777.
[58] T. Nukazawa, T. Iwamoto, *J. Am. Chem. Soc.* **2020**, *142*, 9920–9924.
[59] R. A. Kendall, T. H. Dunning Jr., R. J. Harrison, *J. Chem. Phys.* **1992**, *96*, 6796–6806.
[60] R. Bauernschmitt, R. Ahlrichs, *Chem. Phys. Lett.* **1996**, *256*, 454–464.
[61] C. Van Caillie, R. D. Amos, *Chem. Phys. Lett.* **1999**, *308*, 249–255.

[62] C. Van Caillie, R. D. Amos, *Chem. Phys. Lett.* **2000**, *317*, 159–164.

[63] F. Furche, R. Ahlrichs, *J. Chem. Phys.* **2002**, *117*, 7433–7447.

[64] F. Neese, *J. Am. Chem. Soc.* **2006**, *128*, 10213–10222.

[65] I. Sandler, J. Chen, M. Taylor, S. Sharma, J. Ho, *J. Phys. Chem. A* **2021**, *125*, 1553–1563.

[66] L. Salem, C. Rowland, *Angew. Chem. Int. Ed. Engl.* **1972**, *11*, 92–111.

[67] W. C. Lineberger, W. T. Borden, *Phys. Chem. Chem. Phys.* **2011**, *13*, 11792.

[68] J. Bresien, L. Eickhoff, A. Schulz, E. Zander, in *Comprehensive Inorganic Chemistry III* (Eds.: J. Reedijk, K. Poeppelmeier), Elsevier, **2023**, pp. 165–233.

[69] E. Miliordos, K. Ruedenberg, S. S. Xantheas, *Angew. Chem. Int. Ed.* **2013**, *52*, 5736–5739.

[70] F. Weigend, A. Köhn, C. Hättig, *J. Chem. Phys.* **2002**, *116*, 3175–3183.

[71] J. M. Foster, S. F. Boys, *Rev. Mod. Phys.* **1960**, *32*, 300–302.

[72] D. A. Kleier, T. A. Halgren, J. H. Hall, W. N. Lipscomb, *J. Chem. Phys.* **1974**, *61*, 3905–3919.

[73] J.-P. Malrieu, G. Trinquier, *J. Phys. Chem. A* **2012**, *116*, 8226–8237.

[74] S. Grimme, A. Hansen, *Angew. Chem. Int. Ed.* **2015**, *54*, 12308–12313.

[75] C. A. Bauer, A. Hansen, S. Grimme, *Chem. – A Eur. J.* **2017**, *23*, 6150–6164.

[76] M. Head-Gordon, *Chem. Phys. Lett.* **2003**, *372*, 508–511.

[77] M. Head-Gordon, *Chem. Phys. Lett.* **2003**, *380*, 488–489.

[78] K. Yamaguchi, *Chem. Phys. Lett.* **1975**, *33*, 330–335.
